# First-in-human, phase 1 dose-escalation and dose-expansion study of a RET inhibitor SY-5007 in patients with advanced *RET*-altered solid tumors

**DOI:** 10.1038/s41392-024-02006-9

**Published:** 2024-11-04

**Authors:** Wei Li, Yongsheng Wang, Anwen Xiong, Ge Gao, Zhengbo Song, Yiping Zhang, Dingzhi Huang, Feng Ye, Qiming Wang, Zhihui Li, Jiaye Liu, Chunwei Xu, Yinghui Sun, Xijie Liu, Fei Zhou, Caicun Zhou

**Affiliations:** 1 Department of Medical Oncology, Shanghai Pulmonary Hospital, Tongji University Medical School Cancer Institute, Tongji University School of Medicine, Shanghai, China; 2 Department of Thoracic Oncology, State Key Laboratory of Biotherapy and Cancer Center, West China Hospital, Sichuan University and Collaborative Innovation Center, Chengdu, China; 3 Department of Thoracic Oncology, Zhejiang Cancer Hospital, Hangzhou, China; 4 Lung Cancer Department, Tianjin Medical University Cancer Institute & Hospital, Tianjin, China; 5 Department of Medical Oncology, The First Affiliated Hospital of Xiamen University, Fujian, China; 6 Department of Internal Medicine, The Affiliated Cancer Hospital of Zhengzhou University, Henan Cancer Hospital, Zhengzhou, China; 7 Shouyao Holdings (Beijing) Co., Ltd, Beijing, China

**Keywords:** Clinical trials, Drug development

## Abstract

Oncogenic *RET* alteration is an important, tissue-agnostic therapeutic target across diverse cancers. We conducted a first-in-human phase 1 study on SY-5007, a potent and selective RET inhibitor, in patients with *RET*-altered solid tumors. Primary endpoints were safety, maximum tolerated dose (MTD), and recommended phase 2 dose (RP2D). Secondary endpoints included pharmacokinetics and preliminary anti-tumor activity. A total of 122 patients were enrolled (17 in dose-escalation phase and 105 in dose-expansion phase), including 91 with non-small cell lung cancer, 23 with medullary thyroid cancer, 7 with papillary thyroid cancer and 1 with gastric cancer. Treatment-related adverse events (TRAEs) were reported in 96.7% of patients, with the most common grade ≥ 3 TRAEs being hypertension (22.1%), diarrhea (16.4%), hypertriglyceridemia (6.6%), and neutropenia (6.6%). The exposure to SY-5007 was dose proportional. Among the 116 efficacy-evaluable patients, the overall objective response rate (ORR) was 57.8%, with 70.0% in treatment-naïve patients and 51.3% in previously treated patients. The median progression-free survival (PFS) was 21.1 months. Efficacy was observed regardless of tumor types and previous therapies. Biomarker analysis of 61 patients with circulating tumor DNA (ctDNA)-detectable *RET* alterations showed an ORR of 57.4% and median PFS of 13.8 months. Rapid ctDNA clearance of *RET* alteration correlated with faster responses and improved outcomes. In relapsed patients, off-target induced resistance was observed in 57.1% (12/21), with no on-target *RET* alterations identified. In conclusion, SY-5007 was well-tolerated and showed promising efficacy in patients with *RET*-altered solid tumors. Serial ctDNA monitoring may unveil treatment response and potential resistance mechanisms (NCT05278364).

## Introduction

The REarranged during Transfection (*RET*) gene encodes a vital transmembrane receptor tyrosine kinase that plays an essential role in various physiological processes. Upon stimulation by glial cell line-derived neurotrophic factor (GDNF) family ligands, specific tyrosine residues within the RET intracellular domain undergo autophosphorylation, creating docking sites for key adaptor proteins. This process triggers critical signaling pathways, including RAS/MAPK, PI3K/AKT, and JAK/STAT, which are integral to cellular functions such as migration, proliferation, survival, and differentiation. These pathways are particularly important during embryogenesis, nervous system development, and renal morphogenesis.^1,2^ Dysregulation of RET through somatic gene alterations, such as fusions and activating mutations, can lead to its constitutive activation, which result in heightened downstream signaling activation. This persistent activation over-activates downstream signaling, promoting uncontrolled cell growth, resistance to apoptosis, and ultimately contributing to tumorigenesis and metastasis. Consequently, RET has garnered considerable attention as a significant target for therapeutic intervention in various malignancies.^3–5^

In non-small cell lung cancer (NSCLC), *RET* fusions are found in approximately 1–2% of cases. These fusions typically result from the juxtaposition of the *RET* gene with other genes, such as *KIF5B*, *CCDC6*, and *NCOA4*, producing fusion proteins that exhibit constitutive kinase activity. This persistent activation promotes increased cell proliferation, survival, and invasion, contributing to the aggressive nature of *RET* fusion-positive NSCLC. Such fusions mark a distinct molecular subtype that is associated with poor clinical outcomes and aggressive tumor behavior.^6,7^ In medullary thyroid cancer (MTC), activating *RET* mutations are prevalent in approximately 50% of sporadic cases and nearly all familial cases, correlating with aggressive tumor phenotypes and poor prognosis.^2,8,9^ Similarly, in papillary thyroid carcinoma (PTC), *RET* fusions occur in 10–20% of cases, leading to enhanced kinase activity and invasive growth, further establishing RET as a key driver in thyroid malignancies.^10–12^ Emerging evidence also links *RET* alterations to other solid tumors, including pancreatic and breast cancers, underscoring RET’s broad implications in oncogenesis and the potential for advancing personalized treatment strategies across various solid tumors.^3,13–15^

Several multi-kinase inhibitors (MKIs) have been developed to target RET and its related pathways, including cabozantinib, vandetanib, lenvatinib, and sorafenib. While these agents demonstrate some anti-RET activity in patients with *RET*-altered tumors, the clinical benefits are often modest. This limitation may be due to the constrained anti-RET efficacy and the emergence of dose-limiting off-target toxic effects, such as hypertension and diarrhea, which can significantly affect patient quality of life and adherence to treatment regimens.^14,16–18^ More recently, two highly potent selective RET inhibitors, selpercatinib and pralsetinib, have shown improved efficacy with higher response rates and improved progression-free survival (PFS) compared to traditional MKIs and more favorable toxicity profiles in patients with advanced *RET*-altered solid tumors by specifically targeting RET while minimizing off-target effects, thus enhancing therapeutic outcomes.^19–22^ Currently, both selpercatinib and pralsetinib have received U.S. Food and Drug Administration (FDA) approval for the treatment of *RET* fusion-positive NSCLC and PTC and *RET*-mutant MTC, establishing them as the new standard of care for these patients. Additionally, selpercatinib has tumor-agnostic FDA approval for any solid tumor with a *RET* gene fusion.^23,24^ This highlights the growing recognition of RET as a critical therapeutic target and the importance of personalized medicine approaches in oncology.

SY-5007 is a novel, ATP-competitive small molecule inhibitor that selectively targets RET.^25^ In vitro kinase assays demonstrated that SY-5007 exhibits sub-nanomolar inhibitory activity against RET kinases, including both wild-type RET and prevalent mutations associated with resistance to MKIs,^26^ such as RET^M918T^ (commonly observed in thyroid cancer), RET^V804M^ (a gatekeeper mutation), and RET^G810S^ (a solvent front mutation). Importantly, SY-5007 showed high selectivity against VEGFR2, an off-target kinase that is responsible for cardiovascular adverse effects often seen with non-selective anti-RET therapies (Supplementary Table 1). Additionally, SY-5007 exhibited potent anti-proliferative activity in the low nanomolar range across a variety of *RET*-fusion or mutant-driven cell lines, including TT cells (a human MTC cell line with RET^C634W^ mutation), HEK293T-KIF5B-RET^WT/ V804M/ M918T^ cells, Baf3-KIF5B-RET^WT/ V804M/ M918T^ cells and Baf3-CCDC6-RET^WT/ V804M/ M918T^ cells, while demonstrating minimal impact on the proliferation of normal cells (NIH-3T3) (Supplementary Table 2). In vivo studies further support these findings, revealing dose-dependent and potent anti-tumor activity in various *RET*-driven xenografts models in mice. A minimal effective dose of 10 mg/kg administered twice daily (BID) resulted in significant tumor growth inhibition in most models, with near-complete inhibition observed at 40 mg/kg BID across all models (Supplementary Fig. 1). Collectively, the strong inhibitory potency, broad-spectrum RET inhibition, and high selectivity of SY-5007 suggest that it may enhance RET inhibition efficacy while minimizing off-target toxicities and overcoming resistance mechanisms observed with earlier treatments. The development of SY-5007 could broaden therapeutic options, providing a new, effective treatment pathway and improving clinical outcomes for patients with *RET*-altered tumors. Here, we present the safety, tolerability and preliminary anti-tumor activity of SY-5007 in patients with *RET*-altered solid tumors from a first-in human, multicenter, open-label, phase 1 dose-escalation and dose-expansion study.

## Results

### Patient characteristics

Between April 23, 2021, and November 30, 2023, a total of 122 patients were enrolled in the phase 1 study, with 17 patients in the dose-escalation phase and 105 patients in the dose-expansion phase. Among these patients, 91 had *RET* fusion-positive NSCLC, 23 had *RET*-mutant MTC, 7 had *RET* fusion-positive PTC, and 1 had *RET*-mutant gastric cancer. For prior treatment, 36.1% (44/122) of patients were treatment-naïve and 63.9% (78/122) of patients were previously treated, including 43.4% (53/122) with chemotherapy, 23.8% (29/122) with MKIs, 16.4% (20/122) with programmed death-1 (PD-1)/programmed death ligand-1 (PD-L1) inhibitors, and 27.9% (33/122) with other systemic therapies. *RET* fusions were identified by next-generation sequencing (NGS) in 46.7% (57/122) of patients, reverse transcription-polymerase chain reaction (RT-PCR) in 24.6% (30/122) of patients, and other methods, including fluorescence in situ hybridization (FISH) and immunohistochemistry (IHC) in 28.7% (35/122) of patients. The most prevalent fusion partners were *KIF5B* (54.1%, 66/122) and *CCDC6* (15.6%, 19/122). The most common *RET* mutation was *RET*^M918T^ (12.3%, 15/122). Detailed patient demographics and baseline characteristics are summarized in Table 1 and Supplementary Table 3.

**Table 1:** Baseline characteristics of patients received SY-5007 in phase 1 trial

	*RET*-altered Solid Tumors (*n* = 122)	Dose-escalation phase (*n* = 17)	Dose-expansion phase (*n* = 105)
Median age (range)	57.5 (28–79)	59.0 (32–73)	57.0 (28-79)
Sex, n (%)			
Male	56 (45.9)	5 (29.4)	51 (48.6)
Female	66 (54.1)	12 (70.6)	54 (51.4)
Cancer type, *n* (%)			
NSCLC	91 (74.6)	16 (94.1)	75 (71.4)
MTC	23 (18.9)	0 (0.0)	23 (21.9)
PTC	7 (5.7)	0 (0.0)	7 (6.7)
GC	1 (0.8)	1 (5.9)	0 (0.0)
ECOG PS, *n* (%)^a^			
0	30 (24.6)	1 (5.9)	29 (27.6)
1	92 (75.4)	16 (94.1)	76 (72.4)
Smoking history, *n* (%)			
Current/Prior	30 (24.6)	2 (28.6)	28 (26.7)
Never	92 (75.4)	15 (88.2)	77 (73.3)
Brain metastases, *n* (%)	25 (20.5)	4 (23.5)	21 (20.0)
*RET*-testing method, *n* (%)^b^			
NGS	57 (46.7)	4 (23.5)	53 (50.5)
RT-PCR	30 (24.6)	10 (58.8)	20 (19.0)
Other^c^	35 (28.7)	3 (17.6)	32 (30.5)
*RET* fusion, *n* (%)			
*KIF5B-RET*	66 (54.1)	9 (52.9)	57 (54.2)
*CCDC6-RET*	19 (15.6)	4 (23.5)	15 (14.2)
*NCOA4-RET*	4 (3.3)	0 (0.0)	4 (3.8)
Other^d^	6 (4.9)	1 (5.8)	5 (4.8)
Unknown^e^	5 (4.1)	2 (11.8)	3 (2.6)
*RET* mutation			
M918T	15 (12.3)	0 (0.0)	15 (14.3)
Other^f^	8 (6.6)	0 (0.0)	8 (7.6)
Treatment naïve, *n* (%)	44 (36.1)	1 (5.9)	43 (41.0)
Prior systemic therapy, *n* (%)			
Chemotherapy	53 (43.4)	15 (88.2)	38 (36.2)
Multi-kinase inhibitor	29 (23.8)	0 (0.0)	29 (27.6)
PD-(L)1 inhibitor	20 (16.4)	4 (23.5)	16 (15.2)
Others^g^	33 (27.0)	11 (64.7)	22 (21.0)
Prior cancer-related surgery, *n* (%)	33 (27.0)	3 (17.6)	30 (28.6)

*ECOG PS* Eastern Cooperative Oncology Group performance status, *GC* gastric cancer, *MTC* medullary thyroid cancer, *NGS* next-generation sequencing, *NSCLC* non-small cell lung cancer, *PD-(L)1* programmed death-1 (PD-1)/programmed death ligand-1 (PD-L1), *PTC* papillary thyroid carcinoma, *RT-PCR* reverse transcription-polymerase chain reaction

^a^ECOG PS scores range from 0 to 5, with higher scores indicating greater disability

^b^Fusion status was assayed by multiple techniques in some patients

^c^Other test methods included amplification refractory mutation system-polymerase chain reaction (ARMS-PCR), fluorescence in situ hybridization (FISH) and immunohistochemistry (IHC)

^d^Other fusion partners included *ANK3-RET*, *TRIM27-RET*, *PRKAR1A-RET*, *BMS1-RET*, *HSD3B1-RET*, and *RAB18-RET*

^e^*RET* fusion was indicated by molecular analysis, but specific partner was not identified

^f^Other mutations included C634R/W/Y, A883F, C816G, and C611R

^g^Other systemic therapies included Chinese medicine, radiotherapy and recombinant human endostatin

At the data cut-off date of November 30, 2023, the median follow-up time was 8.28 months (95% confidence interval [CI] 6.57–8.51). A total of 37 patients (30.3%, 37/122) had discontinued SY-5007 treatment due to disease progression or death (*n* = 29), withdrawal of consent (*n* = 6), or adverse events (AEs, *n* = 2). Meanwhile, 69.7% (85/122) of patients remained on treatment (Supplementary Fig. 2).

### Safety

At the data cut-off date, all patients had received as least one dose of SY-5007 and were evaluable for safety analysis. No dose-limiting toxicity (DLT) was observed, and the maximum tolerated dose (MTD) was not reached. A total of 96.7% (118/122) of patients experienced at least one treatment-related adverse event (TRAE). TRAEs with an incidence of ≥ 50% included increased aspartate aminotransferase (AST, 69.7%), increased alanine aminotransferase (ALT, 56.6%), diarrhea (54.1%), and neutropenia (54.1%), all of which were reversible with appropriate treatment discontinuation. Grade ≥ 3 TRAE occurred in 57.4% (70/122) of patients. The most common grade ≥ 3 TRAEs (incidence of ≥ 5%) were hypertension (22.1%), diarrhea (16.4%), hypertriglyceridemia (6.6%), and neutropenia (6.6%). The TRAEs in each dose group are summarized in Table 2.

**Table 2:** Most common TRAEs (≥ 15%) in patients received SY-5007 in phase 1 trial

	Total (*n* = 122)			20 mg QD (*n* = 1)		20 mg BID (*n* = 1)		40 mg BID (*n* = 3)		80 mg BID (*n* = 3)		120 mg BID (*n* = 3)		160 mg BID (*n* = 101)		200 mg BID (*n* = 10)	
TRAEs, *n* (%)	All Grade	Grade 1–2	Grade ⩾ 3	Grade 1–2	Grade ⩾ 3	Grade 1–2	Grade ⩾ 3	Grade 1–2	Grade ⩾ 3	Grade 1–2	Grade ⩾ 3	Grade 1–2	Grade ⩾ 3	Grade 1–2	Grade ⩾ 3	Grade 1–2	Grade ⩾ 3
**Any TRAE**	118 (96.7)	48 (39.3)	70 (57.4)	1 (100.0)	0 (0.0)	1 (100.0)	0 (0.0)	3 (100.0)	0 (0.0)	3 (100.0)	0 (0.0)	2 (66.7)	1 (33.3)	35 (34.7)	62 (61.4)	3 (30.0)	7 (70.0)
Increased aspartate aminotransferase	85 (69.7)	81 (66.4)	4 (3.3)	0 (0.0)	0 (0.0)	0 (0.0)	0 (0.0)	0 (0.0)	0 (0.0)	2 (66.7)	0 (0.0)	3 (100.0)	0 (0.0)	68 (67.3)	4 (4.0)	8 (80.0)	0 (0.0)
Increased alanine aminotransferase	69 (56.6)	63 (51.6)	6 (4.9)	0 (0.0)	0 (0.0)	1 (100.0)	0 (0.0)	0 (0.0)	0 (0.0)	1 (33.3)	0 (0.0)	2 (66.7)	1 (33.3)	52 (51.5)	5 (5.0)	7 (70.0)	0 (0.0)
Diarrhea	66 (54.1)	46 (37.7)	20 (16.4)	0 (0.0)	0 (0.0)	0 (0.0)	0 (0.0)	0 (0.0)	0 (0.0)	1 (33.3)	0 (0.0)	2 (66.7)	0 (0.0)	39 (38.6)	16 (15.8)	4 (40.0)	4 (40.0)
Neutropenia	66 (54.1)	58 (47.5)	8 (6.6)	1 (100.0)	0 (0.0)	1 (100.0)	0 (0.0)	0 (0.0)	0 (0.0)	2 (66.7)	0 (0.0)	2 (66.7)	0 (0.0)	48 (47.5)	7 (6.9)	4 (40.0)	1 (10.0)
Leukopenia	56 (45.9)	51 (41.8)	5 (4.1)	1 (100.0)	0 (0.0)	1 (100.0)	0 (0.0)	0 (0.0)	0 (0.0)	2 (66.7)	0 (0.0)	1 (33.3)	0 (0.0)	41 (40.6)	4 (4.0)	5 (50.0)	1 (10.0)
Hypertriglyceridemia	55 (45.1)	47 (38.5)	8 (6.6)	0 (0.0)	0 (0.0)	0 (0.0)	0 (0.0)	0 (0.0)	0 (0.0)	1 (33.3)	0 (0.0)	1 (33.3)	0 (0.0)	42 (41.6)	8 (7.9)	3 (30.0)	0 (0.0)
Increased blood creatine phosphokinase MB	54 (44.3)	53 (43.4)	1 (0.8)	0 (0.0)	0 (0.0)	0 (0.0)	0 (0.0)	0 (0.0)	0 (0.0)	2 (66.7)	0 (0.0)	2 (66.7)	0 (0.0)	44 (43.6)	1 (1.0)	5 (50.0)	0 (0.0)
Hyperbilirubinemia	51 (41.8)	50 (41.0)	1 (0.8)	0 (0.0)	0 (0.0)	0 (0.0)	0 (0.0)	0 (0.0)	0 (0.0)	2 (66.7)	0 (0.0)	2 (66.7)	0 (0.0)	41 (40.6)	1 (1.0)	5 (50.0)	0 (0.0)
Increased blood lactate dehydrogenase	51 (41.8)	51 (41.8)	0 (0.0)	1 (100.0)	0 (0.0)	0 (0.0)	0 (0.0)	2 (66.7)	0 (0.0)	1 (33.3)	0 (0.0)	1 (33.3)	0 (0.0)	40 (39.6)	0 (0.0)	6 (60.0)	0 (0.0)
Hypertension	50 (41.0)	23 (18.9)	27 (22.1)	0 (0.0)	0 (0.0)	1 (100.0)	0 (0.0)	1 (33.3)	0 (0.0)	1 (33.3)	0 (0.0)	0 (0.0)	0 (0.0)	19 (18.8)	22 (21.8)	1 (10.0)	5 (50.0)
Thrombocytopenia	50 (41.0)	46 (37.7)	4 (3.3)	0 (0.0)	0 (0.0)	0 (0.0)	0 (0.0)	0 (0.0)	0 (0.0)	2 (66.7)	0 (0.0)	1 (33.3)	0 (0.0)	40 (39.6)	3 (3.0)	3 (30.0)	1 (10.0)
Albuminuria	45 (36.9)	44 (36.1)	1 (0.8)	1 (100.0)	0 (0.0)	1 (100.0)	0 (0.0)	1 (33.3)	0 (0.0)	2 (66.7)	0 (0.0)	2 (66.7)	0 (0.0)	31 (30.7)	1 (1.0)	6 (60.0)	0 (0.0)
Increased blood creatine	45 (36.9)	45 (36.9)	0 (0.0)	0 (0.0)	0 (0.0)	0 (0.0)	0 (0.0)	2 (66.7)	0 (0.0)	1 (33.3)	0 (0.0)	0 (0.0)	0 (0.0)	37 (36.6)	0 (0.0)	5 (50.0)	0 (0.0)
Increased blood alkaline phosphatase	43 (35.2)	43 (35.2)	0 (0.0)	0 (0.0)	0 (0.0)	0 (0.0)	0 (0.0)	0 (0.0)	0 (0.0)	1 (33.3)	0 (0.0)	1 (33.3)	0 (0.0)	36 (35.6)	0 (0.0)	5 (50.0)	0 (0.0)
Hyperphosphatemia	42 (34.4)	42 (34.4)	0 (0.0)	0 (0.0)	0 (0.0)	0 (0.0)	0 (0.0)	0 (0.0)	0 (0.0)	0 (0.0)	0 (0.0)	2 (66.7)	0 (0.0)	37 (36.6)	0 (0.0)	3 (30.0)	0 (0.0)
Hyperuricemia	41 (33.6)	41 (33.6)	0 (0.0)	0 (0.0)	0 (0.0)	0 (0.0)	0 (0.0)	0 (0.0)	0 (0.0)	1 (33.3)	0 (0.0)	1 (33.3)	0 (0.0)	35 (34.7)	0 (0.0)	4 (40.0)	0 (0.0)
Dry mouth	40 (32.8)	40 (32.8)	0 (0.0)	0 (0.0)	0 (0.0)	0 (0.0)	0 (0.0)	0 (0.0)	0 (0.0)	0 (0.0)	0 (0.0)	0 (0.0)	0 (0.0)	39 (38.6)	0 (0.0)	1 (10.0)	0 (0.0)
Increased blood creatine phosphokinase	40 (32.8)	38 (31.1)	2 (1.6)	0 (0.0)	0 (0.0)	0 (0.0)	0 (0.0)	0 (0.0)	0 (0.0)	1 (33.3)	0 (0.0)	0 (0.0)	0 (0.0)	34 (33.7)	2 (2.0)	3 (30.0)	0 (0.0)
Hypothyroidism	38 (31.1)	38 (31.1)	0 (0.0)	0 (0.0)	0 (0.0)	0 (0.0)	0 (0.0)	0 (0.0)	0 (0.0)	0 (0.0)	0 (0.0)	0 (0.0)	0 (0.0)	35 (34.7)	0 (0.0)	3 (30.0)	0 (0.0)
Facial edema	35 (28.7)	35 (28.7)	0 (0.0)	0 (0.0)	0 (0.0)	0 (0.0)	0 (0.0)	0 (0.0)	0 (0.0)	0 (0.0)	0 (0.0)	0 (0.0)	0 (0.0)	33 (32.7)	0 (0.0)	2 (20.0)	0 (0.0)
Hypocalcemia	34 (27.9)	32 (26.2)	2 (1.6)	0 (0.0)	0 (0.0)	0 (0.0)	0 (0.0)	0 (0.0)	0 (0.0)	0 (0.0)	0 (0.0)	2 (66.7)	0 (0.0)	27 (26.7)	2 (2.0)	3 (30.0)	0 (0.0)
Hypoalbuminemia	33 (27.0)	33 (27.0)	0 (0.0)	0 (0.0)	0 (0.0)	0 (0.0)	0 (0.0)	0 (0.0)	0 (0.0)	1 (33.3)	0 (0.0)	1 (33.3)	0 (0.0)	27 (26.7)	0 (0.0)	4 (40.0)	0 (0.0)
Lymphopenia	30 (24.6)	27 (22.1)	3 (2.5)	0 (0.0)	0 (0.0)	0 (0.0)	0 (0.0)	1 (33.3)	0 (0.0)	0 (0.0)	0 (0.0)	1 (33.3)	0 (0.0)	24 (23.8)	3 (3.0)	1 (10.0)	0 (0.0)
Increased γ-Glutamyl transferase	28 (23.0)	27 (22.1)	1 (0.8)	0 (0.0)	0 (0.0)	0 (0.0)	0 (0.0)	1 (33.3)	0 (0.0)	2 (66.7)	0 (0.0)	3 (100.0)	0 (0.0)	18 (17.8)	1 (1.0)	3 (30.0)	0 (0.0)
Anemia	27 (22.1)	26 (21.3)	1 (0.8)	0 (0.0)	0 (0.0)	0 (0.0)	0 (0.0)	0 (0.0)	0 (0.0)	1 (33.3)	0 (0.0)	1 (33.3)	0 (0.0)	21 (20.8)	1 (1.0)	3 (30.0)	0 (0.0)
Hyponatremia	23 (18.9)	20 (16.4)	3 (2.5)	0 (0.0)	0 (0.0)	0 (0.0)	0 (0.0)	1 (33.3)	0 (0.0)	0 (0.0)	0 (0.0)	1 (33.3)	0 (0.0)	16 (15.8)	3 (3.0)	2 (20.0)	0 (0.0)
Conjugated Hyperbilirubinemia	23 (18.9)	23 (18.9)	0 (0.0)	0 (0.0)	0 (0.0)	0 (0.0)	0 (0.0)	0 (0.0)	0 (0.0)	0 (0.0)	0 (0.0)	1 (33.3)	0 (0.0)	19 (18.8)	0 (0.0)	3 (30.0)	0 (0.0)
Peripheral edema	23 (18.9)	23 (18.9)	0 (0.0)	0 (0.0)	0 (0.0)	0 (0.0)	0 (0.0)	0 (0.0)	0 (0.0)	1 (33.3)	0 (0.0)	0 (0.0)	0 (0.0)	20 (19.8)	0 (0.0)	2 (20.0)	0 (0.0)
Palmoplantar erythema syndrome	23 (18.9)	20 (16.4)	3 (2.5)	0 (0.0)	0 (0.0)	0 (0.0)	0 (0.0)	0 (0.0)	0 (0.0)	0 (0.0)	0 (0.0)	0 (0.0)	0 (0.0)	18 (17.8)	3 (3.0)	2 (20.0)	0 (0.0)
Hyperglycemia	22 (18.0)	22 (18.0)	0 (0.0)	0 (0.0)	0 (0.0)	0 (0.0)	0 (0.0)	1 (33.3)	0 (0.0)	0 (0.0)	0 (0.0)	1 (33.3)	0 (0.0)	16 (15.8)	0 (0.0)	4 (40.0)	0 (0.0)
Weight loss	22 (18.0)	21 (17.2)	1 (0.8)	0 (0.0)	0 (0.0)	0 (0.0)	0 (0.0)	0 (0.0)	0 (0.0)	0 (0.0)	0 (0.0)	2 (66.7)	0 (0.0)	16 (15.8)	1 (1.0)	3 (30.0)	0 (0.0)
Xeromycteria	21 (17.2)	21 (17.2)	0 (0.0)	0 (0.0)	0 (0.0)	0 (0.0)	0 (0.0)	0 (0.0)	0 (0.0)	0 (0.0)	0 (0.0)	1 (33.3)	0 (0.0)	18 (17.8)	0 (0.0)	2 (20.0)	0 (0.0)
Weakness	20 (16.4)	17 (13.9)	3 (2.5)	0 (0.0)	0 (0.0)	0 (0.0)	0 (0.0)	0 (0.0)	0 (0.0)	0 (0.0)	0 (0.0)	0 (0.0)	0 (0.0)	16 (15.8)	3 (3.0)	1 (10.0)	0 (0.0)
Hypopotassemia	19 (15.6)	14 (11.5)	5 (4.1)	0 (0.0)	0 (0.0)	0 (0.0)	0 (0.0)	1 (33.3)	0 (0.0)	1 (33.3)	0 (0.0)	0 (0.0)	0 (0.0)	11 (10.9)	5 (5.0)	1 (10.0)	0 (0.0)

*BID* bis in die, *QD* quaque die, *TRAE* treatment-related adverse event

TRAE-induced dose interruption and reduction were observed in 56 (45.9%) and 29 (23.8%) patients, respectively. The most common TRAEs (incidence of ≥ 5%) leading to dose interruption were hypertension (12.3%) and diarrhea (10.7%). Hypertension (5.7%) was the primary cause of dose reduction. Dose discontinuation occurred in 2 (1.6%) patients, due to one case of drug eruption and one instance of peripheral arterial thrombosis. Treatment-related serious adverse events (TRSAEs) occurred in 13 (10.7%) patients, with no treatment-related deaths reported.

### Pharmacokinetics (PK)

Forty-three evaluable patients (17 in dose-escalation phase, 26 in dose-expansion phase) were included for PK analysis. After a single-dose administration, the mean time to maximum concentration (*T*_max_) ranged from 0.5 h to 4.0 h across different dose cohorts, and mean elimination half-life (*T*_1/2_) ranged from 6.8 h to 44.4 h. SY-5007 exposure increased in a dose-proportional manner from 20 mg to 160 mg, with maximum concentration (*C*_max_) ranging from 214.0 to 3468.0 ng/mL and area under the concentration-time curve (AUC) from the time of dosing extrapolated to infinity, based on the last observed concentration (AUC_INF_obs_) ranging from 2407.0 to 21315.5 hr*ng/mL. Notably, SY-5007 exposure (*C*_max_ and AUC) did not exhibit a significant increase between 160 mg and 200 mg, suggesting saturation at these doses. After multiple-dose administration on Cycle 1 Day 28, SY-5007 exposure (*C*_max_ and AUC) increased significantly with repeated dosing in the 20 mg to 160 mg range. Modest drug accumulation was observed at steady state. (Table 3 and Supplementary Fig. 3).

**Table 3:** PK parameters of patients received SY-5007 in phase 1 trial

PK Parameter	AUC_INF_obs_ (hr^*^ng/mL)		AUC_0–12_ _h_ (hr^*^ng/mL)		*C*_max_ (ng/mL)		*T*_1/2_ (hr)		*T*_max_ (hr)		MRT_INF_obs_ (hr)	
**Cycle 1 Day 1**	Mean	SD	Mean	SD	Mean	SD	Mean	SD	Mean	SD	Mean	SD
20 mg QD (*n* = 1)	2407.0	N.D.	2226.3	N.D.	391.0	N.D.	6.8	N.D.	1.0	N.D.	7.7	N.D.
20 mg BID (*n* = 1)	3829.0	N.D.	1575.2	N.D.	214.0	N.D.	12.1	N.D.	4.0	N.D.	16.7	N.D.
40 mg BID (*n* = 3)	3764.0	751.2	2722.3	633.5	713.7	372.9	8.7	1.2	0.5	0.3	8.9	2.8
80 mg BID (*n* = 3)	7219.9	3622.9	5147.5	3591.2	1448.7	1363.1	37.8	26.9	0.5	0.9	12.4	6.0
120 mg BID (*n* = 3)	14057.1	6127.7	9271.2	4162.6	2473.3	1300.2	44.4	9.7	0.5	0.3	11.6	2.0
160 mg BID (*n* = 23)	21315.5	7146.8	16716.1	6842.1	3468.0	1413.6	10.8	18.8	1.0	2.3	8.2	3.3
200 mg BID (*n* = 9)	21589.9	6130.3	14852.6	7602.8	3182.2	1774.4	23.9	20.0	1.0	0.6	15.0	15.3
**Cycle 1 Day 28**	Mean	SD	Mean	SD	Mean	SD	Mean	SD	Mean	SD	Mean	SD
20 mg QD (*n* = 1)	2341.5	N.D.	2204.5	N.D.	388.0	N.D.	6.0	N.D.	1.0	N.D.	7.7	N.D.
20 mg BID (*n* = 1)	4017.9	N.D.	2939.8	N.D.	640.0	N.D.	7.3	N.D.	0.5	N.D.	9.0	N.D.
40 mg BID (*n* = 3)	7098.2	1857.9	5807.5	1481.0	1277.0	319.5	5.0	0.3	0.5	0.3	6.8	0.4
80 mg BID (*n* = 3)	18798.8	3011.7	12573.4	3876.6	1960.0	535.1	5.5	0.5	1.0	6.4	8.2	0.9
120 mg BID (*n* = 3)	34020.6	21465.0	21508.1	12110.8	2870.0	1236.5	7.0	2.8	1.0	0.0	10.5	4.2
160 mg BID (*n* = 15)	67151.1	41370.5	36825.6	16278.5	4734.7	1688.7	9.0	5.3	2.0	1.4	13.9	8.2
200 mg BID (*n* = 5)	63667.3	31686.5	41101.1	15836.1	5518.0	2137.8	7.0	2.3	2.0	2.0	10.8	3.3

*AUC*_*0-12 h*_ area under the plasma drug concentration versus time curve from time 0 to 12 h after drug administration, *AUC*_*INF_obs*_ area under the concentration-time curve from the time of dosing extrapolated to infinity, based on the last observed concentration, *C*_max_ maximum plasma drug concentration, *MRT*_*INF_obs*_ mean residence time from the time of dosing extrapolated to infinity, based on the last observed concentration, *N.D.* not determined, *PK* pharmacokinetics, *SD* standard deviation, *T*_1/2,_ elimination half-life, *T*_max_ time of maximum plasma drug concentration

### Efficacy

At the data cut-off date, efficacy assessments were conducted for 116 out of the 122 enrolled patients. Three patients had not yet undergone efficacy evaluation following the initial administration, and three withdrew the consent. Among the efficacy-evaluable patients, 114 had measurable lesions. One patient with gastric cancer, who had a non-measurable lesion, was classified as having progressive disease (PD) due to increased ascites after 2 cycles of SY-5007 treatment. Another patient with NSCLC and a non-measurable lesion was categorized as having stable disease (SD) after 1 cycle of SY-5007 treatment.

**Fig. 1:**
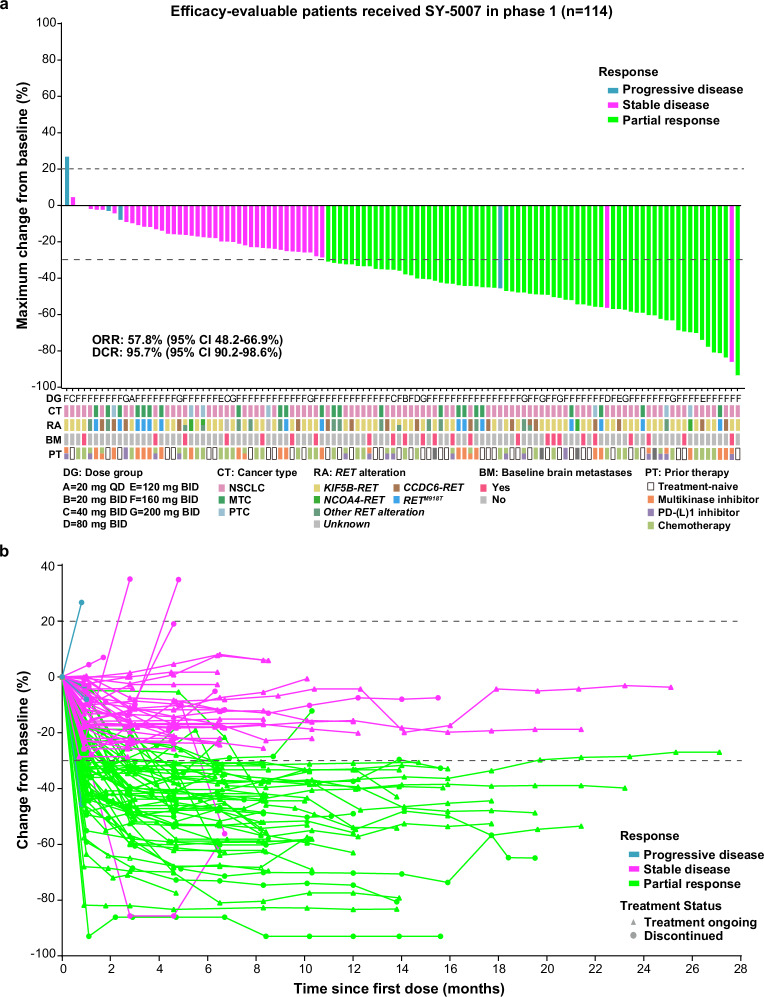
Tumor response in patients with *RET*-altered solid tumors receiving SY-5007. **a** Waterfall plots of the maximum tumor size change in 114 efficacy-evaluable patients with measurable lesions in *RET*-altered solid tumors. **b** The change in tumor burden over time in patients for whom postbaseline tumor data were available. BID bis in die, DCR disease control rate, MTC medullary thyroid cancer, NSCLC non-small cell lung cancer, ORR objective response rate, PTC papillary thyroid carcinoma, QD quaque die

**Fig. 2:**
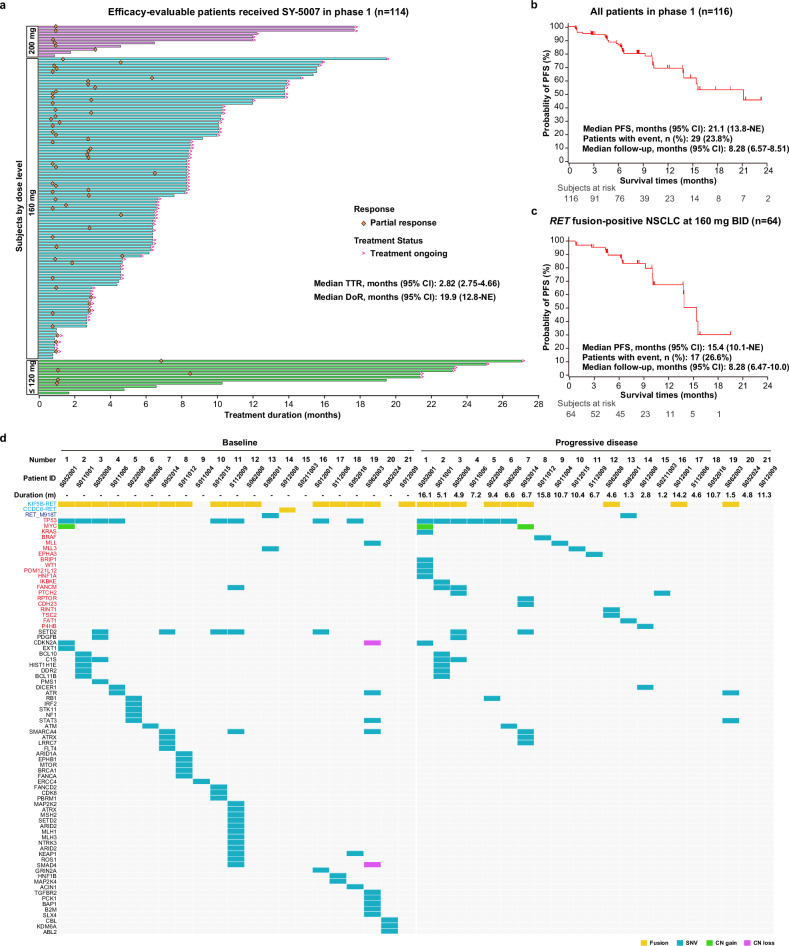
Efficacy of SY-5007 in patients with *RET*-altered solid tumors. **a** The time to response, the duration of treatment, and patient status by the data cut-off date for all enrolled patients with *RET*-altered solid tumors, according to the dose of SY-5007. **b**, **c** Kaplan-Meier curve of the progression-free survival in all patients (**b**) and NSCLC patients at 160 mg BID (**c**). Data shown were determined by investigator assessments. **d** Landscape of gene alterations in ctDNA from patients with progressive disease. The figure illustrates concurrent gene alterations identified in plasma samples from patients at baseline and after disease progression. Each column represents an individual patient for whom data are available. Within each column, colored rectangles indicate specific gene alterations, categorized by alteration type. Newly acquired genetic alterations in patients with progressive disease are highlighted in red font. CI confidence interval, CN copy number, NE not evaluated, NSCLC non-small cell lung cancer, PFS progression-free survival, SNV single nucleotide variation

Overall, target tumor shrinkage was observed in 95.7% of patients (111/116). The overall objective response rate (ORR) and disease control rate (DCR) were 57.8% (67/116, 95% CI 48.2-66.9%) and 95.7% (111/116, 95% CI 90.2–98.6%), respectively (Fig. 1a). SY-5007 induced rapid and durable responses (Figs. 1b, 2a), with a median time to first response (TTR) of 2.82 months (95% CI 2.75–4.66) and a median duration of response (DoR) of 19.9 months (95% CI 12.8-not evaluated [NE]). The median PFS was 21.1 months (95% CI 13.8-NE), with 29 (23.8%) patients experiencing progression events or death (Supplementary Table 3). The estimated 6-month and 12-month PFS rates were 86.1% (95% CI 77.6–91.6%) and 68.9% (95% CI 55.8–78.8%), respectively (Fig. 2b).

In the dose-escalation phase, the ORR and DCR were 52.9% (9/17, 95% CI 27.8–77.0%) and 94.1% (16/17, 95% CI 71.3–99.9%), respectively. In the dose-expansion phase, the ORR and DCR were 58.6% (58/99, 95% CI 48.2–68.4%) and 96.0% (95/99, 95% CI 90.0–98.9%), respectively. Specifically, for all patients receiving SY-5007 at 160 mg BID or 200 mg BID, the ORRs were 58.9% (56/95, 95% CI 48.4–68.9%) and 60.0% (6/10, 95% CI 26.2–87.8%), respectively, and DCRs were 94.7% (90/95, 95% CI 88.1–98.3%) and 100.0% (10/10, 95% CI 69.2–100.0%), respectively (Table 4).

**Table 4:** Efficacy of SY-5007 in patient with *RET*-altered solid tumors

	All Patients	20 mg QD	20 mg BID	40 mg BID	80 mg BID	120 mg BID	160 mg BID	200 mg BID
**All patients (*n*)**	116	1	1	3	3	3	95	10
Best Response, *n* (%)								
Partial response	67 (57.8)	0 (0.0)	1 (100.0)	1 (33.3)	1 (33.3)	2 (66.7)	56 (58.9)	6 (60.0)
Stable disease	44 (37.9)	1 (100.0)	0 (0.0)	2 (66.7)	2 (66.7)	1 (33.3)	34 (35.8)	4 (40.0)
Progressive disease	5 (4.3)	0 (0.0)	0 (0.0)	0 (0.0)	0 (0.0)	0 (0.0)	5 (5.3)	0 (0.0)
ORR, % (95% CI)	57.8 (48.2–66.9)	0 (0.0–97.5)	100.0 (2.5–100.0)	33.3 (0.8–90.6)	33.3 (0.8–90.6)	66.7 (9.4–99.2)	58.9 (48.4–68.9)	60.0 (26.2–87.8)
DCR, % (95% CI)	95.7 (90.2–98.6)	100.0 (2.5–100.0)	100.0 (2.5–100.0)	100.0 (29.2–100.0)	100.0 (29.2–100.0)	100.0 (29.2–100.0)	94.7 (88.1–98.3)	100.0 (69.2–100.0)
Median TTR, months (95% CI)	2.82 (2.75–4.66)	NE (NE-NE)	6.73 (NE-NE)	NE (1.14-NE)	NE (1.21-NE)	8.37 (1.18-NE)	2.79 (2.75–4.63)	1.14 (0.91-NE)
Median DoR, months (95% CI)	19.9 (12.8–NE)	NE (NE-NE)	NE (NE-NE)	9.16 (NE-NE)	NE (NE-NE)	19.9 (NE-NE)	13.0 (12.8-NE)	NE (5.06-NE)
Median PFS, months (95% CI)	21.1 (13.8–NE)	4.83 (NE-NE)	NE (NE-NE)	10.2 (1.67-NE)	NE (6.63-NE)	NE (21.1-NE)	15.4 (13.8-NE)	NE (5.75-NE)
Median follow-up, months (95% CI)	8.28 (6.57–8.51)	NE (NE-NE)	27.1 (NE-NE)	25.0 (NE-NE)	23.2 (23.2-NE)	21.4 (21.3-NE)	8.24 (6.44-8.34)	11.9 (0.88-NE)
**Treatment-naïve patients (n)**	40	0	0	1	0	0	37	2
Best Response, *n* (%)								
Partial response	28 (70.0)	0 (0.0)	0 (0.0)	0 (0.0)	0 (0.0)	0 (0.0)	26 (70.3)	2 (100.0)
Stable disease	10 (25.0)	0 (0.0)	0 (0.0)	1 (100.0)	0 (0.0)	0 (0.0)	9 (24.3)	0 (0.0)
Progressive disease	2 (5.0)	0 (0.0)	0 (0.0)	0 (0.0)	0 (0.0)	0 (0.0)	2 (5.4)	0 (0.0)
ORR, % (95% CI)	70.0 (53.5–83.4)	NE (NE-NE)	NE (NE-NE)	0 (0.0–97.5)	NE (NE-NE)	NE (NE-NE)	70.3 (53.0–84.1)	100.0 (15.8–100.0)
DCR, % (95% CI)	95.0 (83.1–99.4)	NE (NE-NE)	NE (NE-NE)	100.0 (2.5–100.0)	NE (NE-NE)	NE (NE-NE)	94.6 (81.8–99.3)	100.0 (15.8–100.0)
Median TTR, months (95% CI)	2.75 (0.95–2.89)	NE (NE-NE)	NE (NE-NE)	NE (NE-NE)	NE (NE-NE)	NE (NE-NE)	2.75 (0.98–2.89)	0.93 (0.91-NE)
Median DoR, months (95% CI)	14.5 (6.47–NE)	NE (NE-NE)	NE (NE-NE)	NE (NE-NE)	NE (NE-NE)	NE (NE-NE)	14.5 (9.26-NE)	5.32 (5.06-NE)
Median PFS, months (95% CI)	15.5 (9.23–NE)	NE (NE-NE)	NE (NE-NE)	1.67 (NE-NE)	NE (NE-NE)	NE (NE-NE)	15.5 (9.23-NE)	6.22 (5.98-NE)
Median follow-up, months (95% CI)	8.24 (6.27–8.37)	NE (NE-NE)	NE (NE-NE)	NE (NE-NE)	NE (NE-NE)	NE (NE-NE)	6.57 (6.27-8.37)	NE (NE-NE)
**Previously Treated patients (n)**	76	1	1	2	3	3	58	8
Best Response, *n* (%)								
Partial response	39 (51.3)	0 (0.0)	1 (100.0)	1 (50.0)	1 (33.3)	2 (66.7)	30 (51.7)	4 (50.0)
Stable disease	34 (44.7)	1 (100.0)	0 (0.0)	1 (50.0)	2 (66.7)	1 (33.3)	25 (43.1)	4 (50.0)
Progressive disease	3 (3.9)	0 (0.0)	0 (0.0)	0 (0.0)	0 (0.0)	0 (0.0)	3 (5.2)	0 (0.0)
ORR, % (95% CI)	51.3 (39.6–63.0)	0 (0.0–97.5)	100.0 (2.5–100.0)	50.0 (1.3–98.7)	33.3 (0.8–90.6)	66.7 (9.4–99.2)	51.7 (38.2–65.0)	50.0 (15.7–84.3)
DCR, % (95% CI)	96.1 (88.9–99.2)	100.0 (2.5–100.0)	100.0 (2.5–100.0)	100.0 (15.8–100.0)	100.0 (29.2–100.0)	100.0 (29.2–100.0)	94.8 (85.6–98.9)	100.0 (63.1–100.0)
Median TTR, months (95% CI)	4.63 (2.75-NE)	NE (NE-NE)	6.73 (NE-NE)	NE (1.14-NE)	NE (1.21-NE)	8.37 (1.18-NE)	4.56 (2.75-NE)	3.15 (0.91-NE)
Median DoR, months (95% CI)	19.9 (12.8-NE)	NE (NE-NE)	NE (NE-NE)	9.16 (NE-NE)	NE (NE-NE)	19.9 (NE-NE)	13.0 (9.23-NE)	NE (NE-NE)
Median PFS, months (95% CI)	NE (13.8-NE)	4.83 (NE-NE)	NE (NE-NE)	NE (10.2-NE)	NE (6.63-NE)	NE (21.1-NE)	15.4 (13.8-NE)	NE (5.75-NE)
Median follow-up, months (95% CI)	8.37 (6.60–11.9)	NE (NE-NE)	27.1 (NE-NE)	25.0 (NE-NE)	23.2 (23.2-NE)	21.4 (21.3-NE)	8.28 (6.44–8.41)	11.9 (0.88-NE)

*BID* bis in die, *CI* confidence interval, *DCR* disease control rate, *DoR* duration of response, *NE* not evaluated, *ORR* objective response rate, *PFS* progression-free survival, *QD* quaque die, *TTR* time to response

Among the 116 efficacy evaluable patients, 40 were treatment-naïve. For these patients, the ORR and DCR were 70.0% (28/40, 95% CI 53.5–83.4%) and 95.0% (38/40, 95% CI 83.1–99.4%), respectively. Of these, 37 received SY-5007 at 160 mg BID, with an ORR of 70.3% (26/37, 95% CI 53.0–84.1%) and a DCR of 94.6% (35/37, 95% CI 81.8–99.3%). Two patients received SY-5007 at 200 mg BID and both achieved a partial response (PR). Among 76 previously treated patients, the ORR was 51.3% (39/76, 95% CI 39.6–63.0%) and the DCR was 96.1% (73/76, 95% CI 88.9–99.2%). For these patients, 58 received SY-5007 at 160 mg BID, resulting in an ORR of 51.7% (30/58, 38.2–65.0%) and a DCR of 94.8% (55/58, 95% CI 85.6-98.9%). Eight patients received SY-5007 at 200 mg BID, with an ORR of 50.0% (4/8, 95% CI 15.7–84.3%) and DCR of 100.0% (8/8, 95% CI 63.1–100.0%, Table 4). Based on a thorough evaluation of safety, PK data, and the encouraging anti-tumor efficacy demonstrated in a substantial sample size, 160 mg BID of SY-5007 was selected as the RP2D.

Further analysis of SY-5007 at RP2D included 100 patients: 70 with *RET* fusion-positive NSCLC, 23 with *RET*-mutant MTC, and 7 with *RET* fusion-positive PTC. The most prevalent fusion partners in NSCLC were *KIF5B* (72.9%, 51/70) and *CCDC6* (17.1%, 12/70), while *NCOA4* (42.9%, 3/7) and *CCDC6* (28.6%, 2/7) were dominant in PTC. Among *RET*-mutant MTC patients, the most common mutation was *RET*^M918T^ mutation (65.2%, 15/23). Regarding treatment history, 45.7% (32/70) of NSCLC patients, 14.3% (1/7) of PTC patients, and 34.8% (8/23) of MTC patients were treatment-naïve (Supplementary Table 4).

Among 64 evaluable NSCLC patients, SY-5007 achieved an ORR of 64.1% (41/64, 95% CI 51.1–75.7%) and a DCR of 96.9% (62/64, 95% CI 89.2-99.6%, Supplementary Table 5). The median TTR was 2.75 months (95% CI 1.01–2.89), and the median DoR was 13.0 months (95% CI 9.26-NE). The median PFS was 15.4 months (95% CI 10.1-NE), and 26.6% of patients experienced progression or death. Estimated 6- and 12-month PFS rates were 89.6% (95% CI 78.2-95.2%) and 67.5% (95% CI 48.3–80.8%), respectively (Fig. 2c). Notably, SY-5007 demonstrated remarkable anti-tumor efficacy in both treatment-naïve and previously treated patients, with an ORR of 71.4% (20/28, 95% CI 51.3–86.8%) and 58.3% (21/36, 95% CI 40.8–74.5%), and a DCR of 100.0% (28/28, 95% CI 87.7–100.0%) and 94.4% (34/36, 95% CI 81.3-99.3%), respectively.

All 23 *RET*-mutant MTC patients and 7 *RET* fusion-positive PTC patients receiving SY-5007 at RP2D were evaluable for efficacy assessment, demonstrating an ORR of 52.2% (12/23, 95% CI 30.6–73.2%) and 42.9% (3/7, 95% CI 9.9–81.6%), and DCR of 91.3% (21/23, 95% CI 72.0–98.9%) and 100.0% (7/7, 95% CI 59.0–100.0%), respectively. Both the median DoR and PFS were not reached during the median follow-up of 6.44 months (95% CI 4.60-8.37) for MTC patients and 6.67 months (95% CI 2.79–8.37) for PTC patients and the estimated 12-month PFS rates were 85.2% (95% CI 60.5–95.0%) for MTC and 100.0% (95% CI 100.0–100.0%) for PTC. Additionally, SY-5007 exhibited anti-tumor efficacy in both treatment-naïve and previously treated patients, with an ORR of 75.0% (6/8, 95% CI 34.9–96.8%) and 40% (6/15, 95% CI 16.3–67.7%) for MTC patients, and 0.0% (0/1, 95% CI 0.0-97.5%) and 50.0% (3/6, 95% CI 11.8–88.2%) for PTC patients, respectively (Supplementary Table 5).

Moreover, SY-5007 exhibited notable intracranial anti-tumor efficacy in 3 evaluable patients with baseline measurable central nervous system (CNS) metastases. The intracranial ORR was 66.7% (2/3, 95% CI 9.4–99.2%) and DCR was 100.0% (3/3, 95% CI 29.2–100.0%) as assessed by the Response Assessment in Neuro-Oncology Brain Metastases (RANO-BM) criteria. Notably, in one case report involving a 63-year-old male NSCLC patient with *KIF5B-RET* fusion, a 10.8 mm intracranial lesion at baseline reduced by 40.7% after 4 weeks and by 56.5% after 12 weeks of treatment with SY-5007 at 160 mg BID (Supplementary Fig. 4), highlighting the promising intracranial efficacy of SY-5007. Ongoing assessments are underway to gain a more comprehensive understanding of its intracranial efficacy.

### Biomarker analysis

A retrospective analysis of circulating tumor DNA (ctDNA) profiling was performed using longitudinal plasma samples obtained at baseline, Cycle 1 Day 28, and post-progressive disease. At baseline, blood samples from all patients were subjected for NGS. Among them, 67 (54.9%) patients had detectable *RET* alterations in ctDNA, including *KIF5B-RET* (51/122, 41.8%), *CCDC6-RET* (5/122, 4.1%), *NCOA4-RET* (3/122, 2.5%), *RET*^M918T^ mutation (7/122, 5.7%) and other *RET* variation (1/122, 0.8%). 53 patients did not show detectable *RET* alterations in ctDNA, and 2 samples were ineligible for ctDNA profiling.

SY-5007 demonstrated significant anti-tumor efficacy in both patients with detectable and undetectable *RET* variations at baseline, achieving an ORR of 57.4% (35/61, 95% CI 44.1–70.0%) and 60.4% (32/53, 95% CI 46.0-73.5%), respectively, along with a median PFS of 13.8 months (95% CI 10.0–15.5) and NE (*p* = *0.0011*, Supplementary Fig. 5a and Supplementary Table 6). Specifically, within the group of patients with detectable *RET* alterations, the ORR was 58.7% (27/46, 95% CI 43.2–73.0%) for those with *KIF5B-RET*, 71.4% (5/7, 95% CI 29.0–96.3%) for those with other types of *RET* fusions, and 33.3% (3/9, 95% CI 7.5–70.1%) for those with *RET* mutations. Twenty-three patients with detectable *RET* alterations at baseline had ctDNA results available at Cycle 1 Day 28, including 20 with *KIF5B-RET* fusions, 2 with *NCOA4-RET* fusions, and 1 with a *CDCC6-RET* fusion. Notably, all 23 patients achieved a PR by radiologic tumor assessment. Longitudinal analysis revealed a rapid decline in the mean variant allele frequency (VAF) of *RET* alterations in all 23 patients at Cycle 1 Day 28. Remarkably, 21 patients achieved complete *RET* clearance (Supplementary Fig. 5b, c). Meanwhile, the ORR was 38.1% (16/42, 95% CI 23.6–54.4%) in patients with non-clearance or unknown *RET* alterations at Cycle 1 Day 28. The median TTR was 0.91 months (95% CI 0.88–0.95), which is shorter compared with the overall population (2.82 months [95% CI 2.75–4.66], Table 4).

*TP53* mutation has been identified as the most prevalent concomitant mutation in *RET*-rearranged NSCLC and is associated with a poor prognosis.^27^ Our analysis of SY-5007’s efficacy in patients with detectable *RET* variations at baseline, stratified by *TP53* mutation status, revealed that among those with a concomitant *TP53* mutation, SY-5007 demonstrated an ORR of 50.0% (11/22, 95% CI 28.2–71.8%) and a median PFS of 10.1 months (95% CI 6.21-NE). In contrast, patients without a concomitant *TP53* mutation showed an ORR of 61.5% (24/39, 95% CI 44.6–76.6%) and a median PFS of 15.4 months (95% CI 9.23-NE, Supplementary Table 7), indicating numerically inferior efficacy in patients with concurrent *TP53* mutation.

To elucidate the potential mechanisms of resistance to SY-5007, we conducted comprehensive ctDNA profiling. The molecular landscape of concurrent gene alterations, both at baseline and after PD, was assessed in 21 patients, with a median treatment duration of 6.7 months (range 1.3-16.1). Among the patients, 57.1% (12/21) exhibited at least one novel gene alteration after PD, including mutations in *TP53* (9.5%, 2/21), *FANCM* (9.5%, 2/21), *PTCH2* (9.5%, 2/21), *KRAS* (4.8%, 1/21), *BRAF* (4.8%, 1/21), *MLL* (4.8%, 1/21), *MLL3* (4.8%, 1/21) and *EPHA3* (4.8%, 1/21), as well as copy number gain in *MYC* (4.8%, 1/21). Notably, no treatment-induced novel on-target *RET* alterations were identified (Fig. 2d), possibly attributed to the relatively short treatment duration, highlighting the potential significance of off-target induced resistance to SY-5007 during this period.

## Discussion

In this first-in-human phase 1 study, SY-5007, a highly selective and potent RET inhibitor, demonstrated a manageable safety profile and promising anti-tumor efficacy in 122 patients with advanced solid tumors harboring activating *RET* alterations. Based on the evaluation of safety, PK properties, and efficacy, the RP2D of SY-5007 was determined at 160 mg BID.

SY-5007 exhibited a generally favorable tolerability profile with manageable AEs. Among patients treated with SY-5007, 57.4% experienced grade ≥ 3 TRAEs, with the most commonly observed being hypertension (22.1%), diarrhea (16.4%), hypertriglyceridemia (6.6%), and neutropenia (6.6%). TRSAEs occurred in 10.7% of patients. Compared to selpercatinib and pralsetinib, SY-5007 showed a unique safety profile with a comparable incidence of AEs. In the LIBRETTO-001 study,^28^ selpercatinib was associated with a 38.6% incidence of grade ≥ 3 TRAEs, with the most common being hypertension (13.2%), increased ALT (9.0%), and increased AST (6.3%). TRSAEs was observed in 11% of patients. In the ARROW study,^22,29^ pralsetinib was associated with a 54% incidence of grade ≥ 3 TRAEs, with the most common being neutropenia (20%), anemia (12%), hypertension (12%), lymphopenia (9%), and leukopenia (8%). TRSAEs occurred in 24% of patients. Regarding drug tolerability, SY-5007 exhibited lower rates of treatment-induced dose reductions, discontinuations, and deaths (23.8%, 1.6%, and 0%), compared to selpercatinib (41%, 3%, and 0.1%)^28^ and pralsetinib (38%, 7%, and 0.4%).^22,29^ While acknowledging the inherent limitations of indirect trial comparisons, this study highlighted a favorable safety profile of SY-5007. However, it is crucial to continue monitoring the safety profile over extended periods and to conduct comprehensive AE assessments in patients at RP2D during the phase 2 study.

Encouragingly, SY-5007 demonstrated promising clinical activity, with significant anti-tumor efficacy observed in both treatment-naïve and previously treated patients across various *RET*-altered tumor types, including NSCLC, PTC, and MTC, irrespective of fusion partners and prior MKI exposure. In *RET* fusion-positive NSCLC, SY-5007 achieved an ORR of 71.4% in treatment-naïve patients and 58.3% in previously treated patients at RP2D, which were comparable to those reported for pralsetinib and selpercatinib. The ARROW study of pralsetinib in *RET* fusion-positive NSCLC showed an ORR of 72% in treatment-naïve patients and 59% in previously treated patients.^29^ Similarly, selpercatinib reported an ORR of 84% in treatment-naïve patients in the LIBRETTO-431 study^30^ and 61% in previously treated patients in the LIBRETTO-001 study.^28^

With a median follow-up of 8.28 months, SY-5007 demonstrated a median DoR of 19.9 months and median PFS of 21.1 months, with 70.5% of patients still under treatment. The 1-year DoR and PFS rates were 74.1% and 68.9%, respectively, comparable to selpercatinib’s 1-year DoR and PFS rates of 66.1% and 70.6% for treatment-naïve patients, and 73.1% and 70.5% for previously treated patients.^28^ Overall, SY-5007 exhibits sustained clinical benefits and effective long-term disease control in patients with *RET*-fusion positive NSCLC, although further extended follow-up is warranted to confirm these findings.

Patient enrollment for *RET*-altered MTC and PTC was limited in this phase 1 study, reflecting their lower incidence. Despite this, SY-5007 showed significant efficacy at RP2D, with an ORR of 52.2% for *RET*-mutant MTC and 42.9% for *RET*-fusion positive PTC. These results warrant further investigation in larger populations. In cross-trial comparisons, SY-5007 demonstrated a remarkable ORR of 75.0% for treatment-naïve *RET*-mutant MTC, comparable to pralsetinib and selpercatinib. The ARROW study reported an ORR of 71% for pralsetinib,^31^ while the LIBRETTO-531 study showed an ORR of 69.4% for selpercatinib.^32^ These data suggest the promising potential of SY-5007 in treating *RET*-mutant MTC, meriting further exploration and validation in larger patient cohorts.

Given its favorable safety profile and potent anti-tumor activity, SY-5007 is a promising candidate for combination therapies in *RET*-altered solid tumors. Its unique therapeutic properties make it especially suitable for integration with chemotherapy and radiotherapy, both in adjuvant and consolidation settings. These integrated approaches hold the potential to eradicate residual cancer cells and extend disease (progression)-free survival, particularly for patients with early-stage or locally advanced *RET*-altered solid tumors, drawing valuable insights from the management of *EGFR/ALK*-altered NSCLC.^33–35^ Moreover, SY-5007’s potential in neoadjuvant therapy also merits exploration. Its ability to induce rapid tumor responses could potentially facilitate tumor downstaging and improve surgical resection outcomes.

ctDNA has emerged as a promising biomarker for monitoring treatment response, tracking tumor evolution, and uncovering resistance mechanisms.^36,37^ In biomarker analysis, SY-5007 demonstrated an impressive ORR of 57.4% in 61 patients with detectable *RET* alterations, aligning with the radiologic responses across the study populations. These findings support further investigation of SY-5007 in patients with ctDNA-detected *RET* alterations, particularly those lacking sufficient tissue for genomic profiling. Additionally, rapid ctDNA clearance of *RET* alteration correlated with better and quicker clinical responses, evidenced by an ORR of 100% and a median TTR of 0.91 months. This discovery further validates SY-5007’s on-target anti-tumor efficacy. However, the presence of concurrent *TP53* mutations in some patients was associated with numerically inferior outcomes, underscoring the necessity for additional validation studies.

Intriguingly, longitudinal ctDNA profiling of patients who relapsed after SY-5007 treatment revealed no on-target *RET* resistance mutation, such as the solvent-front *RET*^G810R/S/C/V^ mutations observed with selpercatinib^38^ or *RET*^G810C/S^ mutations associated with pralsetinib.^39^ Instead, off-target resistance mechanisms were observed in 57.1% of patients (12/21). These included activation of bypass pathways through alternative receptor tyrosine kinases (RTKs) that engage downstream RAS-MAPK signaling pathways, as well as dysregulation of DNA repair and cell cycle processes. The RETgistry study corroborates these findings, showing that off-target mechanisms, like bypass pathway activation, are the primary cause of drug resistance to selpercatinib and pralsetinib, accounting for 42% of cases, compared to only 14% due to on-target resistance mechanisms.^40^ These insights underscore the unique therapeutic profile of SY-5007 and suggest that combining it with agents that target pan-RAS, BRAF, and cell cycle pathways may be more effective in overcoming resistance than relying on next-generation RET inhibitors alone. Further investigation into these off-target resistance mechanisms is essential. This includes additional ctDNA and tissue sample analyses, as well as extended follow-up studies, to fully elucidate the resistance mechanisms and refine treatment strategies.

We recognize several limitations in this first-in-human study of SY-5007. Firstly, the phase 1 trial was a single-arm, open-label study with a selectively chosen patient population, which means that the preliminary safety and efficacy data must be interpreted with caution and require validation in larger, more diverse populations. Secondly, the median follow-up time of 8.28 months, while yielding promising median DoR and PFS, is relatively short. Although the majority of patients remained progression-free, with only 23.8% experiencing progression or death, the short follow-up period necessitates longer-term studies to fully evaluate the durability of SY-5007’s efficacy and safety. Thirdly, the small number of patients evaluable for brain metastases indicates a need for further investigation into SY-5007’s intracranial efficacy. Expanding the study to include more patients with brain metastases will provide a comprehensive understanding of the drug’s potential in treating this challenging aspect of cancer care.

In conclusion, the current phase 1 study highlights the promising clinical efficacy of SY-5007 in both treatment-naïve and prior treated patients with advanced *RET* fusion-positive NSCLC, *RET* fusion-positive PTC and *RET*-mutant MTC. Based on the encouraging anti-tumor efficacy and favorable safety profiles, a pivotal phase 2 study has been initiated to further assess the efficacy and safety of SY-5007 at RP2D (160 mg BID) in patients with locally advanced or metastatic *RET* fusion-positive NSCLC (NCT05278364).

## Methods

### Study design and participants

This single-arm, open-label, first-in-human, dose-escalation and dose-expansion phase 1 study was conducted across six sites in China (NCT05278364). SY-5007 was orally administered in a continuous 28-day cycle until disease progression, death, unacceptable toxicity, or withdrawal of informed consent. Dose escalation followed a 3 + 3 design, incorporating accelerated titration^41^ for the first two dose levels and a modified Fibonacci dose escalation.^42^ Patients received SY-5007 at a starting dose of 20 mg once daily (QD), with subsequent doses ranging from 20 to 200 mg BID. The phase 1 dose-escalation phase determined the MTD and RP2D of SY-5007. All patients enrolled in the dose-expansion phase received the recommended dose of 160 mg BID or 200 mg BID.

Eligible patients were aged ≥ 18 years old, with an Eastern Cooperative Oncology Group (ECOG) performance status (PS) score of 0 to 1. Patients were required to have advanced or metastatic solid tumors, including *RET* fusion-positive NSCLC, *RET*-mutant MTC, or other *RET*-altered solid tumors. *RET* alterations were determined in tumor or ctDNA in blood by NGS, FISH, RT-PCR or IHC per local testing. In the dose-escalation phase, eligibility was limited to patients who had previously received standard-of-care treatments (immune checkpoint inhibitors, MKIs, chemotherapy, or radiotherapy) or those ineligible for standard/available therapies. In the dose-expansion phase, treatment-naïve patients were allowed. Additional eligibility criteria included having at least one measurable lesion per Response Evaluation Criteria in Solid Tumors version 1.1 (RECIST 1.1).^43^

This study was conducted in accordance with the ethical principles of Good Clinical Practice and the Declaration of Helsinki, and was based on the International Council for Harmonisation E6 requirements. The full protocol was approved by the institutional review board or independent ethics committee of each participating site, and all patients provided signed informed consent (Ethics Committee Approval Number: 21256ZL for Shanghai Pulmonary Hospital).

### Study assessments

Radiologic tumor assessments were conducted by computed tomography (CT) or magnetic resonance imaging (MRI) of brain, chest, abdomen, and pelvis at baseline, 4 weeks for the first tumor assessment, then every 8 weeks (± 7 days) for 1 year, and every 12 weeks (± 7 days) thereafter. MTC patients were longitudinally monitored for serum calcitonin and carcinoembryonic antigen (CEA) levels.

PK evaluation was performed for all patients who received at least one dose of SY-5007. For single-dose PK evaluation, serial plasma samples were collected at prespecified time points in Cycle 1, including pre-dose and various time points after a single dose of SY-5007. Serial plasma samples were also collected in each patient after the first dose and during multiple-dose administration on Cycle 1 Day 28. PK parameters included *C*_max_, *T*_max_, *T*_1/2_, AUC_INF_obs_, area under the plasma drug concentration versus time curve from time 0 to 12 h after drug administration (AUC_0-12 h_), and mean residence time from the time of dosing extrapolated to infinity, based on the last observed concentration (MRT_INF_obs_).

AEs were assessed from the initiation of treatment until 28 days after the last dose of SY-5007 and graded according to the National Cancer Institute Common Terminology Criteria for Adverse Events, version 5.0.

### Biomarker analysis

For molecular profiling, plasma samples were collected at baseline, Cycle 1 Day 28, and post-progressive disease, and the ctDNA were isolated for analysis. Gene alteration status was analyzed at a central laboratory using a validated, commercially available 1021-gene NGS panel (GenePlus, Beijing, China).

### Study endpoints

The primary endpoints for this study were to determine the MTD, DLT, RP2D and safety. Secondary endpoints included PK parameters and preliminary anti-tumor activity of SY-5007, such as ORR (defined as the proportion of patients who had complete response [CR] or PR), DCR (defined as the proportion of patients who had CR, PR or SD) as assessed by the investigators and independent review committee (IRC) according to the RECIST 1.1. All responses required a confirmation of radiologic assessment at least 4 weeks after the first assessment.

### Statistical analysis

For data analysis, patients initially enrolled into the study were included in the intent-to-treat (ITT) population (*n* = 122). The Safety Analysis Set (SAS) comprised all enrolled patients who received at least one dose of SY-5007 and had post-treatment safety records (*n* = 122). The Per-Protocol Set (PPS) included all patients who received at least one dose of SY-5007 and underwent at least one follow-up tumor assessment (*n* = 116). The Pharmacokinetic Analysis Set (PKAS) included all patients with at least one evaluable PK sample (*n* = 43). Descriptive statistics were used for baseline data, summarizing continuous data with mean, standard deviation, median, minimum, and maximum, and categorical data with frequency and percentage. Safety analysis was descriptive, including adverse events summarized by frequency and severity. PK parameter analysis was performed using Phoenix WinNonlin version 8.0 (Certara L.P. [Pharsight], St. Louis, MO, USA), and results were summarized using geometric mean and coefficient of variation. CIs for response rates were calculated using the Clopper-Pearson method. Time-to-event data (DoR, PFS and OS) and follow-up times were estimated using the Kaplan-Meier method, with median times and 95% CIs provided. All analysis were performed with SAS statistical software, version 9.2 (SAS Institute).

## Supplementary information

Figures. S1 to S5, Tables S1 to S7

Study protocol

## Data Availability

The raw sequencing data reported in this paper have been deposited in the Genome Sequence Archive in National Genomics Data Center, China National Center for Bioinformation/Beijing Institute of Genomics, Chinese Academy of Sciences (https://ngdc.cncb.ac.cn/gsa-human). These data are accessible under the accession number: HRA008677. These data are under controlled access by human privacy regulations and are only available for research purposes. Access to the data can be granted following approval from the Data Access Committee of the GSA-human database. Shouyao Holdings (Beijing) Co., Ltd, Beijing, China, is the owner of SY-5007 and is dedicated to ensuring data transparency. Other datasets used and/or analyzed during the current study are available from the corresponding author upon reasonable request.
