# Supplementary material for: First-in-human, phase 1 dose-escalation and dose-expansion study of a RET inhibitor SY-5007 in patients with advanced RET-altered solid tumors
Source: Signal Transduct Target Ther. 2024 Nov 4;9:300. doi: 10.1038/s41392-024-02006-9 (PMC11532403; doi:10.1038/s41392-024-02006-9)
Supplement: Supplementary file 1 — Figures. S1 to S5, Tables S1 to S7 [file 41392_2024_2006_MOESM1_ESM.docx]

Supplementary Materials for

First-in-human, phase 1 dose-escalation and dose-expansion study of SY-5007 in patients with advanced RET-altered solid tumors

Wei Li^†^, Yongsheng Wang^†^, Anwen Xiong^†^, Ge Gao, Zhengbo Song, Yiping Zhang, Dingzhi Huang, Feng Ye, Qiming Wang, Zhihui Li, Jiaye Liu, Chunwei Xu, Yinghui Sun, Xijie Liu, Fei Zhou and Caicun Zhou

^†^These authors contributed equally to this work.

Correspondence to: Caicun Zhou (caicunzhoudr@163.com) or Fei Zhou (fei.zhou@tongji.edu.cn)

**This PDF file includes:**

Materials and Methods

Figures. S1 to S5

Tables S1 to S7

**Other Supplementary Materials for this manuscript include the following:**

Study protocol

Materials and Methods

GST-RET expression and purification

RET (residues 658-1114) was cloned into pFastBac vector containing an N-terminal Glutathione S-transferase (GST) tag. Mutagenesis was performed on this vector to generate the RET^V804M^ single mutant: RETV804MmutF: CTCCTCATCaTGGAGTACGCC and RETV804MmutR: GGCGTACTCCAtGATGAGGAG; the RET^M918T^ single mutant: RETM918TmutF: GTTAAATGGAcGGCAATTGAA and RETM918TmutR: TTCAATTGCCgTCCATTTAAC; the RET^G810S^ single mutant: RETG810SmutF: GGAGTACGCCAAATACtcCTCCCTGCGGGGCTT and RETG810SmutR: AAGCCCCGCAGGGAGgaGTATTTGGCGTACTCC. Recombinant baculovirus for expression of GST-RET was generated by co-transfection of Sf9 cells. Plaque-purified viruses were amplified, titrated and used to infect Sf9 cells incubated at 27 ^o^C and 80 rpm. Cells were harvested 72 h post infection for protein purification.

Frozen cell pellets (1 liter culture/each) were thawed and resuspended in 20 ml of buffer A (50mM Tris-HCl, pH 7.5, 150mM NaCl, 0.1mM EDTA, 0.5% Triton X-100, 20% glycerol and 1* protease inhibitor [#539134-1SETCN, Merck]) and sonicated. The cell lysates were centrifuged at 20000 rpm for 40 min at 4 ^o^C, then applied to GSH-Sepharose 4 Fast Flow (GE Healthcare) at 4 ^o^C. After washing twice with 10 mL of buffer B (50mM Tris-HCl, pH 7.5, 150mM NaCl, 0.1 mM EDTA, 20% glycerol), the protein was eluted with elution buffer (50mM Tris-HCl, pH 7.5, 150mM NaCl, 0.1mM EDTA, 20% glycerol, 20mM Glutathione, 1 mM DTT) and concentrated using Amicon Ultra-15. Purities of proteins were 90% as determined by SDS-PAGE and Coomassie Blue stain.

*In vitro* biochemical assays

Kinase activity was detected using CisBio HTRF kinEASE-TK kit. The assay was performed in 384-well white plates (#3574, Corning) in a reaction volume of 10 μL containing a final concentration of 50 mM HEPES, pH 7.5, 0.1 mM Na3VO4,0.01% BSA, 0.001% Tween-20, 5 mM MgCl2, 5 mM MgCl2, 1 mM DTT, 1 μM TK-Substrate-biotin. The reaction was initiated by the addition of ATP at Km and indicated recombinant RET proteins. For RET^WT^, this was 20 μM and 0.25 nM; for RET^V804M^, this was 1.5 μM and 0.01 nM; for RET^M918T^, this was 1.5 μM and 0.01 nM, for RET^G810S^, this was 20 μM and 0.5 nM, respectively. The IC_50_ assays were performed using a 8-dose IC_50_ assay with a 5-fold serial dilution starting at 2 μmol/L. The assay was allowed to incubate at 23 ^o^C for 1 hour before terminating with the addition of 10 μL HTRF detection buffer containing EDTA supplemented with TK-antibody labelled with Eu^3+^-Cryptate (1:100 dilution) and streptavidin-XL665 (125 nM). After incubation at 23 ^o^C for 1 hour, FRET signal was measured on an EnVision reader (PerkinElmer Inc.).

Cell lines and cell culture

The cell lines HEK293T, Baf3, TT cell and NIH-3T3 were obtained from the Cell Resource Center, Peking Union Medical College (PCRC). HEK293T and NIH-3T3 were maintained in DMEM high glucose medium (#C3113-0500, Vivacell) supplemented with 10 % FBS (#10500064, Gibco) and 1 % Penicillin-Streptomycin (#15140122, Gibco). The murine Pro‐B cell line Baf3 was cultured in RPMI 1640 medium (#C3010, Vivacell) supplemented with 10 % FBS, 1 % Penicillin-Streptomycin and 1 ng/mL recombinant murine interleukin-3 (#213-13, PeproTech). All stably transformed BaF3 cells were cultured in RPMI 1640 without IL‐3. The TT MTC cell was grown in Ham's F-12K medium (#21127022, Gibco) supplemented with 10 % FBS and 1 % Penicillin-Streptomycin. Cells were cultured at 37 ^o^C under 5 % CO_2_ and high humidity conditions.

Generation of RET fusion or mutant-driven cell lines

KIF5B-RET, CCDC6-RET and mutations were cloned into pCDHL vector and were verified by sequencing. Reah RET expression vectors were separately co‐transfected with psPAX2 and pMD2.G packaging plasmids into 60-70% confluent HEK 293T cells maintained in 2 mL of culture medium in six‐well plates using Polyethyleneimine (PEI) transfection grade. Virus containing media was harvested after 48 h and filtered with 0.45 μm membrane and stored at -80 ^o^C prior to infection. For infection of Ba/F3 cells, 1 mL of viral supernatant and 8 μg/mL polybrene were added to 5 million cells per well in six‐well plates. Infected cells were selected with the addition of 1 μg/mL puromycin for 2 weeks in RPMI-1640/ 10% FBS/ 1 ng/mL IL-3 medium. Subsquently, puromycin-resistant cells were cultured in IL-3-free medium for 1 week and RET fusion proteins were confirmed by immunoblot analysis.

Proliferation assays

Baf3 cells were cultured in 96-well plates (1000 cells per well) and exposed to SY-5007 ranging from 10 μmol/L to 0.3 nmol/L for 72 hours at 37°C. Cell viability was assessed by luminescence using CellTiter-Glo 2.0 assay (Promega). IC_50_ curve fitting was performed using GraphPad Prism 9.0.

Tumor xenograft models

All procedures relating to animal handling, care, and treatment were performed according to the Beijing Laboratory Animal Management Regulations. 6-week-old male BALB/c nude mice were inoculated subcutaneously into the flank with Baf3-CCDC6-RET^WT^ (5*10^6^ cells/ 0.1 mL/ each), Baf3-CCDC6-RET^M918T^ (5*10^6^ cells/0.1 mL/each), Baf3-CCDC6-RET^V804M^ (5*10^6^ cells/ 0.1 mL/ each), or HEK293T-KIF5B-RET^V804M^ (5*10^6^ cells/ 0.1 mL/ each). Mice were randomized by tumor size into groups of 10 to 12 when tumor volume reached approximately 100-150 mm^3^. SY-5007 was suspended in 0.5% methyl cellulose (MC). For all experiments, mice were dosed twice daily with vehicle or SY-5007 for 9-14 days. Tumor size and body weight were measured two times each week, and tumor volume was calculated with the following formula: [length × (width)^2^]/ 2. Statistical significance was calculated by using two-way ANOVA test to assess the difference in tumor volumes between the control (vehicle-treated) and treatment groups.

Inclusion and exclusion criteria

To be eligible for this study, subjects must meet all of the following inclusion criteria:

1) Age ≥ 18 years old, male or female;

2) Eastern Cooperative Oncology Group (ECOG) performance status score of 0-1;

3) Estimated life expectancy > 12 weeks;

4) Subjects must have at least one assessable lesion in dose-escalation phase and one measurable lesion in dose-expansion phase according to RECIST v1.1;

5) Dose-escalation phase: subjects with histologically or cytologically confirmed advanced solid tumors who have failed standard therapy, or for whom no standard treatment is available, or for whom standard treatment is not available at this stage (e.g., subject refuses standard therapy), and for whom there is a RET gene fusion or mutation;

Dose-expansion phase: subjects with histologically or cytologically confirmed advanced solid tumors who have failed standard therapy, or for whom no standard treatment is available, or for whom standard treatment is not appropriate at this stage, and who have RET gene fusion in NSCLC or RET mutation in MTC or RET-altered other advanced solid tumors;

6) Subjects must have adequate organ function, defined as follows:

Liver function:

• Without liver metastases, serum aspartate aminotransferase (AST) and serum alanine aminotransferase (ALT) ≤ 3 times the upper limit of normal (ULN); subjects with liver metastases or hepatocellular carcinoma (HCC), AST, ALT ≤ 5 times ULN, total serum bilirubin (TBIL) ≤ 1.5 times ULN.

Bone marrow function (no transfusions or hematopoietic stimulating factor therapy within 10 days prior to testing):

• Neutrophil absolute value (ANC) ≥ 1.5×109/L;

• Platelets (PLT) ≥ 75×109/L;

• Hemoglobin (Hb) ≥ 85 g/L.

Kidney function:

• Creatinine clearance ≥ 50 mL/min.

Coagulation function:

• Prothrombin time (PT) or international normalized ratio (INR) ≤ 1.5×ULN.

Lipids:

• Cholesterol ≤ 500 mg/dL (12.92 mmol/L).

7) All women of childbearing age must have a negative serum pregnancy test within 7 days prior to the first dose, and male and female subjects of childbearing potential must agree to abstinence or use contraception throughout the study period and for at least 3 months after the last dose of drug;

8) Willingness and ability to give informed consent and follow protocol procedures, and comply with follow-up visit requirements.

Subjects with any of the following are not eligible for entry into this study:

1) Dose-expansion phase: subjects carry known major driver gene alterations other than *RET* genes, e.g., *EGFR*, *ALK*, *ROS1*, *KRAS*, etc.;

2) Dose-expansion phase: previous use of selective RET inhibitors;

3) Received antitumor therapy such as chemotherapy, radiotherapy, biological therapy, endocrine therapy, immunotherapy and other antitumor therapy within 4 weeks prior to first dose, except for the following:

• Nitrosourea or mitomycin C within 6 weeks prior to first dose;

• Oral fluorouracil analogs and small molecule-targeted drugs within 2 weeks prior to first dose or within 5 half-lives of the drug, whichever is longer;

• Traditional Chinese medicines with anti-tumor indications within 2 weeks prior to the first dose.

4) Received other unlisted clinical trial drugs or treatments within 4 weeks prior to the first dose;

5) Major organ surgery (excluding puncture biopsy) or had significant trauma within 4 weeks prior to the first dose, or required elective surgery during the trial;

6) Adverse effects of previous antitumor therapy have not returned to a CTCAE v5.0 grade ≤ 1 (except for toxicities judged by investigator to pose no safety risk, such as alopecia, grade 2 peripheral neurotoxicity, etc.);

7) Central nervous system (CNS) metastases with clinical symptoms, or other evidence of uncontrolled CNS metastases or meningeal metastases in subjects who, in the judgement of the investigator, are not suitable for enrollment;

8) Participants with active uncontrolled systemic bacterial, viral or fungal infection despite optimal treatment (not required to screen for chronic disease);

9) Active hepatitis B (HBV-DNA ≥ 2000 IU/mL), hepatitis C virus infection (HCV antibody positive), HIV antibody positive, active syphilis, and still uncontrolled after active treatment, and is judged by the investigator to be unsuitable for enrollment;

10) History of severe cardiovascular and cerebrovascular disease, including but not limited to:

• Severe cardiac rhythm or conduction abnormalities, such as ventricular arrhythmias requiring clinical intervention, degree II-III atrioventricular block, etc;

• At rest, mean QT interval corrected using Fridericia’s formula (QTcF) > 480 ms;

• Acute coronary syndrome, congestive heart failure, aortic dissection, stroke, or other grade 3 or above cardiovascular and cerebrovascular events within 6 months prior to the first dose;

• New York Heart Association (NYHA) ≥ class II heart failure or left ventricular ejection fraction (LVEF) < 50%;

•High blood pressure remains uncontrolled despite aggressive antihypertensive therapy. Uncontrolled hypertension is defined as systolic blood pressure > 185 mmHg and/or diastolic blood pressure > 110 mmHg measured three times at least 10 minutes apart;

11) Used any CYP3A4 inhibitor or inducer within 14 days prior to the first dose;

12) Inability to swallow the drug orally, or conditions that, in the judgment of the investigator, severely interfere with gastrointestinal absorption;

13) Subjects who, judged by the investigator, have a history of other serious systemic diseases, or are otherwise reasons unsuitable for participation in this clinical study.

Figure. S1.


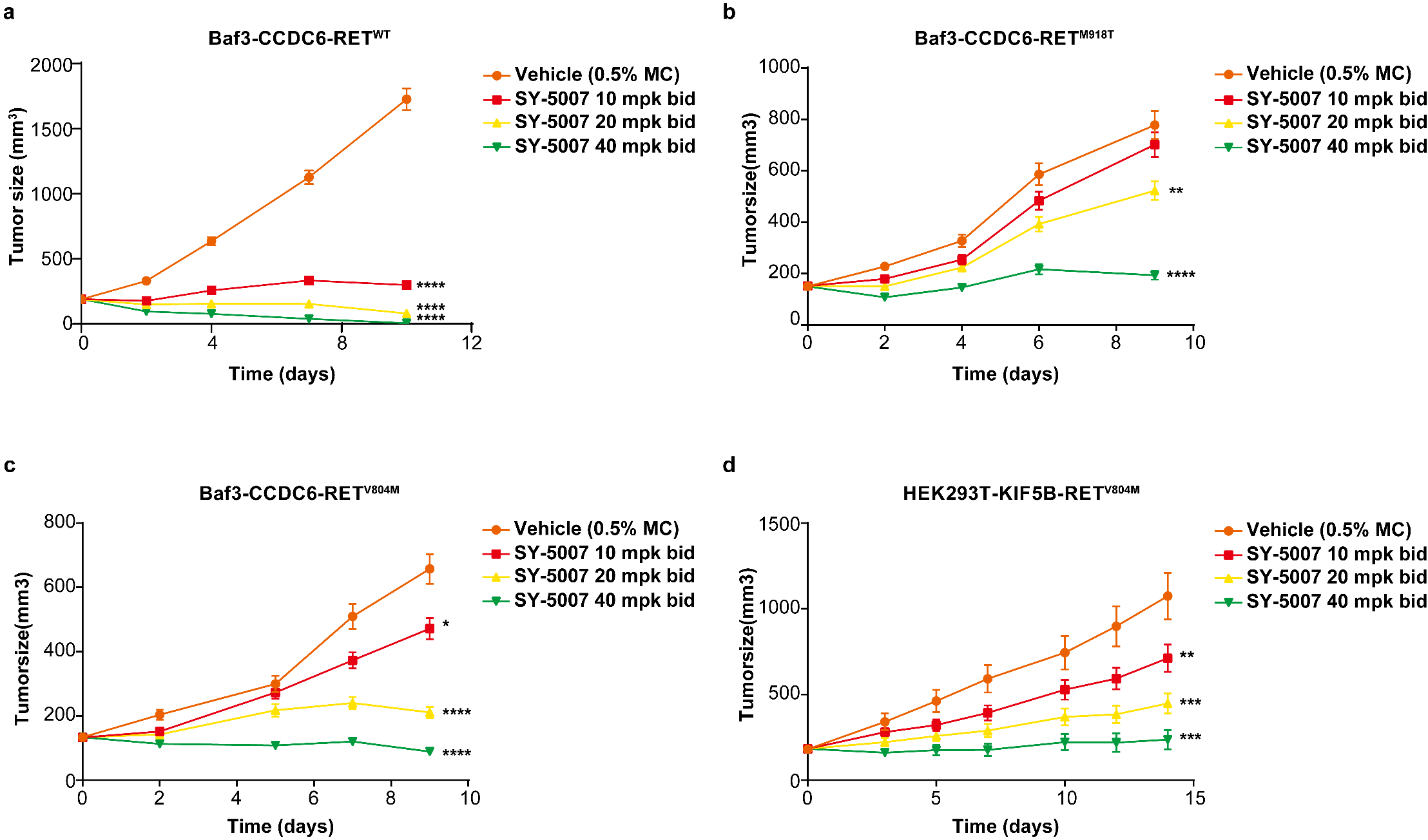


**Supplementary Figure S1. *In vivo* Efficacy of SY-5007 in RET-driven tumor xenografts in mice**. **a-c**, Cell lines (Baf3-CCDC6-RET^WT/ V804M/ M918T^ Cell, HEK293T-KIF5B-RET^V804M^ Cell) were implanted into subcutaneous flanks of female mice and treated as indicated. Mice bearing Baf3-CCDC6-RET^WT^ (**a**), Baf3-CCDC6-RET^M918T^ (**b**) and Baf3-CCDC6-RET^V804M^ (**c**) xenograft and time course of treatment. Data represent mean ± standard error of the mean (SEM). There were 12 mice in each group in both models. **d**, Mice bearing HEK293T-KIF5B-RET^V804M^ xenograft and time course of treatment. Data represent mean ± SEM. There were 10 mice in each group in this model. The volume of tumors in all treatment groups in each model was significantly lower than that of the respective vehicle-treated groups. Statistical analysis was performed using a two-way ANOVA test. *, *p* < 0.5; **, *p* < 0.01; ***, *p* < 0.001; ****, *p* < 0.0001. Abbreviations: bid, bis in die; mc, methyl cellulose; mpk, mg/Kg.

Figure. S2.


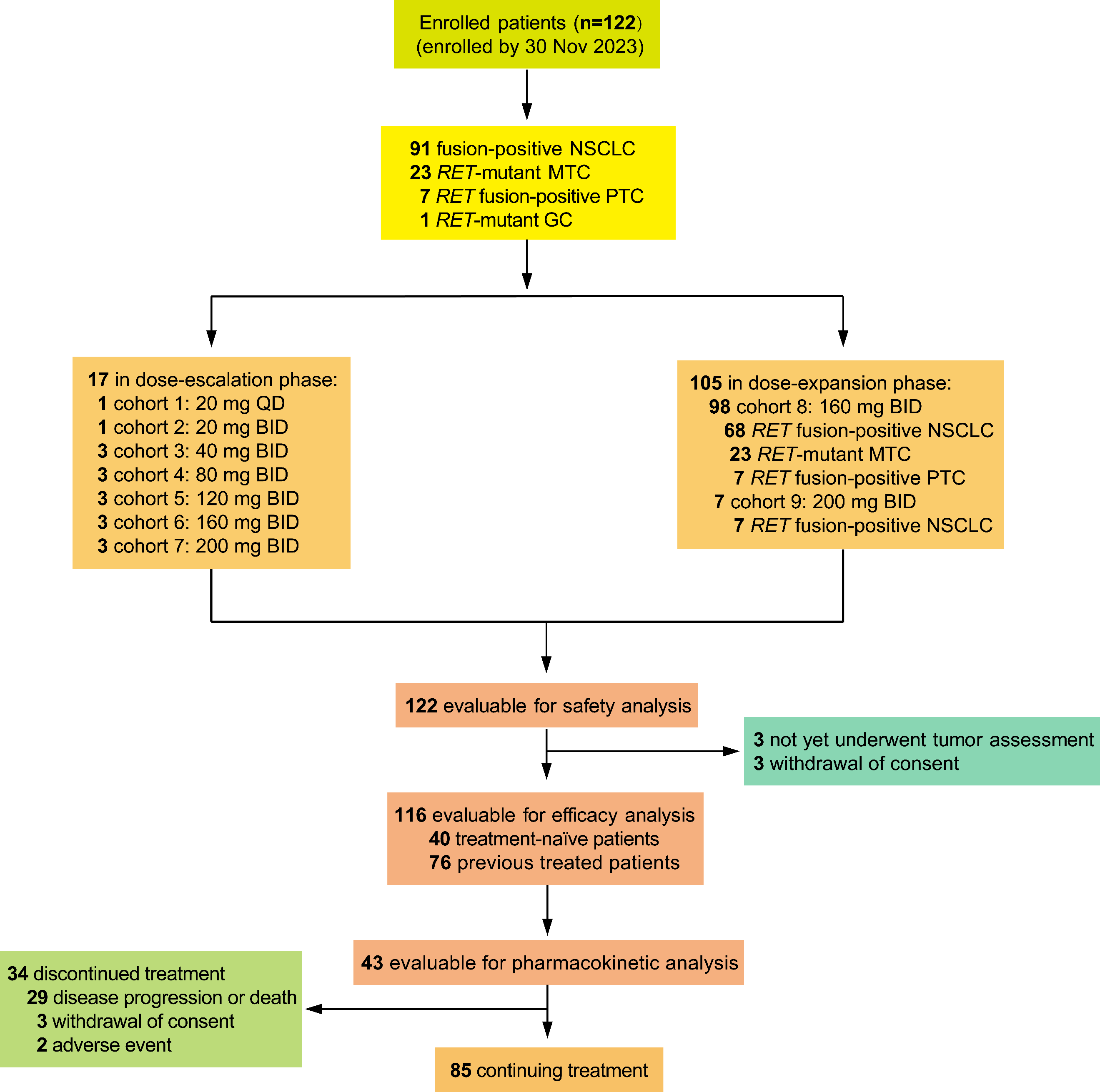


**Supplementary Figure S2. Patients disposition**. Abbreviations: BID, bis in die; GC, gastric cancer; MTC, medullary thyroid cancer; NSCLC, non-small-cell lung cancer; PTC, papillary thyroid carcinoma; QD, quaque die.

Figure. S3.


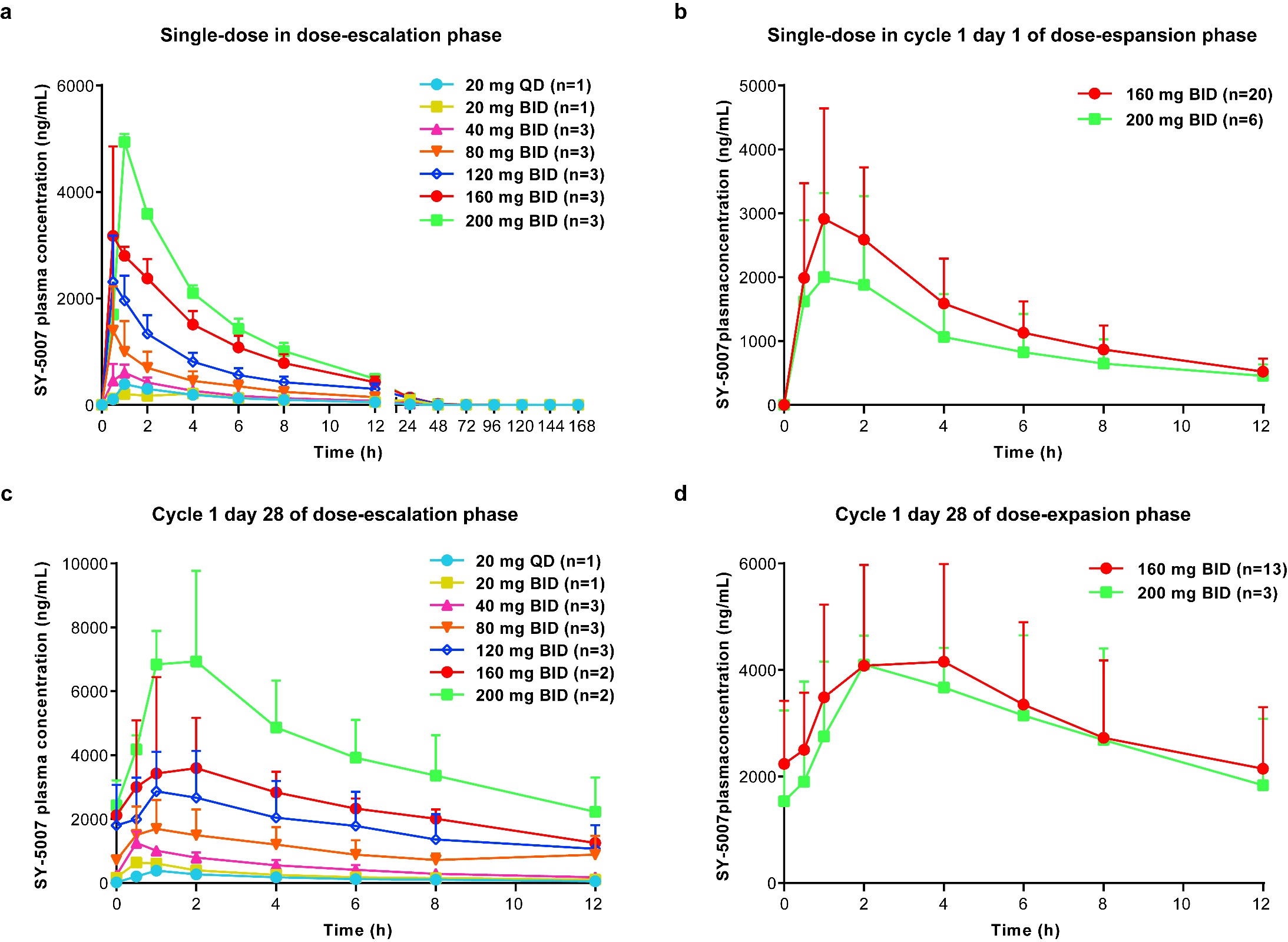


**Supplementary Figure S3. Mean concentration-time profiles of SY-5007 in phase 1 trial.** **a**. Single-dose SY-5007 plasma concentration-time curve assessed in the PK lead-in phase of the dose-escalation phase at pre-dose and 0.5, 1, 2, 3, 4, 6, 8, 12, 24, 48, 72, 96, 120, 144, and 168 h post-dose. **b**. Plasma concentration-time curve of single-dose SY-5007 PK assessed in Cycle 1 day 1 of the dose-expansion phase at pre-dose and 0.5, 1, 2, 3, 4, 6, 8 and 12 h post-dose. **c**. Plasma concentration-time curve of SY-5007 PK assessed in Cycle 1 day 28 of the dose-escalation phase at pre-dose and 0.5, 1, 2, 3, 4, 6, 8 and 12 h post-dose. **d**. Plasma concentration-time curve of SY-5007 PK assessed in Cycle 1 day 28 of the dose-expansion phase at pre-dose and 0.5, 1, 2, 3, 4, 6, 8 and 12 h post-dose. Data are presented as mean + SEM. Abbreviations: BID, bis in die; PK, pharmacokinetic; QD, quaque die.

Figure. S4.


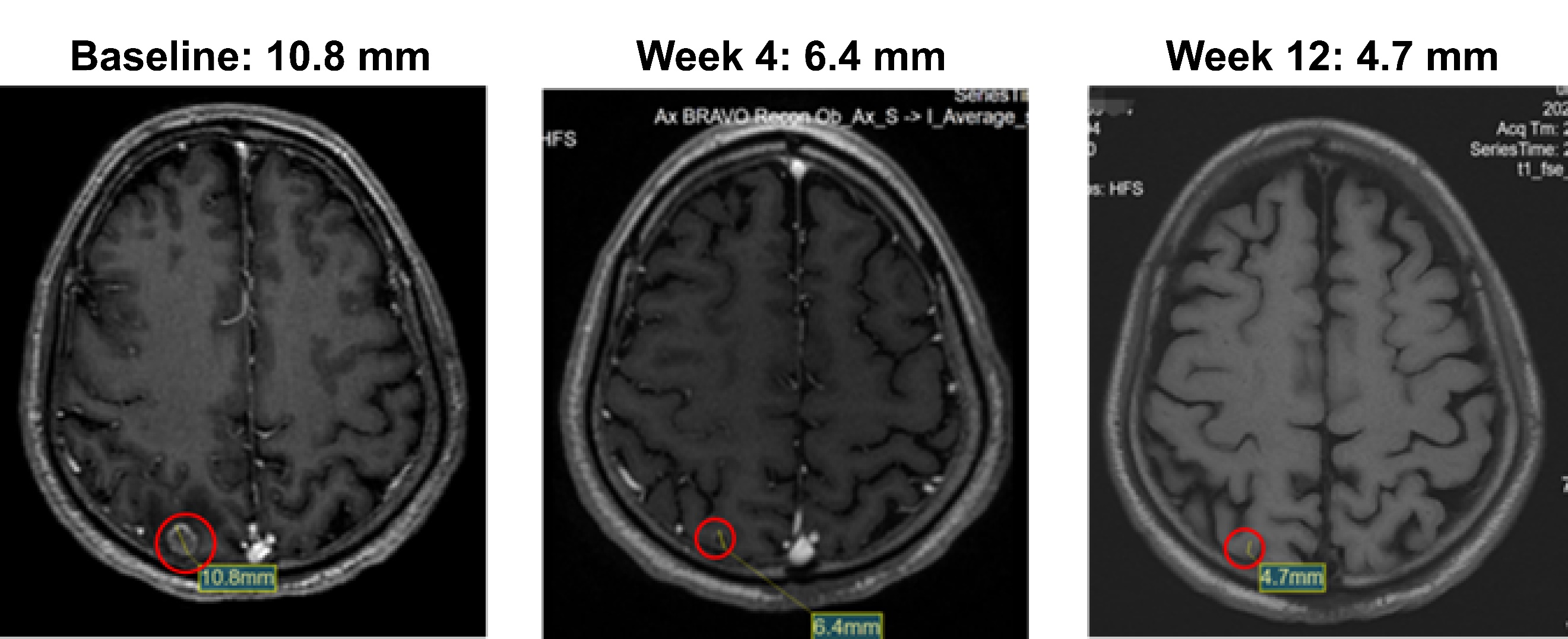


**Supplementary Figure S4. Intracranial response to SY-5007.** MRI scan of intracranial lesion (red cycle) at baseline (left) and after 4 weeks (middle) and 12 weeks (right) of SY-5007 treatment.

Figure. S5.


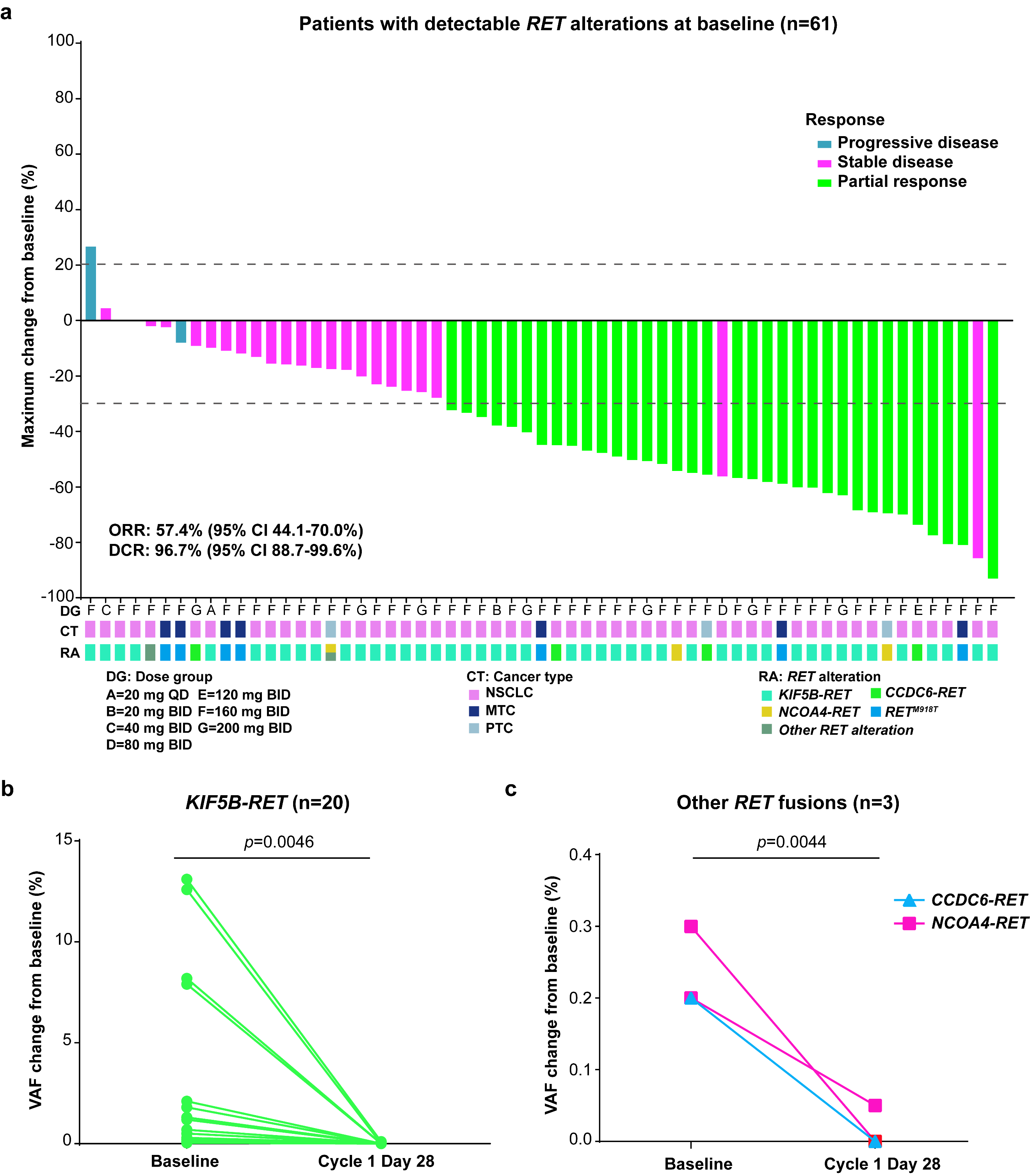


**Supplementary Figure S5. Efficacy of SY-5007 in patients with detectable *RET* alterations in ctDNA at baseline.** **a**. Waterfall plots of the maximum change in tumor size in 61 patients with detectable *RET* alterations in ctDNA at baseline. **b**, **c**. Plasma samples were tested for *KIF5B-RET* (**b**) and other RET fusions (*CCDC6-RET* and *NCOA4-RET*) (**c**) in ctDNA at baseline and Cycle 1 Day 28, each line represents one patient and this analysis included 23 patients with detectable *RET* fusion in ctDNA. Two patients, one with *KIF5B-RET*, and the other with *CCDC6-RET*, had no measurable lesions and are not shown in the above waterfall plots. Statistical analysis was performed using unpaired t-test and *p* value are shown. Abbreviations: BID, bis in die; ctDNA, circulating tumor DNA; DCR, disease control rate; MTC, medullary thyroid cancer; NSCLC, non-small cell lung cancer; ORR, objective response rate; PTC, papillary thyroid carcinoma; QD, quaque die.

Table S1.

***In Vitro* enzymatic activity of SY-5007 on RET**

|  | **Biochemical IC_50_, (nM)** | | | | Ratio (RET^WT^/ VEGFR2) |
| --- | --- | --- | --- | --- | --- |
| **Compound** | RET^WT^ | RET^V804M^ | RET^M918T^ | RET^G810S^ |  |
| **SY-5007** | 0.339 | 0.564 | 0.84 | 2.07 | 16.1 |

Abbreviation: IC_50_, half-maximal inhibitory concentration.

Table S2.

***In Vitro* anti-proliferative activity of SY-5007 in Cells**

| **Compound** | **Cellular Viability IC_50_, (nM)** | | | | | | | | | | | | |
| --- | --- | --- | --- | --- | --- | --- | --- | --- | --- | --- | --- | --- | --- |
|  | TT | HEK293T-KIF5B-RET | | |  | Baf3-KIF5B-RET | | |  | Baf3-CCDC6-RET | | | NIH-3T3 |
|  | (RET^C634W^) | WT | V804M | M918T |  | WT | V804M | M918T |  | WT | V804M | M918T |  |
| **SY-5007** | 9.11 | 54.4 | 99 | 57 |  | 9.65 | 45.3 | 13.6 |  | 10.2 | 27.1 | 5.44 | >5000 |

Abbreviation: IC_50_, half-maximal inhibitory concentration.

Table S3.

***RET* gene alternation and follow-up information for patients in phase 1**

| **Number** | **Patient ID** | **Phase** | **Tumor Type** | **Dose** | ***RET* Gene Alternation** | **PFS: Type of event** | **PFS Follow-up (m)** |
| --- | --- | --- | --- | --- | --- | --- | --- |
| **1** | S011001 | Dose-Escalation | NSCLC | 20mg QD | KIF5B-RET | Censoring | 4.83 |
| **2** | S011002 | Dose-Escalation | NSCLC | 20mg BID | KIF5B-RET | Event | 27.14 |
| **3** | S011003 | Dose-Escalation | NSCLC | 40mg BID | Unknown RET Fusion | Event | 25.07 |
| **4** | S011004 | Dose-Escalation | NSCLC | 40mg BID | KIF5B-RET | Censoring | 10.29 |
| **5** | S011005 | Dose-Escalation | NSCLC | 40mg BID | KIF5B-RET | Censoring | 1.68 |
| **6** | S011006 | Dose-Escalation | NSCLC | 80mg BID | KIF5B-RET | Censoring | 6.64 |
| **7** | S011007 | Dose-Escalation | NSCLC | 80mg BID | CCDC6-RET | Event | 23.23 |
| **8** | S011008 | Dose-Escalation | NSCLC | 120mg BID | KIF5B-RET | Event | 21.39 |
| **9** | S011009 | Dose-Escalation | NSCLC | 120mg BID | CCDC6-RET | Event | 21.42 |
| **10** | S011010 | Dose-Escalation | NSCLC | 120mg BID | CCDC6-RET | Censoring | 21.13 |
| **11** | S011011 | Dose-Escalation | NSCLC | 160mg BID | CCDC6-RET | Event | 19.55 |
| **12** | S011012 | Dose-Escalation | NSCLC | 160mg BID | KIF5B-RET | Censoring | 15.41 |
| **13** | S012001 | Dose-Expansion | NSCLC | 160mg BID | KIF5B-RET | Censoring | 13.90 |
| **14** | S012002 | Dose-Expansion | NSCLC | 160mg BID | CCDC6-RET | Event | 13.80 |
| **15** | S012003 | Dose-Expansion | NSCLC | 160mg BID | NCOA4-RET | Event | 13.83 |
| **16** | S012004 | Dose-Expansion | NSCLC | 200mg BID | KIF5B-RET | Event | 11.99 |
| **17** | S012005 | Dose-Expansion | NSCLC | 200mg BID | CCDC6-RET | Event | 11.96 |
| **18** | S012007 | Dose-Expansion | NSCLC | 200mg BID | KIF5B-RET | Censoring | 5.98 |
| **19** | S012008 | Dose-Expansion | NSCLC | 200mg BID | CCDC6-RET | Censoring | 5.75 |
| **20** | S012009 | Dose-Expansion | NSCLC | 160mg BID | KIF5B-RET | Censoring | 10.09 |
| **21** | S012012 | Dose-Expansion | NSCLC | 160mg BID | KIF5B-RET | Event | 0.03 |
| **22** | S012013 | Dose-Expansion | NSCLC | 160mg BID | KIF5B-RET | Event | 10.09 |
| **23** | S012014 | Dose-Expansion | NSCLC | 160mg BID | KIF5B-RET,  BMS1-RET | Event | 10.12 |
| **24** | S012015 | Dose-Expansion | NSCLC | 160mg BID | KIF5B-RET | Censoring | 10.12 |
| **25** | S012016 | Dose-Expansion | NSCLC | 160mg BID | KIF5B-RET | Event | 8.28 |
| **26** | S012019 | Dose-Expansion | NSCLC | 160mg BID | KIF5B-RET | Event | 8.35 |
| **27** | S012020 | Dose-Expansion | NSCLC | 160mg BID | CCDC6-RET | Event | 8.28 |
| **28** | S012024 | Dose-Expansion | NSCLC | 160mg BID | KIF5B-RET | Event | 8.28 |
| **29** | S012025 | Dose-Expansion | NSCLC | 160mg BID | CCDC6-RET | Event | 6.44 |
| **30** | S012026 | Dose-Expansion | NSCLC | 160mg BID | KIF5B-RET | Event | 6.44 |
| **31** | S012027 | Dose-Expansion | NSCLC | 160mg BID | KIF5B-RET | Event | 0.03 |
| **32** | S012028 | Dose-Expansion | NSCLC | 160mg BID | CCDC6-RET | Event | 0.03 |
| **33** | S021002 | Dose-Escalation | NSCLC | 80mg BID | Unknown RET Fusion | Event | 23.30 |
| **34** | S021003 | Dose-Escalation | GC | 160mg BID | N/A | Censoring | 1.02 |
| **35** | S021004 | Dose-Escalation | NSCLC | 200mg BID | ANK3-RET | Event | 17.74 |
| **36** | S021005 | Dose-Escalation | NSCLC | 200mg BID | KIF5B-RET | Event | 17.74 |
| **37** | S022002 | Dose-Expansion | NSCLC | 160mg BID | CCDC6-RET | Censoring | 6.37 |
| **38** | S022005 | Dose-Expansion | MTC | 160mg BID | RET p.I590_G607del | Event | 14.72 |
| **39** | S022006 | Dose-Expansion | MTC | 160mg BID | RET p.M918T | Event | 6.44 |
| **40** | S022007 | Dose-Expansion | MTC | 160mg BID | RET p.M918T | Event | 11.96 |
| **41** | S022008 | Dose-Expansion | NSCLC | 160mg BID | KIF5B-RET | Censoring | 9.23 |
| **42** | S022009 | Dose-Expansion | NSCLC | 160mg BID | KIF5B-RET | Event | 10.15 |
| **43** | S022010 | Dose-Expansion | NSCLC | 160mg BID | KIF5B-RET | Event | 8.28 |
| **44** | S022011 | Dose-Expansion | NSCLC | 160mg BID | KIF5B-RET | Event | 0.03 |
| **45** | S022013 | Dose-Expansion | NSCLC | 160mg BID | KIF5B-RET | Event | 8.25 |
| **46** | S022014 | Dose-Expansion | MTC | 160mg BID | RET p.M918T | Event | 6.51 |
| **47** | S022015 | Dose-Expansion | NSCLC | 160mg BID | KIF5B-RET | Event | 0.03 |
| **48** | S022018 | Dose-Expansion | NSCLC | 160mg BID | KIF5B-RET | Event | 4.63 |
| **49** | S022020 | Dose-Expansion | NSCLC | 160mg BID | KIF5B-RET | Event | 2.76 |
| **50** | S022021 | Dose-Expansion | NSCLC | 160mg BID | KIF5B-RET | Event | 2.76 |
| **51** | S051001 | Dose-Escalation | NSCLC | 200mg BID | KIF5B-RET | Event | 3.15 |
| **52** | S052001 | Dose-Expansion | NSCLC | 160mg BID | KIF5B-RET | Censoring | 15.58 |
| **53** | S052002 | Dose-Expansion | NSCLC | 160mg BID | KIF5B-RET | Event | 2.73 |
| **54** | S052003 | Dose-Expansion | NSCLC | 160mg BID | CCDC6-RET,  ANK3-RET | Event | 15.74 |
| **55** | S052004 | Dose-Expansion | MTC | 160mg BID | RET p.M918T | Event | 13.90 |
| **56** | S052007 | Dose-Expansion | NSCLC | 160mg BID | KIF5B-RET | Censoring | 4.44 |
| **57** | S052008 | Dose-Expansion | NSCLC | 160mg BID | KIF5B-RET | Censoring | 4.70 |
| **58** | S052009 | Dose-Expansion | NSCLC | 160mg BID | KIF5B-RET | Censoring | 13.80 |
| **59** | S052014 | Dose-Expansion | NSCLC | 200mg BID | KIF5B-RET | Censoring | 6.47 |
| **60** | S052015 | Dose-Expansion | NSCLC | 200mg BID | KIF5B-RET | Event | 12.16 |
| **61** | S052016 | Dose-Expansion | NSCLC | 160mg BID | KIF5B-RET | Censoring | 10.15 |
| **62** | S052017 | Dose-Expansion | NSCLC | 160mg BID | KIF5B-RET | Event | 10.25 |
| **63** | S052018 | Dose-Expansion | NSCLC | 160mg BID | KIF5B-RET | Event | 10.32 |
| **64** | S052019 | Dose-Expansion | NSCLC | 160mg BID | RAB18-RET,  HSD3B1-RET | Event | 10.32 |
| **65** | S052020 | Dose-Expansion | NSCLC | 160mg BID | KIF5B-RET | Censoring | 2.69 |
| **66** | S052021 | Dose-Expansion | MTC | 160mg BID | RET p.C634R | Event | 8.41 |
| **67** | S052022 | Dose-Expansion | PTC | 160mg BID | CCDC6-RET | Event | 8.38 |
| **68** | S052023 | Dose-Expansion | PTC | 160mg BID | NCOA4-RET | Event | 8.48 |
| **69** | S052024 | Dose-Expansion | MTC | 160mg BID | RET p.M918T | Censoring | 4.57 |
| **70** | S052025 | Dose-Expansion | MTC | 160mg BID | RET p.M918T | Event | 8.51 |
| **71** | S052026 | Dose-Expansion | MTC | 160mg BID | RET p.C618G | Event | 6.51 |
| **72** | S052027 | Dose-Expansion | MTC | 160mg BID | RET p.M918T | Event | 6.60 |
| **73** | S052028 | Dose-Expansion | NSCLC | 160mg BID | KIF5B-RET | Event | 6.57 |
| **74** | S052029 | Dose-Expansion | NSCLC | 160mg BID | CCDC6-RET | Event | 6.57 |
| **75** | S052031 | Dose-Expansion | PTC | 160mg BID | TRIM27-RET | Event | 6.67 |
| **76** | S052032 | Dose-Expansion | MTC | 160mg BID | RET p.M918T | Event | 4.60 |
| **77** | S052037 | Dose-Expansion | PTC | 160mg BID | CCDC6-RET | Event | 2.89 |
| **78** | S052038 | Dose-Expansion | MTC | 160mg BID | RET p.M918T | Event | 2.86 |
| **79** | S052039 | Dose-Expansion | PTC | 160mg BID | Unknown RET Fusion | Event | 2.92 |
| **80** | S052040 | Dose-Expansion | NSCLC | 160mg BID | KIF5B-RET | Event | 4.67 |
| **81** | S052041 | Dose-Expansion | MTC | 160mg BID | RET p.A883F | Event | 0.99 |
| **82** | S052042 | Dose-Expansion | NSCLC | 160mg BID | CCDC6-RET | Event | 2.96 |
| **83** | S052044 | Dose-Expansion | NSCLC | 160mg BID | KIF5B-RET | Event | 0.89 |
| **84** | S062002 | Dose-Expansion | NSCLC | 160mg BID | KIF5B-RET | Event | 13.77 |
| **85** | S062003 | Dose-Expansion | NSCLC | 160mg BID | KIF5B-RET | Censoring | 0.76 |
| **86** | S062004 | Dose-Expansion | NSCLC | 160mg BID | Unknown RET Fusion | Censoring | 0.79 |
| **87** | S062006 | Dose-Expansion | NSCLC | 160mg BID | KIF5B-RET | Censoring | 6.21 |
| **88** | S062007 | Dose-Expansion | NSCLC | 160mg BID | KIF5B-RET | Event | 10.12 |
| **89** | S062008 | Dose-Expansion | NSCLC | 160mg BID | KIF5B-RET | Censoring | 4.37 |
| **90** | S062009 | Dose-Expansion | NSCLC | 160mg BID | KIF5B-RET | Event | 6.28 |
| **91** | S062010 | Dose-Expansion | NSCLC | 160mg BID | PRKAR1A-RET | Event | 6.34 |
| **92** | S072001 | Dose-Expansion | NSCLC | 160mg BID | KIF5B-RET | Event | 8.48 |
| **93** | S082001 | Dose-Expansion | NSCLC | 160mg BID | KIF5B-RET | Event | 15.87 |
| **94** | S082002 | Dose-Expansion | NSCLC | 160mg BID | KIF5B-RET | Event | 13.87 |
| **95** | S082004 | Dose-Expansion | NSCLC | 200mg BID | KIF5B-RET | Event | 0.89 |
| **96** | S082005 | Dose-Expansion | NSCLC | 160mg BID | CCDC6-RET | Event | 10.02 |
| **97** | S082006 | Dose-Expansion | MTC | 160mg BID | RET p.C611R | Event | 8.38 |
| **98** | S082007 | Dose-Expansion | MTC | 160mg BID | RET p.M918T | Event | 5.75 |
| **99** | S092001 | Dose-Expansion | MTC | 160mg BID | RET p.M918T | Censoring | 0.99 |
| **100** | S092002 | Dose-Expansion | MTC | 160mg BID | RET p.M918T | Event | 2.76 |
| **101** | S092003 | Dose-Expansion | MTC | 160mg BID | RET p.M918T | Event | 2.66 |
| **102** | S112002 | Dose-Expansion | PTC | 160mg BID | NCOA4-RET | Event | 8.35 |
| **103** | S112004 | Dose-Expansion | NSCLC | 160mg BID | CCDC6-RET | Event | 8.28 |
| **104** | S112005 | Dose-Expansion | NSCLC | 160mg BID | CCDC6-RET | Event | 8.28 |
| **105** | S112006 | Dose-Expansion | NSCLC | 160mg BID | KIF5B-RET | Event | 4.50 |
| **106** | S112008 | Dose-Expansion | NSCLC | 160mg BID | KIF5B-RET | Event | 8.31 |
| **107** | S112009 | Dose-Expansion | NSCLC | 160mg BID | KIF5B-RET | Censoring | 6.44 |
| **108** | S112010 | Dose-Expansion | NSCLC | 160mg BID | KIF5B-RET | Event | 8.28 |
| **109** | S112011 | Dose-Expansion | NSCLC | 160mg BID | KIF5B-RET | Event | 6.47 |
| **110** | S112012 | Dose-Expansion | MTC | 160mg BID | RET p.M918T | Event | 6.41 |
| **111** | S112013 | Dose-Expansion | MTC | 160mg BID | RET p.M918T | Event | 6.41 |
| **112** | S112014 | Dose-Expansion | NSCLC | 160mg BID | KIF5B-RET | Event | 6.44 |
| **113** | S112015 | Dose-Expansion | NSCLC | 160mg BID | KIF5B-RET | Event | 4.57 |
| **114** | S112016 | Dose-Expansion | MTC | 160mg BID | RET p.C634W | Event | 4.60 |
| **115** | S112018 | Dose-Expansion | PTC | 160mg BID | NCOA4-RET | Event | 2.79 |
| **116** | S112019 | Dose-Expansion | NSCLC | 160mg BID | Unknown RET Fusion | Event | 0.92 |
| **117** | S112021 | Dose-Expansion | NSCLC | 160mg BID | KIF5B-RET | Event | 0.03 |
| **118** | S122001 | Dose-Expansion | NSCLC | 160mg BID | KIF5B-RET | Event | 2.76 |
| **119** | S122002 | Dose-Expansion | NSCLC | 160mg BID | KIF5B-RET | Event | 2.73 |
| **120** | S122003 | Dose-Expansion | MTC | 160mg BID | RET p.L769L | Event | 0.85 |
| **121** | S122004 | Dose-Expansion | NSCLC | 160mg BID | KIF5B-RET | Event | 0.89 |
| **122** | S132001 | Dose-Expansion | MTC | 160mg BID | RET p.C634Y | Censoring | 0.92 |

Abbreviations: BID, bis in die; MTC, medullary thyroid cancer; N/A, not available; NSCLC, non-small-cell lung cancer; PFS, progression-free survival; PTC, papillary thyroid carcinoma; QD, quaque die.

Table S4.

**Baseline characteristics in patients at RP2D**

|  | **NSCLC (n=70)** | **PTC (n=7)** | **MTC (n=23)** |
| --- | --- | --- | --- |
| **Median age (range)** | 59.0 (34-79) | 50.0 (31-66) | 54.0 (28-65) |
| **Sex, n (%)** |  |  |  |
| Male | 32 (45.7) | 2 (28.6) | 15 (65.2) |
| Female | 38 (54.3) | 5 (71.4) | 8 (34.8) |
| **ECOG PS, n (%)^a^** |  |  |  |
| 0 | 16 (22.9) | 0 (0.0) | 12 (52.2) |
| 1 | 54 (77.1) | 7 (100.0) | 11 (47.8) |
| **Smoking history, n (%)** |  |  |  |
| Current/Prior | 50 (71.4) | 6 (85.7) | 17 (73.9) |
| Never | 20 (28.6) | 1 (14.3) | 6 (26.1) |
| **Brain metastases, n (%)** | 19 (27.1) | 1 (14.3) | 0 (0.0) |
| ***RET*-testing method, n (%)^b^** |  |  |  |
| NGS | 31 (44.3) | 4 (57.1) | 17 (73.9) |
| RT-PCR | 16 (22.9) | 0 (0.0) | 2 (8.7) |
| Other^c^ | 23 (32.9) | 3 (42.9) | 4 (17.4) |
| ***RET* fusion, n (%)** |  |  |  |
| *KIF5B-RET* | 51 (72.9) | 0 (0.0) | 0 (0.0) |
| *CCDC6-RET* | 12 (17.1) | 2 (28.6) | 0 (0.0) |
| *NCOA4-RET* | 1 (1.4) | 3 (42.9) | 0 (0.0) |
| Other^d^ | 4 (5.7) | 1 (14.3) | 0 (0.0) |
| Unknown | 1 (1.4) | 1 (14.3) | 0 (0.0) |
| ***RET* mutation, n (%)** |  |  |  |
| M918T | 0 (0.0) | 0 (0.0) | 15 (65.2) |
| Other^e^ | 0 (0.0) | 0 (0.0) | 8 (34.8) |
| **Treatment naïve, n (%)** | 32 (45.7) | 1 (14.3) | 8 (34.8) |
| **Prior therapy type, n (%)** |  |  |  |
| Chemotherapy | 34 (48.6) | 1 (14.3) | 1 (4.3) |
| Multikinase inhibitor | 10 (14.3) | 5 (71.4) | 14 (60.9) |
| PD-(L)1 inhibitor | 14 (20.0) | 1 (14.3) | 0 (0.0) |
| Other systemic therapies^f^ | 19 (27.1) | 1 (14.3) | 0 (0.0) |
| **Prior cancer-related surgery, n (%)** | 13 (18.6) | 4 (57.1) | 12 (52.2) |

Abbreviations: ECOG PS, Eastern Cooperative Oncology Group performance status; GC, gastric cancer; MTC, medullary thyroid cancer; NGS, next-generation sequencing; NSCLC, non-small-cell lung cancer; PD-(L)1, programmed death-1 (PD-1)/programmed death ligand-1 (PD-L1); PTC, papillary thyroid carcinoma; RT-PCR, reverse transcription-polymerase chain reaction.

^a^ECOG PS scores range from 0 to 5, with higher scores indicating greater disability.

^b^Fusion status was assayed by multiple techniques in some patients.

^c^Other test methods included amplification refractory mutation system- polymerase chain reaction (ARMS-PCR), fluorescence *in situ* hybridization (FISH) and immunohistochemistry (IHC).

^d^Other fusions included *ANK3-RET*, *TRIM27-RET*, *PRKAR1A-RET*, and *BMS1-RET*.

^e^Other mutations included C634R/W/Y, A883F, C816G and C611R.

^f^Other systemic therapies included Chinese medicine, radiotherapy and recombinant human endostatin.

Table S5.

**Efficacy of SY-5007 in patients at RP2D**

|  | **NSCLC** | **MTC** | **PTC** |
| --- | --- | --- | --- |
| **All patients (n)** | 64 | 23 | 7 |
| Best Response, n (%) |  |  |  |
| Partial response | 41 (64.1) | 12 (52.2) | 3 (42.9) |
| Stable disease | 21 (32.8) | 9 (39.1) | 4 (57.1) |
| Progressive disease | 2 (3.1) | 2 (8.7) | 0 (0.0) |
| ORR, % (95% CI) | 64.1 (51.1-75.7) | 52.2 (30.6-73.2) | 42.9 (9.9-81.6) |
| DCR, % (95% CI) | 96.9 (89.2-99.6) | 91.3 (72.0-98.9) | 100.0 (59.0-100.0) |
| Median TTR, months (95% CI) | 2.75 (1.01-2.89) | 4.66 (2.75-NE) | NE (0.88-NE) |
| Median DoR, months (95% CI) | 13.0 (9.26-NE) | NE (NE-NE) | NE (NE-NE) |
| Median PFS, months (95% CI) | 15.4 (10.1-NE) | NE (NE-NE) | NE (NE-NE) |
| Median follow-up, months (95% CI) | 8.28 (6.47-10.0) | 6.44 (4.60-8.37) | 6.67 (2.79-8.37) |
| **Treatment-naïve patients (n)** | 28 | 8 | 1 |
| Best Response, n (%) |  |  |  |
| Partial response | 20 (71.4) | 6 (75.0) | 0 (0.0) |
| Stable disease | 8 (28.6) | 0 (0.0) | 1 (100.0) |
| Progressive disease | 0 (0.0) | 2 (25.0) | 0 (0.0) |
| ORR, % (95% CI) | 71.4 (51.3-86.8) | 75.0 (34.9-96.8) | 0 (0.0-97.5) |
| DCR, % (95% CI) | 100.0 (87.7-100.0) | 75.0 (34.9-96.8) | 100.0 (2.5-100.0) |
| Median TTR, months (95% CI) | 1.11 (0.91-3.15) | 2.75 (0.85-NE) | NE (NE-NE) |
| Median DoR, months (95% CI) | 14.5 (9.26-NE) | NE (NE-NE) | NE (NE-NE) |
| Median PFS, months (95% CI) | 15.5 (9.23-NE) | NE (0.92-NE) | NE (NE-NE) |
| Median follow-up, months (95% CI) | 8.28 (6.27-10.0) | 6.44 (0.85-NE) | 2.92 (NE- -NE) |
| **Previously Treated patients (n)** | 36 | 15 | 6 |
| Best Response, n (%) |  |  |  |
| Partial response | 21 (58.3) | 6 (40.0) | 3 (50.0) |
| Stable disease | 13 (36.1) | 9 (60.0) | 3 (50.0) |
| Progressive disease | 2 (5.6) | 0 (0.0) | 0 (0.0) |
| ORR, % (95% CI) | 58.3 (40.8-74.5) | 40.0 (16.3-67.7) | 50.0 (11.8-88.2) |
| DCR, % (95% CI) | 94.4 (81.3-99.3) | 100.0 (78.2-100.0) | 100.0 (54.1-100.0) |
| Median TTR, months (95% CI) | 2.75 (0.98-NE) | NE (2.75-NE) | NE (0.88-NE) |
| Median DoR, months (95% CI) | 13.0 (9.20-NE) | NE (NE-NE) | NE (NE-NE) |
| Median PFS, months (95% CI) | 13.8 (10.1-NE) | NE (NE-NE) | NE (NE-NE) |
| Median follow-up, months (95% CI) | 8.28 (6.44-10.3) | 6.50 (4.60-8.51) | 7.50 (2.79-NE) |

Abbreviations: BID, bis in die; CI, confidence interval; DCR, disease control rate; DoR, duration of response; MTC, medullary thyroid cancer; NE, not evaluated; NSCLC, non-small-cell lung cancer; ORR, objective response rate; PFS, progression-free survival; PTC, papillary thyroid carcinoma; QD, quaque die; TTR, time to response.

Table S6.

**Efficacy of SY-5007 in patients with baseline detectable or undetectable *RET* alterations**

|  | **Undetectable *RET* alterations** | **Detectable *RET* alterations** | **All patients** |
| --- | --- | --- | --- |
| Best overall response |  |  |  |
| Number of patients (n) | 53 | 61 | 114 |
| Partial response | 32 (60.4) | 35 (57.4) | 67 (58.8) |
| Stable disease | 18 (34.0) | 24 (39.3) | 42 (36.8) |
| Progressive disease | 3 (5.7) | 2 (3.3) | 5 (4.4) |
| ORR, % (95% CI) | 60.4 (46.0-73.5) | 57.4 (44.1-70.0) | 58.8 (49.2-67.9) |
| DCR, % (95% CI) | 94.3 (84.3-98.8) | 96.7 (88.7-99.6) | 95.6 (90.1-98.6) |
| *p*-value of ORR (undetectable *vs.* detectable) | 0.8491 | |  |
| Median TTR, months (95% CI) | 3.15 (2.75-6.30) | 2.79 (1.01-6.73) | 2.79 (2.75-4.66) |
| Median DoR, months (95% CI) | NE (NE-NE) | 13.0 (9.23-19.9) | 19.9 (12.8-NE) |
| Median PFS, months (95% CI) | NE (NE-NE) | 13.8 (10.0-15.5) | 21.1 (13.8-NE) |
| Median follow-up, months (95% CI) | 8.28 (6.50-10.1) | 8.47 (8.24-10.2) | 8.34 (8.28-10.1) |
| *p*-value of PFS (undetectable *vs.* detectable) | 0.0011 | |  |

Abbreviations: CI, confidence interval; DCR, disease control rate; DoR, duration of response; NE, not evaluated; ORR, objective response rate; PFS, progression-free survival; TTR, time to response.

Table S7.

**Efficacy of SY-5007 in patients with baseline detectable *RET* alterations stratified by concomitant *TP53* mutation status**

|  | **Patients with detectable *RET* variations** | | | | |
| --- | --- | --- | --- | --- | --- |
|  | ***TP53* wild-type** | | ***TP53* mutant** | | **All patients** |
| Best overall response |  | |  | |  |
| Number of patients (n) | 39 | | 22 | | 61 |
| Partial response | 24 (61.5) | | 11 (50.0) | | 35 (57.4) |
| Stable disease | 13 (33.3) | | 11 (50.0) | | 24 (39.3) |
| Progressive disease | 2 (5.1) | | 0 (0.0) | | 2 (3.3) |
| ORR, % (95% CI) | 61.5 (44.6-76.6) | | 50.0 (28.2-71.8) | | 57.4 (44.1-70.0) |
| DCR, % (95% CI) | 94.9 (82.7-99.4) | | 100.0 (84.6-100.0) | | 96.7 (88.7-99.6) |
| *p*-value of ORR (*TP53* mutant *vs.* *TP53* wild-type) | 0.4277 | | | |  |
| Median TTR, months (95% CI) | 2.79 (0.98-6.73) | | 2.89 (0.95-NE) | | 2.79 (1.01-6.73) |
| Median DoR, months (95% CI) | 19.9 (6.47-NE) | | 13.0 (2.82-NE) | | 13.0 (9.23-19.9) |
| Median PFS, months (95% CI) | 15.4 (9.23-NE) | | 10.1 (6.21-NE) | | 13.8 (10.0-15.5) |
| Median PFS follow-up, months (95% CI) | 8.37 (6.44-10.1) | | 8.47 (8.28-12.1) | | 8.47 (8.24-10.2) |
| *p*-value of PFS (*TP53* mutant *vs.* *TP53* wild-type) | | 0.2412 | |  | |

Abbreviations: CI, confidence interval; DCR, disease control rate; DoR, duration of response; NE, not evaluated; ORR, objective response rate; PFS, progression-free survival; TTR, time to response.
