# Supplementary material for: First-in-human, phase 1 dose-escalation and dose-expansion study of a RET inhibitor SY-5007 in patients with advanced RET-altered solid tumors
Source: Signal Transduct Target Ther. 2024 Nov 4;9:300. doi: 10.1038/s41392-024-02006-9 (PMC11532403; doi:10.1038/s41392-024-02006-9)
Supplement: Supplementary file 2 — Study protocol [file 41392_2024_2006_MOESM2_ESM.docx]

**A Phase I Study to Evaluate the Safety, Tolerability, Pharmacokinetic Profile, and Efficacy of the RET Inhibitor SY-5007 Tablets in Subjects with Advanced Solid Tumors**

| **Clinical Stage:** | Phase I |
| --- | --- |
| **Protocol Number:** | SY-5007-I-01 |
| **Protocol Version:** | V 1.0 |
| **Version Date:** | December 01, 2020 |
| **Principal Investigator:** | Caicun Zhou |
| **Clinical Research Team Leader Unit:** | Shanghai Pulmonary Hospital |
| **Sponsor:** | Shouyao Holdings (Beijing) Co., Ltd |

| This Protocol is confidential information of Shouyao Holdings (Beijing) Co., Ltd., and shall not be reproduced or divulged to others or used for any other unauthorized purposes by any person who has obtained or viewed this material without the prior written consent of Shouyao Holdings (Beijing) Co., Ltd. If there is any violation or suspicion of violation of the above provisions, please notify Shouyao Holdings (Beijing) Co., Ltd. |
| --- |

**Protocol Signature Page**

**1. Sponsor**

I will conscientiously perform my role as sponsor in accordance with Good Clinical Trial Practice (GCP) and all applicable regulations, be responsible for initiating, applying for, organizing and financing this clinical trial, bear the relevant medical expenses and appropriate financial compensation for subjects who suffer trial-related harm or death during the clinical trial, and provide legal guarantees to the investigator. Agree to design and conduct this trial in accordance with this protocol (Protocol No: SY-5007-I-01; version number: 1.0; version date: 01 December 2020).

Sponsor: Shouyao Holdings (Beijing) Co., Ltd

Sponsor's Address: Building 15, Yuquan Huigu, No. 3 Minzhuang Road, Haidian District, Beijing

Sponsor Representative: ____________________

Signature: ______________________________

Date: _______________________________

**Protocol Signature Page**

**2. Principal investigator**

I will conscientiously fulfil my duties as a researcher in accordance with Chinese GCP regulations and personally participate in or directly supervise this clinical trial. We have read and acknowledge this protocol (protocol number: SY-5007-I-01; Version Number: 1.0; Version Date: 1 December 2020). I agree to perform my duties in accordance with Chinese law, the Declaration of Helsinki, Chinese GCP and this protocol, and to make any changes to the protocol only after notifying the sponsor, and to conduct the protocol only after obtaining the approval of the Ethics Committee, except when measures must be taken to protect the safety, rights and interests of the subjects.

Name of the test center: Shanghai Pulmonary Hospital

Principal investigator: Caicun Zhou

Signature: ______________________________

Date: ______________________________

**Contents**

[Protocol Signature Page 2](#_Toc145252343)

[Protocol summary 7](#_Toc145252344)

[List of acronyms 15](#_Toc145252345)

[1. Research background 18](#_Toc145252346)

[1.1 Introduction to the *RET* gene 18](#_Toc145252347)

[1.1.1 RET Biology 18](#_Toc145252348)

[1.1.2 Carcinogenic Activation of RET 18](#_Toc145252349)

[1.2 RET and Solid Tumors 19](#_Toc145252350)

[1.2.1 RET and Lung Cancer 19](#_Toc145252351)

[1.2.2 RET and Thyroid Cancer 20](#_Toc145252352)

[1.2.3 RET and Neuroblastoma 22](#_Toc145252353)

[1.2.4 RET in Pheochromocytoma and Hirschsprung’s disease 23](#_Toc145252354)

[1.2.5 RET and Other Tumors 23](#_Toc145252355)

[1.3 RET Inhibitors 24](#_Toc145252356)

[1.3.1 Non-selective RET Inhibitors 24](#_Toc145252357)

[1.3.2 Selective RET Inhibitors 27](#_Toc145252358)

[1.4 SY-5007 29](#_Toc145252359)

[1.4.1 Pharmacology 29](#_Toc145252360)

[1.4.2 Pharmacokinetics 33](#_Toc145252361)

[1.4.3 Toxicology 38](#_Toc145252362)

[2. Research Objectives and Evaluation Indicators 41](#_Toc145252363)

[3. Study Population 42](#_Toc145252364)

[3.1 Subject Number Rules 42](#_Toc145252365)

[3.2 Inclusion Criteria 42](#_Toc145252366)

[3.3 Exclusion Criteria 43](#_Toc145252367)

[3.4 Screening Failure 45](#_Toc145252368)

[3.5 Exit Criteria 45](#_Toc145252369)

[3.6 Early Termination of the Trial 46](#_Toc145252370)

[4. Study Design 46](#_Toc145252371)

[4.1 Overall Study Design 46](#_Toc145252372)

[4.2 Starting Dose 48](#_Toc145252373)

[4.3 DLT Definitions 50](#_Toc145252374)

[4.4 Dose Adjustment and Treatment Delays 50](#_Toc145252375)

[4.4.1 Dose-escalation phase 50](#_Toc145252376)

[4.4.2 Continuous Drug Cycle 2 and Later, Dose-expansion Phase 51](#_Toc145252377)

[5. Research Follow-up Visit 51](#_Toc145252378)

[5.1 Research Follow-up visit Process 53](#_Toc145252379)

[5.1.1 Screening Visits 61](#_Toc145252380)

[5.1.2 PK Research 61](#_Toc145252381)

[5.1.3 Study Treatment Period 64](#_Toc145252382)

[5.1.4 End-of-treatment Visit 64](#_Toc145252383)

[5.1.5 Unscheduled Visits 65](#_Toc145252384)

[5.2 Efficacy Evaluation 66](#_Toc145252385)

[5.3 Safety Evaluation 66](#_Toc145252386)

[5.3.1 Vital Signs 66](#_Toc145252387)

[5.3.2 Physical Examination (including weight) 66](#_Toc145252388)

[5.3.3 Laboratory Tests 66](#_Toc145252389)

[5.3.4 ECG Examination 67](#_Toc145252390)

[6. Experimental Drug 67](#_Toc145252391)

[6.1 Experimental Drug 67](#_Toc145252392)

[6.2 Supply, Storage and Distribution of Test Drugs 67](#_Toc145252393)

[6.3 Assessment of Treatment Adherence 68](#_Toc145252394)

[6.4 Combination of Drugs 68](#_Toc145252395)

[6.4.1 Combination of Drugs Allowed in This Study 68](#_Toc145252396)

[6.4.2 Combination of Drugs Prohibited in This Study 69](#_Toc145252397)

[7. Safety Reports and Adverse Event Management 69](#_Toc145252398)

[7.1 Related Definitions 69](#_Toc145252399)

[7.2 Severity Assessment 70](#_Toc145252400)

[7.3 Causality Assessment 71](#_Toc145252401)

[7.4 Exposure During Pregnancy 72](#_Toc145252402)

[7.5 Follow-up of AEs 73](#_Toc145252403)

[7.6 Reporting Deadlines and Requirements 73](#_Toc145252404)

[8. Data Management 74](#_Toc145252405)

[8.1 Data Entry and Modification 74](#_Toc145252406)

[8.2 External Data Management 75](#_Toc145252407)

[8.3 Data Lock 75](#_Toc145252408)

[8.4 Record Keeping 75](#_Toc145252409)

[9. Statistical Analysis 75](#_Toc145252410)

[9.1 Statistical Analysis of Data Sets 76](#_Toc145252411)

[9.2 Methods of Statistical Analysis 76](#_Toc145252412)

[9.2.1 General Analysis 76](#_Toc145252413)

[9.2.2 Demographic and baseline analysis 76](#_Toc145252414)

[9.2.3 Handling of Missing Data 77](#_Toc145252415)

[9.2.4 Security Analysis 77](#_Toc145252416)

[9.2.5 PK Analysis 77](#_Toc145252417)

[9.2.6 Efficacy Analysis 77](#_Toc145252418)

[10. Quality Control and Assurance 78](#_Toc145252419)

[11. Ethical Requirements 78](#_Toc145252420)

[11.1 Institutional Review Board (IRB)/Independent Ethics Committee (IEC) 78](#_Toc145252421)

[11.2 Ethical Implementation in Research 78](#_Toc145252422)

[11.3 Subject Information and Informed Consent 79](#_Toc145252423)

[11.4 Safety Concern Reports and Critical Program/ICH GCP Violations 79](#_Toc145252424)

[12. Sponsor's Criteria for Termination the Study 80](#_Toc145252425)

[13. Articles Published by Researchers 80](#_Toc145252426)

[14. References 82](#_Toc145252427)

[15. Appendix 90](#_Toc145252428)

[15.1 ECOG Physical Fitness Score 90](#_Toc145252429)

[15.2 Efficacy Evaluation Criteria for Solid Tumors Version 1.1 (RECIST V1.1). 90](#_Toc145252430)

[15.3 Creatinine Clearance Calculation Formula 100](#_Toc145252431)

[15.4 Examples of abstinence or contraception 100](#_Toc145252432)

[15.5 New York Heart Association Cardiac Function Classification (NYHA) 101](#_Toc145252433)

[15.6 Program Amendment Records 102](#_Toc145252434)

[15.7 Risk Prevention and Control Program 103](#_Toc145252435)

**Statement of Compliance**

1. **Follow the document**

The experiment must follow the following documents:

- National Medical Products Administration Clinical Trial Notification CXHLXXXXXXX
- The Drug Administration Law of the People's Republic of China 2019
- Measures for the Administration of Drug Registration 2020
- Good Clinical Practice for Drugs 2020
- Declaration of Helsinki 2013
- Technical Guidelines for Clinical Pharmacokinetic Research of Chemical Drugs, 2005
- Technical Guidelines for Clinical Trials of Antitumor Drugs 2012

1. **Institutional Review Board and Ethics Committees**

Prior to the start of the trial, it is the investigator's responsibility to obtain approval institutional, ethics committee, or organization with equivalent authority for this clinical study protocol, protocol revisions, informed consent, and other relevant documents (e.g., enrollment recruitment advertisements). All documents communicated with the EC should be kept in the investigator folder.

1. **informed consent**

Informed consent must comply with the requirements of GCP, ICH-GCP and relevant laws and regulations.

The informed consent form used in this study, as well as any changes made during the study, must be approved by the ethics committee and Shouyao Holdings (Beijing) Co., Ltd. (hereinafter referred to as "Shouyao Holdings") prior to use.

The benefits and risks of the trial must be fully explained to each potential subject before each potential subject participates in any trial-related activity. After explaining the essential elements of the trial and being satisfied that each subject who will participate in the trial understands the purpose of the trial, subjects should be asked to sign the informed consent form with their name and date. Investigators must provide each subject with a signed informed consent form.

**Protocol summary**

| **Teat drugs** | SY-5007 |
| --- | --- |
| **Study name** | A Phase I Study to Evaluate the Safety, Tolerability, Pharmacokinetic Profile, and Efficacy of the RET Inhibitor SY-5007 Tablets in Subjects with Advanced Solid Tumors |
| **Study number** | SY-5007-I-01 |
| **Sponsor** | Shouyao Holdings (Beijing) Co., Ltd |
| **Study design** | Single-arm, open-label, dose-escalation and dose-expansion Phase I clinical study  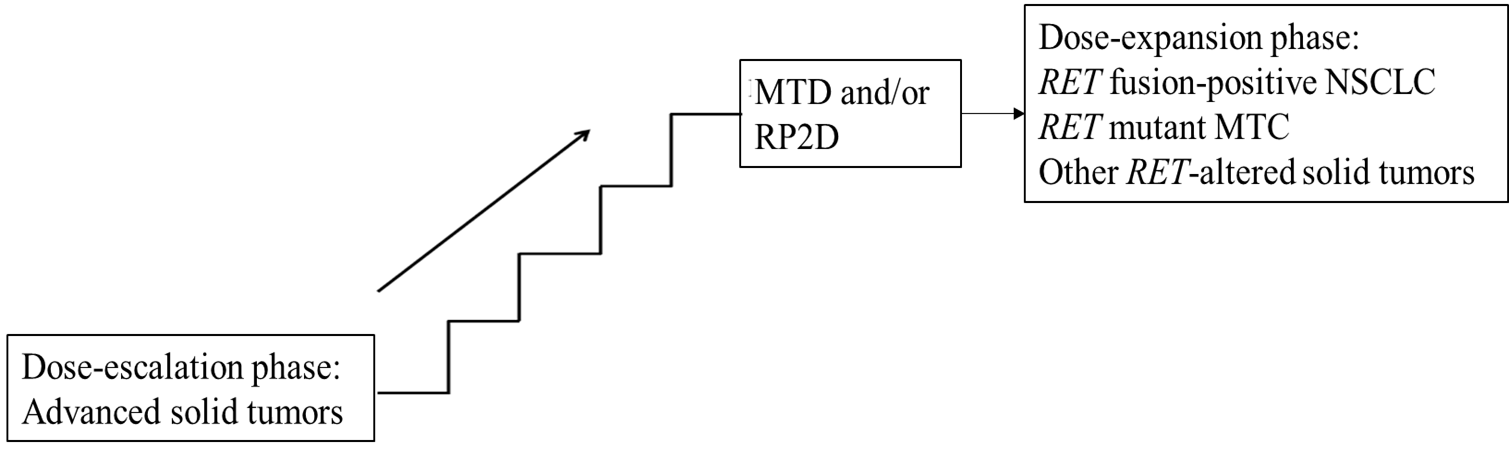  **Figure 1 Overall study design**  **Table 1 Proposed dose-escalation designs (including but not limited to the dose groups)**   \| **groups** \| **Administered dose** \| **Frequency of administration** \| **Total daily dose** \| \| --- \| --- \| --- \| --- \| \| 1 \| 20mg \| QD \| 20mg \| \| 2 \| 20mg \| BID \| 40mg \| \| 3 \| 40mg \| 80mg \| \| 4 \| 80mg \| 160mg \| \| 5 \| 120mg \| 240mg \| \| 6 \| 160mg \| 320mg \| \| 7 \| 200mg \| 400mg \|   During the dose-escalation phase, the dose level after the initial dose is escalated in steps of 100%, 100%, 50%, 33% …. according to the Fischer's modified method until ≥2/6 of subjects developed DLT at a given dose, or until a dose group whose pharmacokinetic parameters indicate that absorption is saturated, then the dose-escalation is terminated.  Based on the safety, tolerability, PK characteristics and preliminary efficacy obtained from the study, the dosage and dosing regimen for subsequent clinical trial will be determined. If the above maximum dose still fails to reach MTD, then combine with preclinical data, effective dosage observed in human in domestic and foreign studies of similar drug, and PK parameters to determine the dose and dosage regime for subsequent clinical trials after a safety review by the investigator and medical supervisors. |
| **Research object** | Advanced solid tumors |
| **Sample size** | 50~80 cases |
| **Purpose of the study** | **Primary Outcome**: To investigate the safety and tolerability of SY-5007 tablets in Chinese subjects with advanced solid tumors, and to determine the maximum tolerated dose (MTD), the limiting toxicity (DLT) and phase II recommended dose (RP2D).  **Secondary Outcome:** To evaluate the pharmacokinetic (PK) characteristics of SY-5007 tablets in Chinese subjects with advanced solid tumors, and to preliminarily evaluate the clinical efficacy of SY-5007 tablets in subjects with advanced solid tumors with RET gene fusion or mutation. |
| **Inclusion Criteria** | Subjects must meet all of the following inclusion criteria:   - 1. Age≥ 18 years old, male or female;   2. Eastern Cooperative Oncology Group (ECOG) performance status score of 0-1;   3. Estimated life expectancy >12 weeks;   4. According to RECIST V1.1, subjects must have at least one assessable lesion in dose-escalation phase and one measurable lesion in dose-expansion phase;   5. **Dose-escalation phase**: subjects with histologically or cytologically confirmed advanced solid tumors who have failed standard therapy, or for whom no standard treatment is available, or for whom standard treatment is not available at this stage (e.g., subject refuses standard therapy), and for whom there is a RET gene fusion or mutation;   **Dose-expansion phase:** subjects with histologically or cytologically confirmed advanced solid tumors who have failed standard therapy, or for whom no standard treatment is available, or for whom standard treatment is not appropriate at this stage, and who have RET gene fusion in NSCLC or RET mutation in MTC or RET-altered other advanced solid tumors;   - 1. Subjects must have adequate organ function, defined as follows:   **Liver function**:   - Without liver metastases, serum aspartate aminotransferase (AST) and serum alanine aminotransferase (ALT) ≤ 3 times the upper limit of normal (ULN); subjects with liver metastases or hepatocellular carcinoma (HCC), AST, ALT ≤ 5 times ULN, total serum bilirubin (TBIL) ≤ 1.5 times ULN.   **bone marrow function (no transfusions or hematopoietic stimulating factor therapy within 10 days prior to testing):**   - Neutrophil absolute value (ANC) ≥ 1.5×10^9^/L; - Platelets (PLT) ≥75×10^9^/L; - Hemoglobin (Hb) ≥ 85g/L.   **Kidney function**:   - Creatinine clearance ≥ 50 mL/min.   **Coagulation function**:  PT or INR≤1.5×ULN.  **Lipids**:  Cholesterol ≤ 500 mg/dL (12.92 mmol/L).   - 1. All women of childbearing age must have a negative serum pregnancy test within 7 days prior to the first dose, and male and female subjects of childbearing potential must agree to abstinence or use contraception throughout the study period and for at least 3 months after the last dose of drug;   2. Willingness and ability to give informed consent and follow protocol procedures, and comply with follow-up visit requirements. |
| **Exclusion criteria** | Subjects with any of the following are not eligible for entry into this study:   1. **Dose-expansion phase:** subjects carry known major driver gene alterations other than RET genes, e.g., EGFR, ALK, ROS1, KRAS, etc.; 2. **Dose-expansion phase:** previous use of selective RET inhibitors; 3. Received antitumor therapy such as chemotherapy, radiotherapy, biological therapy, endocrine therapy, immunotherapy and other antitumor therapy within 4 weeks prior to first dose, except for the following:   • Nitrosourea or mitomycin C within 6 weeks prior to first dose;  • Oral fluorouracil analogs and small molecule-targeted drugs 2 weeks prior to first dose or within 5 half-lives of the drug, whichever is longer;  • Traditional Chinese medicines with anti-tumor indications are within 2 weeks prior to the first dose.   1. Received other unlisted clinical trial drugs or treatments within 4 weeks prior to the first dose; 2. Major organ surgery (excluding puncture biopsy) or had significant trauma within 4 weeks prior to the first dose, or required elective surgery during the trial; 3. Adverse effects of previous antitumor therapy have not returned to a CTCAE 5.0 grade ≤ 1 (except for toxicities judged by investigator to pose no safety risk, such as alopecia, grade 2 peripheral neurotoxicity, etc.); 4. Central nervous system(CNS) metastases with clinical symptoms, or other evidence of uncontrolled CNS metastases or meningeal metastases in subjects who, in the judgement of the investigator, are not suitable for enrollment; 5. participants with active uncontrolled systemic bacterial, viral or fungal infection despite optimal treatment (not required to screen for chronic disease); 6. Active hepatitis B (HBV-DNA≥2000 IU/mL), hepatitis C virus infection (HCV antibody positive), HIV antibody positive, active syphilis, and still uncontrolled after active treatment, and is judged by the investigator to be unsuitable for enrollment; 7. History of severe cardiovascular and cerebrovascular disease, including but not limited to:   •Severe cardiac rhythm or conduction abnormalities, such as ventricular arrhythmias requiring clinical intervention, degree II-III atrioventricular block, etc;  •At rest, Mean QT interval corrected using Fridericia's formula (QTcF)> 480ms;  •Acute coronary syndrome, congestive heart failure, aortic dissection, stroke, or other grade 3 or above cardiovascular and cerebrovascular events within 6 months prior to the first dose;  • New York Heart Association (NYHA) ≥ class II heart failure or left ventricular ejection fraction (LVEF) < 50%;  • High blood pressure remains uncontrolled despite aggressive antihypertensive therapy. Uncontrolled hypertension is defined as systolic blood pressure >185 mmHg and/or diastolic blood pressure >110 mmHg measured three times at least 10 minutes apart;   1. Used any CYP3A4 inhibitor or inducer within 14 days prior to the first dose; 2. Inability to swallow the drug orally, or conditions that, in the judgment of the investigator, severely interfere with gastrointestinal absorption; 3. Subjects who, in the opinion of the investigator, have a history of other serious systemic diseases, or are otherwise reasons unsuitable for participation in this clinical study. |
| **Medication regimen** | SY-5007 tablets: 20 mg, 80 mg, the dosage and mode of administration are as prescribed by follow the physician. It is recommended that the drug should be taken at the same time every day, and the actual time of taking the drug should be based on the original clinical records. In order to prevent food from affecting drug absorption, the drug should be taken on an empty stomach, that is, 1 hour before or 2 hours after meals. If the dose is missed, it can be taken within 4 hours of the expected dose time. If the drug has not been taken for more than 4 hours, no refills will be made and the drug will be taken at the expected time. If participants vomit after taking the drug, no refills will be given.  Treatment will continue until the participant develops disease progression, intolerable toxicity, a decision by the investigator or subject to discontinue treatment, initiation of a new antineoplastic therapy, or death. |
| **Dose-limiting toxicity (DLT)** | According to the NCI CTCAE version 5.0 classification, DLT is defined as the following toxic reactions that are related (including definitely related or possibly related) within a single dose and within the 1st cycle (35 days) of continuous administration**:**  (1) Hematological toxicity:   - Grade 4 neutropenia lasting > 7 days; - grade ≥ 3 febrile neutropenia; - Grade 4 thrombocytopenia; - Grade 3 thrombocytopenia with bleeding; - Grade 4 anemia.   (2) Non-hematologic toxicity:   - Class 4 non-hematologic toxicity; - Grade 3 non-hematologic toxicity that does not recover to grade ≤2 within 3 days after treatment (except for simple laboratory test abnormalities that, in the judgement of the investigator, are not clinically symptomatic and do not require intervention).   (3) Other toxic reactions that the investigator judged to have to permanently discontinuation of the drug or to have caused discontinuation of the first cycle for more than 7 days.  The above adverse events were graded according to the American Institute for Cancer Research's Routine Toxicity Criteria (NCI CTCAE) version 5.0.  The DLT evaluates cases in at least 75% of participants who have reached the planned dose administered. |
| **security**  **assessment** | Adverse events (AEs), serious adverse events (SAEs), suspected and unexpected serious adverse reactions (SUSAR), laboratory tests, vital signs, physical examination, 12-lead ECG abnormalities, etc. (CTCAE 5.0 criteria). |
| **Efficacy evaluation** | Adoption of the RECIST 1.1 standard |
| **PK research** | **Dose-escalation Phase - QD administration**: Subjects will first undergo a single-dose pharmacokinetic (PK) study, i.e., 1 dose followed by 7 days of observation, with blood collection points including before the first dose (within 0.5 h), 0.5, 1, 2, 4, 6, 8, 12, 24 (D2), 48 (D3), 72 (D4), 96 (D5), 120 (D6), 144 (D7) and 168 h (C1D1 before dosing). A PK study is then performed with once daily continuous dosing for 28 days per cycle. Blood collection points including pre-dose (within 0.5 hours), and 0.5, 1, 2, 4, 6, 8, 12, and 24 h post-dose on day 28 of cycle 1 (window period ±3).  **Dose-escalation Phase - BID administration**: Subjects will first undergo a single-dose pharmacokinetic (PK) study, i.e., 1 dose followed by 7 days of observation, with blood collection points including before the first dose (within 0.5 h), 0.5, 1, 2, 4, 6, 8, 12, 24 (D2), 48 (D3), 72 (D4), 96 (D5), 120 (D6), 144 (D7) and 168 h (C1D1 before dosing). A PK study is then performed with two daily continuous dosing for 28 days per cycle. Blood collection points including pre-dose (within 0.5 hours), and 0.5, 1, 2, 4, 6, 8, and12 h post-dose on day 28 of cycle 1 (window period ±3).  In the trial, the PK blood collection point can be adjusted according to the PK results.  **Dose-expansion phase**: Subjects will be directly enrolled in the PK study with twice-daily continuous dosing for 28 days per cycle. Blood collection points include days 1 and 28 of cycle 1 and 28 (window period is ±3 days), before (within 0.5 h), and 0.5, 1, 2, 4, 6, 8, and 12 hours after the first dose.  The PK blood collection point can be adjusted according to the PK results. |
| **Statistical methods** | The baseline data of this trial are analyzed by the full analysis set (FAS), and all validity indicators are analyzed according to the FAS and conformity protocol set (PPS); safety analysis is performed by the safety analysis set (SAS); PK analysis is performed by the PK Analysis Set (PKAS). |
| **End of study** | The end of this study is defined as the end of the last subject's second cycle of continuous medication for 28 days, disease progression, withdrawal of informed consent, loss to follow-up, or death (whichever occurs first). Participants who benefit from the study will continue to participate in long-term observational studies of efficacy and safety. |
| **Trial progress** | This study is expected to last 2 years. |

**List of acronyms**

| **abbreviation** | **Full name in English** |
| --- | --- |
| AE | Adverse event |
| WHITE | Album |
| ALP | Alkaline phosphatase |
| OLD | Alanine aminotransferase |
| Ames | Ames trial |
| ANC | Absolute neutrophil count |
| APTT | Activated partial thromboplastin time |
| AST | Aspartate aminotransferase |
| AUC | Area Under Curve |
| AUC_0-24_ | Area Under Curve0-24 |
| AUC_0-∞_ | Area Under Curve0-∞ |
| AUC_ss_ | steady-state Area Under Curve |
| BP | Blood pressure |
| GOOD | Blood urea nitrogen |
| CDISC | Clinical Data Interchange Standards Consortium |
| CK | Creatine kinase |
| CK-MB | Creatine kinase isoenzymes |
| CL | Plasma clearance |
| C_max_ | Maximum observed plasma concentration |
| C_max,ss_ | Maximum observed steady-state plasma concentration |
| CL/F | Bioavailability corrected clearance |
| CNS | Central Nervous System |
| Cre | creatinine |
| CR | Complete remission |
| CRF | Case report form |
| CT | Computed tomography |
| DBIL | Direct Bilirubin |
| DCR | Disease control rate |
| DDS | Dose-determining set |
| DLT | Dose-limiting toxicities |
| DR/DOR | Duration of response |
| EC | Ethics Committee |
| ECG | electrocardiogram |
| ECOG | Eastern cooperative oncology group |
| EIU | Pregnancy exposure/intrauterine exposure |
| MOTHER | European Medicines Agency |
| FAS | Full analysis set |
| FDA | Food and Drug Administration |
| FIB | Plasma fibrinogen |
| FISH | Fluorescence in situ hybridization |
| GCP | Good clinical practice |
| GIVE | Cholesterol |
| GLB | c-Globulin |
| GGT | Glutamyl transpeptidase |
| HBDH | Hydroxybutyrate dehydrogenase |
| HCC | Hepatocellular Carcinoma |
| HDL | High-density lipoprotein |
| Hgb | Hemoglobin |
| HR | Heart rate |
| IBIL | Indirect bilirubin |
| ICH | International Conference on Harmonization of Technical Requirements for Registration of Pharmaceuticals for Human Use |
| IEC | Independent ethics committee |
| IHC | immunohistochemistry |
| INR | International normalized ratio |
| IRB | Institutional Review Board |
| HERE | Intention-to-treat |
| MRI | Magnetic resonance imaging |
| MRSD | Maximum recommended starting dose |
| MTD | Maximum tolerated dose |
| Mono | Monocytes |
| NCI-CTCAE | National Cancer Institute Common Terminology Criteria Adverse Events |
| NGS | Next generation sequence |
| NMPA | National Medical Products Administration |
| NOAEL | No observed adverse event level |
| NSCLC | Non-small cell lung carcinoma |
| ORR | Objective response rate |
| THE | Overall survival |
| PKAS | PK analysis set |
| PCR | Polymerase chain reaction |
| PD | Progressive Disease |
| PD | pharmacodynamics |
| ON | Physical examination |
| PET | Positron Emission computed tmography |
| PFS | Progression-Free Survival |
| PgP | P - glycop rotein |
| HP | Pharmacokinetics |
| PMDA | Pharmaceuticals and Medical Devices Agency |
| PPS | Perprotocol set |
| PR | Partial Remission |
| QTc | QT interval corrected for heart rate |
| Rac | Accumulation index |
| RBC | Red blood cell |
| RD | Recommended Dose |
| RT-PCR | Reverse Transcription-Polymerase Chain Reaction |
| SAE | Serious Adverse Event |
| SAS | Safety analysis set |
| SD | Stable Disease |
| SOP | Standard Operating Procedure |
| T_1/2_ | Terminal half-life |
| TBIL | Total bilirubin |
| TEAEs | Treatment-emergent adverse events |
| TGI | Tumor growth inhibition |
| T_max_ | Time to maximum observed plasma concentration |
| TTP | Time to progression |
| ULN | Upper limit of normal |
| CEO/F | Bioavailability corrected apparent volume of distribution |
| WBC | White blood cell |

# 1. Research background

## 1.1 Introduction to the *RET* gene

**1.1.1 RET Biology**

The *RET* proto-oncogene was first identified in 1985 by transfection of NIH3T3 cells with human lymphoma DNA ^[1].^  The RET proto-oncogene is localized on chromosome 10q11.2, with a total DNA length of 60 kb, contains 21 exons, and encodes the RET protein composed of 1100 amino acids, which is a tyrosine kinase receptor containing an extracellular cysteine region, a transmembrane region, an intracellular region and a catalytic tyrosine kinase activity ^[2]^. RET proteins bind to ligand-co-receptor complexes of glial cell-derived neurotrophic factor (GDNF) family ligands (GFLs) to form a binary complex that mediates RET dimerization and activation ^[3].^ RET is involved in cell proliferation, nerve conduction, cell migration, and cell differentiation, and activates various downstream pathways through ligand/complex receptor/RET complex singaling, such as RAS/RAF/MEK/ERK, PI3K/AKT and STAT pathways to induce cell proliferation ^[4].^ During embryogenesis, this kinase plays an important role in the development of the renal and enteric nervous systems ^[5-7].^ RET is also important for homeostasis in a variety of tissue types, including neural, neuroendocrine, hematopoietic, and male germ cell tissues ^[8].^ Mutations in this ligand-receptor system can lead to intestinal ganglionopathy with congenital megacolon (Hirschsprung’s disease) and congenital renal and urinary defects ^[9].^ In addition, RET is also expressed in neural tumors, such as medullary thyroid cancer (MTC), pheochromocytoma (pheochromocytoma), neuroblastoma and so on ^[10].^ Activating mutations and genetic recombination of RET have also been found in the development of a variety of other tumors, including breast, prostate, lung, brain and pancreatic cancers.

**1.1.2 Carcinogenic Activation of RET**

**Mechanism of oncogenic activation of RET**

Oncogenic activation of RET can occur through two main mechanisms: First, chromosomal rearrangements can generate hybrid protein that fuse the RET kinase domain with chaperone proteins containing the dimerized domain ^[11-14].^ Second, mutations can directly or indirectly activate kinases ^[15-17].^ These somatic or germline alterations are implicated in the pathogenesis of several cancers ^[11-18].^ More importantly, activating RET rearrangements and mutations share common characteristics with oncogenes of identified targets.

RET-mediated carcinogenesis may involve mechanisms other than RET rearrangements and mutations. First, increased RET expression in the absence of defined genomic alterations may contribute to the growth and survival of certain cancers. Second, RET was identified as a strong negative regulator of major histocompatibility complex (MHC) class I expression in a variety of histological cancer cell lines, suggesting a possible role of RET inhibition in upregulating the anticancer immune response ^[19].^

**RET rearrangement**

The RET chromosome rearrangement involves the long arm of chromosome 10 and results in the fusion of the 3′ kinase domain coding region of RET with various 5′ heterologous upstream chaperone genes ^[12-14].^ RET rearrangement can lead to downstream pathway activation through two main mechanisms. First, chimeric gene fusion can lead to transcriptional control of RET kinases through their partner genes ^[12,20].^ Notably, RET expression is usually restricted to specific non-embryonic cell types, while its chaperone genes can be expressed everywhere. Thus, RET rearrangement status can lead to increased RET expression, the level of which may affect its oncogenic potential ^[21].^ Second, upstream fusion partners can contribute dimerization domains, leading to ligand-independent dimerization and activation of RET kinases ^[12-14,22].^ RET rearrangement is more common in radiation-induced cancers or radiation-sensitive organs ^[23].^

**RET mutations**

The most common type of mutation in the RET gene is point mutation, which is most commonly found in exons 10 and 11, corresponding to the regulation of RET-rich cysteine domains, and these cysteine residues maintain the stability of the extracellular receptor protein structure through disulfide bonds. When mutations occur, the receptor protein conformation changes and the disulfide bond between the two receptor proteins bind to each other, causing the downstream signaling pathways to activate automatically without ligands or stimulation ^[18].^ Exons 13, 14, 15, and 16 are part of the structural domain that regulate the intracellular RET tyrosine kinase; others occur in other parts of the RET gene, such as exons 3, 5, and 8.

Since the initial discovery of RET mutations causing multiple endocrine neoplasia type 2 (MEN2), a total of 72 different mutation points have been identified, of which 50 are located in 7 common exons (exons 8, 10, 11, 13-16). These mutations vary in clinical phenotype and risk classification ^[24].^

## 1.2 RET and Solid Tumors

**1.2.1 RET and Lung Cancer**

Lung cancer is the leading cause of morbidity and mortality in the vast majority of countries worldwide. It is estimated that in 2018, there were 2.094 million new lung cancer patients and 1.761 million deaths worldwide, accounting for 11.6% and 18.4% of all cancer incidence and death, respectively ^[25]^. RET fusion occurs in 1-2% of non-small cell lung cancers (NSCLCs) in the population, and RET mutations and fusions are very rare in small cell lung cancer ^[26].^ However, due to the large population base of lung cancer, this type of patients is not uncommon in clinical practice. In recent years, the incidence of lung cancer in China has been increasing year by year, and the age of onset is younger. A number of studies have summarized the relatively obvious clinical features of NSCLC patients with RET gene fusion mutations, generally more common in patients with low age (≤ 60 years) and non-smoking patients, and most patients have clinical features of low differentiation tendency in pathology, tumor tumors are small but often multiple, and lymph node metastases up to N2 or above ^[12,13,27-34].^

The most common RET fusions in NSCLC are KIF5B-RET and CCDC6-RET. KIF5B is the most common RET fusion partner in NSCLC, is detected in up to 70–90% of cases ^[35].^ The breakpoint cluster region in KIF5B may occur in multiple introns, the most commonly intron 15 ^[36].^ The detection rate of RET fusions CCDC6-RET is 10–25%, and others such as NCOA4-RET, TRIM33-RET, ZNF477P-RET, ERCC1-RET, HTR4-RET, CLIP1-RET, are about 18% ^[37-40].^

Since their discovery, RET fusion genes have been thought to be mutually exclusive with other mutated genes, but a growing number of studies ^[41,42]^ have confirmed that RET fusion genes can co-exist with genetic variants such as EGFR, MAP2K1, CTNNB1, AKT1, TP53, SETD2, and MET amplification. LU et al. ^[42]^ also noted that patients with RET gene fusion NSCLC with TP53 mutations had shorter OS. In addition, it has been suggested that RET gene fusion may be one of the mechanisms leading to first ^[43]^ and third-generation (Osimertinib ^[44]^) EGFR-TIKs acquired resistance.

**1.2.2 RET and Thyroid Cancer**

According to Cancer statistics 2018, the incidence and mortality of thyroid cancer ranked 9th and 6th in the world, respectively. In 2018, there were 567,000 new cases of thyroid cancer and 41,000 deaths worldwide, accounting for 3.1% and 0.4% of all cancer morbidity and death, respectively. The incidence in women (10.2 per 100,000) is more than 3 times that of men (3.1 per 100,000). Moreover, data show that thyroid cancer is one of the fastest growing malignant tumors in China, and it is the malignant tumor with the highest incidence rate in the female population, ranking 6th in the incidence of female malignant tumors ^[25].^ The main histological types of thyroid cancer are papillary carcinoma (PTC), accounting for more than 80%, followed by follicular carcinoma (FTC), the former two are collectively called differentiated thyroid cancer (DTC), less common types are medullary carcinoma (MTC), poorly differentiated carcinoma (PDTC), and undifferentiated carcinoma (ATC).

The occurrence and development of thyroid cancer is the result of polygenic regulation, and transcriptional and post-transcriptional regulation play a dominant role, mainly involving mitogen-activated protein kinase (MAPK) pathway and phosphoinositol-3-kinase (PI3K)/AKT signaling pathway activation. Among them, BRAF, TERT, RAS, RET, PAX8/PPARγ and TP53 genes are closely correlated with thyroid cancer diagnosis, aggressiveness, targeted therapy and prognosis ^[45].^

**RET and PTC**

There are at least 13 different RET fusions in PTC, the two most common of which are CCDC6-RET (also known as RET/PTC1) and NCOA4-RET (also known as RET/PTC3), accounting for 90% of all rearrangements ^[46-51]^. Other RET fusions found in PTC include RET/PTC2, RET/PTC4 via RET/PTC9, ELKS-RET, PCM1-RET, RFP-RET and HOOK3-RET ^[50].^ A parallel sequencing study targeting 244 cancer-associated genes and 20 fusion genes showed that RET fusion was detected in 4.35% of papillary thyroid carcinoma (PTC) ^[52].^ A comprehensive multi-platform analysis by the Cancer Genome Atlas (TCGA) network showed that RET fusion in 6.8% of PTC ^[53].^

Other studies have shown that somatic RET gene rearrangements account for 2.5-73% of sporadic PTCs, and RET fusion more prevalent in radiation-exposed populations. RET rearrangement is present in 84% of PTCs and 45% of follicular adenomas in patients with external thyroid irradiation, with RET/PTC1 being the most common, followed by RET/PTC3 ^[54]^. Among PTC patients exposed to radiation from the atomic bombings of Hiroshima or Nagasaki, 22% of PTC patients were found to have RET/PTC rearrangement. RET fusion occurs more frequently at 50% in patients with high-dose (> 0.5 Gy) exposure ^[55-56].^

**RET and MTC**

MTC is an endocrine tumor originating in parafollicular cells (C cells) of the thyroid gland, accounting for about 1%-5% of thyroid cancer, and is divided into sporadic medullary carcinoma (sMTC) and hereditary medullary carcinoma (hMTC) ^[57].^ RET gene mutations are the molecular basis for the pathogenesis of most MTCs, and RET mutations occur in 40% - 60 % of sMTCs and almost all hMTCs ^[58].^ Germline missense mutations are strongly associated with autosomal dominant multiple endocrine neoplasia type 2 (MEN2) syndromes, particularly MEN2A, MEN2B, and familial medullary thyroid cancer (FMTC) ^[59].^

**(1) RET and MEN2A**

MEN2A-associated RET mutations are most commonly found in regions encoding the highly conserved extracellular tyrosine-rich domain, most commonly in exons 10 and 11. In particular, codon 634 in exon 11 is the most common, accounting for approximately 85% of all MEN2A mutations. 634 mutation positivity is indicative of a more aggressive MTC, with a significantly increased incidence of paraneoplastic, pheochromocytoma, and cutaneous mossy lesions ^[60].^ MEN2A patients with Hirschsprung’s disease also have a high prevalence of RET mutations, with a high proportion at codons 620 and 609 ^[61].^ In addition, MEN2A patients also develop mutations in exons 5, 8, 13, 14, and 15, but these are rare.

FMTCs occur predominantly in exons 10 and 11, with mutations in exon 10 (codons 609, 611, 618, and 620) behaving broadly similarly to MEN2A-related mutations, so that FMTCs with such mutations represent a subtype of MEN2A ^[62].^ A common mutation in exon 11 is codon 634, which accounts for 30% of FMTCs. Codons 768, 790, and 791 of exon 13 and 804 of exon 14 are also common ^[63].^

**(2) RET and MEN2B**

MEN2B type only accounts for 5% of MEN2, but it has an early onset, rapid progression, high malignancy, and poor prognosis, which is very different from the clinical characteristics of MEN2A. More than 95% of MEN2B is caused by mutations in the codon 918 of exon 16. This mutation mainly affects the intracellular domain of RET, and has a strong tendency to early invasion and metastasis. M918T has the poorest prognosis of all mutations, and 95% of them are inherited from the germ cells to the offspring. The A883F mutation in exon 15 occurs in about 5% of cases ^[64]^, and MTC is less aggressive than M918T.

**(3) RET and sMTC**

RET mutations occur in In 30%-50% of sMTC patients, with mutation hotspots in exons 10, 11, and 16, and there is no significant difference in clinical features between sMTC and hMTC with the same mutation ^[65]^. The M918T mutation is the most common, similar to MEN2B, this sMTC is the most invasive with lymph node and distant metastases and a poor prognosis. However, the response to drugs is relatively good ^[66]^, and genetic testing is of great importance for the treatment and prognosis of sMTC.

**1.2.3 RET and Neuroblastoma**

In neuroblastoma (NB), the decrease in RET transcription precedes the decrease in RET protein. Studies have shown that when ALK is activated or inhibited in neuroblastoma cell lines, ETV5 is affected at both protein and mRNA levels, and this effect precedes its regulation by RET. Chip-seq analysis confirmed that ETV5 binds to the RET promoter and activates the enhancer upstream of the promoter, thereby driving the transcription of the *RET* gene. In a MYCN- and alkf1178l-driven mouse neuroblastoma model, the combination of RET inhibitor and ALK inhibitor inhibited tumor growth rate more effectively than either agent alone. These results suggest that the ERK-ETV5-RET pathway is key to activating neuroblastoma tumorigenesis downstream of ALK ^[67].^ RET is required for NB cell proliferation, so high RET expression may lead to a poor prognosis for NB patients. RET is a potential therapeutic target for NB, and the use of novel RET inhibitors may improve the patient prognosis ^[68]^.

**1.2.4 RET in Pheochromocytoma and Hirschsprung’s disease**

In a previous study, 25% of patients with pheochromocytoma or paraganglioma had missense or nonsense germline gene variants, including the 136C>T (p. R46X) variant of the c.SDHB gene, the c.1901G>A (p.C634Y) variant of the c. RET gene, the c. 2712C>G (p.S904S) variant and the c.2071G>A (p.G691S) variant of the c.RET gene. The first two variants have been shown to be pathogenic and are present in patients with pheochromocytoma and/or paraganglioma, so it is important to test the c.SDHB and RET genes in patients with pheochromocytoma and/or paraganglioma ^[69]^. Hirschsprung’s disease (HSCR) is a complex genetic disorder of the enteric nervous system, most commonly caused by the complete loss of neurons and ganglia in the enterocytes. More than 80% cases are caused by RET gene mutations. By immunofluorescence staining and PCR amplification of the RET gene, the generated iPSCs were found to express multiple markers of OCT4, SSEA4, SOX2, tra1-60 and NANOG. It was also found that hscrp-pscs-positive cells could be cultured in vitro and could be differentiated into tricellular cells, confirming that RET gene mutations can cause HSCR ^[70].^

**1.2.5 RET and Other Tumors**

With the exception of thyroid and lung cancer, RET fusion has not been extensively studied in solid tumors. There are reports of RET fusion protein expression in lung sarcoma (16.7%), ovarian epithelial carcinoma (1.9%), salivary adenocarcinoma (3.2%), pancreatic ductal carcinoma (0.6%), and unknown primary carcinoma (0.7%). RET/PTC3 has been detected in the human malignant pleural mesothelioma cell line EHMES-10 ^[71].^ RET rearrangements CCDC6-RET, NCOA4-RET, and KIF5B-RET were found in 0.2%-1.6% of colorectal cancer cases ^[72,73]^. RET rearrangements were detected in 0.16% of breast cancers, including CCDC6-RET, NCOA4-RET, and RASGEF1A-RET. In NIH/3T3 fibroblasts and MCF10A breast cells, the expression of NCOA4-RET and RASGEF1A-RET and the amplification of RET resulted in increased growth capacity and clonal expansion ^[74].^

## 1.3 RET Inhibitors

Over the past 20 years, a large number of small molecule tyrosine kinase inhibitors have been developed, which can cross the cell membrane and block the signalling pathway of cancer cell growth and division inside tumor cells. Abnormal activation of RET is a key driver of the growth and proliferation of various solid tumors such as non-small cell lung cancer (NSCLC), medullary thyroid cancer (MTC), and papillary thyroid carcinoma (PTC), and the research and development of anti-tumor drugs targeting RET has become a research hotspot at home and abroad. Trials of tyrosine kinase inhibitors targeting RET have mainly focused on thyroid cancer and NSCLC.

**1.3.1 Non-selective RET Inhibitors**

**Vandetanib**

On April 6, 2011, the FDA approved vandetanib tablets for the treatment of locally advanced or metastatic symptomatic or progressive MTC, becoming the first oral multi-target multi-kinase inhibitor approved by the FDA for MTC ^[75]^, whose targets are mainly RET, VEGFR-2 and VEGFR-3, produced by AstraZeneca pharmaceutical company in the United Kingdom, and which is currently not available in China.

Vandetanib is an aniline-based TKI originally developed as a vascular endothelial growth factor receptor (VEGFR) inhibitor ^[76]^. Vandetanib inhibits RET with an IC_50_ of 0.1 μmol/L, leading to inhibition of its downstream signalling pathway through inhibition of RET autophosphorylation as well as the oncogenic transformation ability of RET.

An international, multicenter, randomised, placebo-controlled, double-blind phase III trial of vandetanib in the treatment of MTC ^[77]^ included 331 patients with advanced MTC, and showed that median PFS in the trial group was approximately 11 months longer than in the control group (30.5 months vs. 19.3 months, HR: 0.46; 95% CI: 0.31- 0.69, P< 0.001); the disease control rate (DCR) was also 16% higher (87% vs. 71%) than in the control group; the PR rate of calcitonin and CEA (more than 50% lower than the baseline level) was significantly higher in the trial group than that in the control group (69% vs. 3%, 52% vs. 2%), and the time to worsening tumor pain was also significantly delayed in the trial group. Adverse effects with vandetanib were similar to those with other TKIs, the most common were diarrhea (57%) and hand-foot syndrome (53%). The incidence of grade 3 and 4 AEs was 57%, with diarrhea (11%), hypertension (9%), and QT interval prolongation (8%) being common. SAEs associated with vandetanib include QT interval prolongation, hypertension, interstitial lung disease, ischemic cardiovascular and cerebrovascular events, reversible leukoencephalopathy syndrome, and bleeding.

**Sorafenib**

Sorafenib was approved by the FDA in 2005 for the treatment of advanced renal cell carcinoma, and was approved for import and domestic use in 2006. In November 2013, the FDA formally approved sorafenib for the treatment of advanced iodine-refractory differentiated thyroid cancer, and in March 2017, sorafenib was approved for the indication of thyroid cancer in China. It is currently listed in the national medical insurance catalogue and belongs to the medical insurance category B.

Sorafenib is an inhibitor of a variety of kinases including Raf, VEGF2, VEGF3, PDGFR, FLT-3 and KIT ^[78]^. Sorafenib also inhibits RET activity and signalling, including ERK1/2, which blocks the growth of NIH3T3 cells transfected with the RET gene ^[79]^. The DECISION trial ^[80]^, a double-blind clinical trial in 416 patients with radioiodine (RAI)-refractory, locally recurrent or metastatic progressive differentiated thyroid cancer, showed that sorafenib improved median PFS by 5 months compared to placebo: 10.8 months vs. 5.8 months (HR=0.587, 95% CI, 0.454-0.758, P<0.0001); the PR rate in the trial group was also higher than in the control group (12.2% vs. 0.5%), with a median partial remission time of 10.2 months (95% CI, 7.4-16.6); the experimental data finally showed that there was no significant difference in OS between the two groups. However, 66% of patients receiving sorafenib discontinued dosing due to AEs. 14% of patients receiving sorafenib reported AEs leading to discontinuation, compared to 1.4% of patients receiving placebo. Grade 3 AEs were reported in 53% of patients receiving sorafenib, compared to 23% of patients treated with placebo. Grade 4 AEs were reported in 12% of patients receiving sorafenib compared with 7% of patients treated with placebo ^[81].^

**Cabozantinib**

Cabozantinib is an oral capsule developed by Exelixis Pharmaceuticals, Inc. in the United States, which has the advantages of high bioavailability, high blood-brain barrier permeability, and long clearance half-life ^[82]^, and can exert antitumor effects by inhibiting tyrosine kinase activities, such as RET, MET, VEGFR-1/2/3, and KIT ^[83-84]^. It was first approved by the FDA for the treatment of MTC in November 2012 ^[85]^ and was subsequently approved as second-line therapy in April 2016 and as first-line therapy in patients with advanced renal cell carcinoma (RCC) in December 2017 ^[86,87].^ It is not currently available in China.

Cabotinib is a potent inhibitor of RET, MET, and VEGFR-2 with a semi-inhibitory concentrations (IC_50_± SD) of (5.2±4.3), (1.3±1.2) and (0.035±0.01) nmol·L^-1 [88]^. The randomized, double-blind, placebo-controlled phase III clinical trial (NCT00704730) resulted in the approval of cabozantinib for MTC ^[89]^, 330 patients were randomised assigned (2:1) to cabozantinib (140 mg·d^-1^, n = 219) and placebo group (n = 111). The results showed that mPFS was 11.2 and 4.0 months with an HR of 0.28 (95% CI, 0.19-0.40, P<0.001) in the cabozantinib and placebo groups, respectively, and all subgroups (age, priors TKI treatment, and RET mutation type) showed a prolonged PFS; the ORRs were 28% and 0%, (P<0.001), respectively. Schlumberger et al. ^[89]^ further analyzed overall survival and showed that the mOS was 44.3 and 18.9 months in the RET M918T mutation-positive subgroup, cabozantinib group, and placebo group, respectively, with an HR of 0.60 (95% CI, 0.38-0.94, P=0.026). The most common AEs observed in this trial were diarrhea (63.1%), hand-foot syndrome (50.0%) and weight loss (47.7%). Due to these AEs, 16% of patients in the cabozantinib arm discontinued treatment. Overall, cabozantinib is highly toxic and has a high dose reduction rate, with approximately 69% of patients experiencing grade 3-4 AEs ^[90].^

**Sunitinib**

Sunitinib is developed by Pfizer, which was listed in China in 2007, and has entered the national medical insurance, with the following indications: 1. inoperable advanced renal cell carcinoma (RCC); 2. gastrointestinal stromal tumor (GIST) for which treatment with imatinib mesylate has failed or is intolerable; 3. unresectable metastatic highly differentiated advanced pancreatic neuroendocrine tumor (pNET) in adult patients.

Sunitinib is an oral multi-target TKI that inhibits a variety of RTKs and can inhibit tumor angiogenesis and anti-tumor through multiple receptors such as platelet-derived growth factor receptors (PDGFR, PDGFRα, PDGFRβ), vascular endothelial cell growth factor receptors (VEGFR1, VEGFR2, and VEGFR3). At present, the efficacy of sunitinib in the treatment of patients with NSCLC with RET gene fusion mutation is still controversial. In a phase II clinical study, sunitinib may improve the prognosis of advanced NSCLC ^[91].^ Wu et al. ^[92]^ reported a patient with KIF5B-RET fusion mutation-positive lung adenocarcinoma, who developed respiratory failure after oral sunitinib was given, and then the patient's respiratory distress symptoms were relieved, oxygenation status was significantly improved, and clinical treatment was successful, suggesting that sunitinib may have a certain therapeutic effect in patients with KIF5B-RET mutation-positive lung adenocarcinoma.

**Levantinib**

Levatinib is a novel targeted drug developed by Eisai in Japan, which was approved by the European Medicines Agency (EMA) and the FDA in 2015 for the treatment of radioactive iodine-refractory differentiated thyroid cancer. In December 2017, lenvatinib was granted priority approval and review qualification by the National Medical Products Administration of China (NMPA) for the treatment of liver cancer.

Levatinib is an oral multi-targeted RTK inhibitor that selectively inhibits the kinase activity of VEGFR as well as FGFR, PDGFRα, the tyrosine kinase KIT and RET. The phase II study (NCT01877083) included 25 patients with RET gene fusion mutation NSCLC who were treated with lenvatinib (24 mg/d). Results showed that 12 patients had tumor shrinkage with an ORR of 16% and DCR of 76%, and the mPFS was 7.3 months. This study also showed that 92% of patients had grade 3 or higher AEs, 64% of patients had treatment interruptions and 3 patients died due to AEs. The most common AEs were hypertension, nausea, decreased appetite, diarrhea, proteinuria, vomiting, with the incidence of the above being 68%, 60%, 52%, 52%, 48%, 44%, respectively ^[93]^.

**Alectinib**

In vitro studies have shown that alectinib inhibits fusion mutations in the RET, which is effective against two common RET fusion mutations (V804L and V804M); at the same time, alectinib does not inhibit VEGFR2, thus avoiding anti-angiogenic drug toxicity. In vitro studies have shown that alectinib inhibits the growth of KIF5B-RET-positive lung adenocarcinoma cells and induces the death of CCDC6-RET-positive lung adenocarcinomas cells ^[94].^

**Agerafenib（RXDX-105）**

RXDX-105 is a potent RET inhibitor developed by Ignyta, which is a multi-target inhibitor that can inhibit BRAF (V600E/WT) and C-RAF in tumor cells, as well as ABL-1, C-KIT, RET, PDGFRβ and VEGFR2. The NCT01877811 study included a total of 21 NSCLC patients NSCLC with a RET gene fusion mutation, 13 patients with KIF5B-RET positive, all patients had not received any targeted therapy prior to enrollment. 13 patients with KIF5B-RET fusion gene had no response to treatment, and 8 patients without KIF5B-RET fusion gene had an ORR of 75%; the most common AEs were rash, hypophosphatemia, elevated alanine aminotransferase (ALT), and anemia, and the incidence of the above AEs was 10%, 8%, 7%, and 7%, respectively ^[95].^

**1.3.2 Selective RET Inhibitors**

In addition to several non-selective RET inhibitors mentioned above, selective inhibitors targeting RET are also in development. Novel selective RET inhibitors such as BLU-667 and LOXO-292, with their good efficacy and low off-target toxicity, offer new hope for patients with RET drive gene positive tumours.

**LOXO-292（Selpercatinib）**

LOXO-292 is a potent selective RET inhibitor developed by Loxo Oncology, a subsidiary of Eli Lilly. Clinical studies of LIBRETTO-001 have shown good activity and tolerability of LOXO-292 in the treatment of RET fusion-positive tumors ^[96-97]^. After treatment with LOXO-292, the overall ORR in 105 RET fusion-positive NSCLC patients was 64%, of which 39 patients did not receive systemic therapy, resulting in an ORR of 84%. Of the 143 patients with thyroid cancer with RET mutation, 55 previously treated patients had an ORR of 69% and 88 patients who had not previously treated had an ORR of 73%. In 19 treated patients with RET fusion-positive thyroid cancer, the ORR was 79%. Based on this research, in May 2020, the US FDA approved LOXO-292 for the treatment of tumors with mutations or fusions of three RET genes—NSCLC, MTC, and other types of thyroid cancer. LOXO-292 also became the first selective RET inhibitor approved by the FDA.

**BLU-667(Pralsetinib)**

BLU-667 developed by Blueprint Pharmaceuticals, is a highly selective kinase inhibitor specifically designed to target RET, effectively inhibiting both wild-type RET kinase activity and carbotinib and vandetanib-resistant RET mutants (C634W, M918T, and V804L/M). Cellular studies showed that BLU-667 is significantly better than vandetanib, cabozantinib, and RXDX-105 at blocking MAPK signaling in RET-driven tumor cell lines, and its selectivity for RET is more than 10 times higher than other multi-target inhibitors, with only a weak inhibitory effect on VEGFR-2 ^[98]^.

On September 4, 2020, the US FDA accelerated the approval of the RET inhibitor Gavreto (pralsetinib) for the treatment of adult patients with RET fusion-positive NSCLC based on data from clinical phase I/II clinical trial (NCT03037385) ARROW ^[101]^. The study evaluated 42 patients with RET fusion-positive NSCLC with an ORR of 61%, tumor shrinkage observed in 95% of patients, and complete response in 14% of patients. In patients with RET-mutant MTC, the ORR was 60%, in patients previously treated with vandetanib, and tumor shrinkage was observed in approximately 98% of patients. The ORR in treatment-naïve patients MTC was 74%, and tumor shrinkage was observed in 100% of patients. In terms of safety, compared with multi-kinase inhibitors, the frequency and severity of vascular toxicities such as hypertension associated with BLU-667 treatment were significantly reduced due to the reduction in off-target inhibition of VEGFR2. The most common AEs were fatigue, constipation, musculoskeletal pain and increased blood pressure.

Based on the results of the ARROW Phase 1/2 clinical trial, Blueprint Medicines, Inc. and Genentech, Inc. recently announced that pralsetinib has received accelerated approval from the U.S. FDA for an expanded indication for the treatment of patients with RET-mutated thyroid cancer, including patients 12 years of age or older with advanced or metastatic RET-mutated medullary thyroid cancer (MTC) who require systemic therapy, or patients with radioiodine-refractory advanced or metastatic RET fusion-positive thyroid cancer who require systemic therapy.

**Other selective RET inhibitors**

Boston Pharmaceuticals' RET inhibitor BOS172738 is currently in phase I clinical trials (NCT03780517) in advanced RET fusion-positive NSCLC, advanced RET-mutated MTC and advanced solid tumours with RET gene alteration. GlaxoSmithKline's GSK3352589 and GSK3179106 are selective inhibitors of RET that have completed phase I clinical trials in irritable bowel syndrome, with potential applications in MEN2 and other RET-positive malignancies ^[99-100]^.

## 1.4 SY-5007

SY-5007 is a highly active small molecule RET tyrosine kinase selective inhibitor, developed by Shouyao Holdings (Beijing) Co., Ltd. (hereinafter referred to as " Shouyao Holdings "), which is a typical ATP competitive kinase inhibitor.

SY-4789 Chemical Name: 6-(3-hydroxy-3-methylazetidin-1-yl)-4-(6-(6-((6-methoxypyridin-3-yl)methyl)-3,6-diazabicyclo[3.1.1]heptan-3-yl)pyridin-3-yl)pyrazolo[1,5-a]pyridine-3-carbonitrile.

Molecular Formula: C_29_H_30_N_8_O_2_

Molecular weight: 522.61

All non-clinical pharmacology, pharmacokinetics, and toxicological studies of SY-5007 are conducted in accordance with GLP regulations, and relevant reports are filed by sponsors.

### 1.4.1 Pharmacology

The pharmacological studies of SY-5007 are mainly performed at three levels, including in vitro enzymatic level, cellular level, and in vivo in animals. The pharmacological properties of SY-5007 as a highly selective small-molecule RET inhibitor were studied through the target, mechanism of action, in vitro and in vivo pharmacodynamics and pathological detection of SY-5007. Detailed pharmacological findings are provided in the investigator's manual and the following conclusions can be drawn:

SY-5007 is a highly selective and potent small molecule RET tyrosine kinase inhibitor, a typical ATP-competitive inhibitor that inhibits kinase activity by competing with ATP for binding to a catalytic domain on protein kinases. Enzymatic assays showed that SY-5007 has very potent inhibitory activity against wild-type RET kinase (IC_50_: 0.339 nM) and significant selectivity for VEGFR2 (more than 10-fold, comparable to the control drug LOXO-292). SY-5007 also showed very high inhibitory activity against two key oncogenic mutations (V804M and M918T) with IC_50_ of 0.546 and 0.840 nM, respectively, and significant inhibitory activity against a key drug-resistant mutant (G810S) with an inhibitory IC_50_ of 2.07 nM. In the kinase selectivity assay, SY-5007 was found to have high selectivity for inhibiting RET kinase, and the selective analysis of nearly 100 kinases showed that SY-5007 had significant inhibitory effect on only a few kinases, indicating that SY-5007 is a highly selective and highly active small molecule RET inhibitor.

At the cellular level, SY-5007 is active against a panel of RET-positive (including various fusion forms, various mutant forms) tumour cells (TT, BaF3- CCDC6-RET ^WT, V804M, M918T^ ,293T- KIF5B-RET ^WT, V804M, M918T^) has a significant anti-proliferative effect, with IC_50_ values between 5-100 nM. For the RET-negative murine fibroblast line NIH-3T3 cells, the growth inhibitory activity is very weak, IC _50_> 5000 nM, indicating that SY-5007 also has high selectivity for RET protein at the cellular level. In cell function studies, SY-5007 was found to have a typical concentration-dependent inhibitory effect on RET signaling pathways in multiple RET-positive tumor cells, and in BaF3-CCDC6-RET^WT^ cells, very low concentrations of SY-5007 (1 nM) can significantly inhibit phosphorylation of RET, and can significantly block phosphorylation conduction of RET downstream proteins such as SHC and ERK, under the action of 10 nM, the effect basically reaches saturation. In the two mutant cells BaF3- CCDC6-RET^V804M/M918T^, SY-5007 also showed a strong blocking effect on the RET signaling pathway, showing a significant inhibitory effect at a concentration of 10 nM and saturation at 100 nM. A similar phenomenon was observed in another fusion form of tumor cells (293T-KIF5B-RET ^WT, V804M, M918T^), which means that at the cellular level, SY-5007 can inhibit the growth of RET tumor cells by inhibiting RET signaling pathway, whether it is RET wild-type or mutant tumor cells.

In animal models, SY-5007 significantly inhibited the xenograft tumor growth of RET-positive tumor cells BaF3-CCDC6-RET^WT, V804M, M918T^ and 293T-KIF5B-RET^V804M^ in a dose-dependent manner. In the BaF3-CCDC6-RET^WT^ xenograft tumor model, it was found that after 13 days of twice-daily administration, the tumor growth inhibition rate (TGI) of 3 mg/kg, 6 mg/kg and 12 mg/kg dose groups of SY-5007 was 59.2%, 80.1% and 93.5%, respectively, and the minimum 3 mg/kg could achieve a clear tumor growth inhibitory effect. Under the condition of high-dose administration conditions, the TGI of 40 mg/kg, 20 mg/kg and 10 mg/kg dose groups of SY-5007 was 93.0%, 107% and 112%, respectively, and the tumor volume was reduced and showed obvious regression, the tumor regression rate (REG) was 0%, 57.7% and 97.3%, respectively. Under parallel conditions, the antitumor activity of SY-5007 was superior to that of LOXO-292. SY-5007 also had a dose-dependent inhibitory effect on tumor growth in two xenograft tumor models with mutant BaF3-CCDC6-RET^V804M/M918T^. In the BaF3-CCDC6-RET^M918T^ tumor model, after 9 days of oral administration of SY-5007, the TGI of 10 mg/kg, 20 mg/kg and 40 mg/kg dose groups was 12.4%, 40.9% and 93.0%, respectively, and the antitumor activity of SY-5007 was superior to that of LOXO-292 under parallel conditions. In the BaF3-CCDC6-RET^V804M^ tumor model, after 9 days of oral administration of SY-5007, the TGI of 10 mg/kg, 20 mg/kg and 40 mg/kg dose groups was 35.4%, 85.3% and 109%, respectively. SY-5007 also exhibited dose-dependent tumor growth inhibition in a xenograft tumor model of 293T-KIF5B-RET^V804M^. After 14 days of oral administration of SY-5007 14 twice daily, the TGI of 10 mg/kg, 20 mg/kg and 40 mg/kg dose groups was 40.7%, 70.3%, and 94.1%, respectively. The antitumor activity of SY-5007 was significantly better than that of LOXO-292 (TGI 45.2%) under parallel conditions (20 mg/kg). PK/PD studies found that SY-5007 had significant concentration-dependent and time-dependent effects on RET signaling pathways in tumor tissues, and found that tumor growth inhibition was positively correlated with SY-5007 concentration in plasma and the inhibition of RET signaling pathways in tumor tissues. In addition, pathological evidence was also obtained in three mouse tumour models transplanted with BaF3-CCDC6-RET^WT/V804M/M918T^, and it was found that after oral administration of SY-5007, the tumor burden in several important organs such as spleen, liver and lung of tumor-bearing mice was significantly reduced, and the involvement of tumor cells in these organs was also greatly reduced, indicating that SY-5007 can not only inhibit the growth of tumors. It can also effectively reduce the metastasis of tumor cells to these organs, ultimately improving the quality of life of tumor-bearing mice.

**In vitro hERG potassium channel inhibition test**

The rapidly activating potassium channel encoded by the human *ether a-go-go-related gene* (hERG) is an important ion channel involved in the formation of phase 3 repolarisation of the myocardial action potential. Pharmacological blockade of hERG channels can lead to prolonged cardiac repolarisation with the electrocardiographic manifestation of a prolonged QT interval, known as long QT interval syndrome. Drug-induced delayed ventricular repolarisation can in some cases lead to a fatal arrhythmia, tip-twist ventricular tachycardia. To initially assess the preclinical cardiotoxicity of SY-5007, the inhibitory effect of SY-5007 on hERG potassium channels was investigated using the whole-cell membrane clamp technique (the gold standard for hERG safety assessment) and the risk of inducing ventricular repolarisation toxicity was assessed. The IC_50_ value of the test compound SY-5007 acting on hERG currents in the tested concentration range was 3.3 µM ± 368.5 nM, and SY-5007 was judged to have a moderate inhibitory effect on hERG channels according to the universal hERG standard. However, the in vitro GLP hERG assay IC_50_ value for the reference compound LOXO-292 in the literature was 1.1 µM. Therefore, although SY-5007 has some inhibitory effect on hERG channels, it may be safer for the heart than the reference compound.

**Cardiovascular function test in a beagle**

In the safety pharmacological study of cardiovascular effects, four male and four female beagles with buried implants were selected. The solvent (0.5% w/v MC dissolved in deionised water) and three different doses of the test drug SY-5007 (2, 4, and 6 mg/kg in males and 4, 8, and 12 mg/kg in females) were administered orally by the Latin formula, with a 3-day washout period between each dose. The animals were evaluated by cage-side and detailed clinical observations, electrocardiogram, heart rate, body temperature and blood pressure. No mortality was observed in male beagles at 2, 4 and 6 mg/kg of SY-5007 and in female beagles at 4, 8 and 12 mg/kg of SY-5007, and no correlation was found with any of the cardiovascular parameters evaluated, including systolic blood pressure, diastolic blood pressure, mean arterial pressure, heart rate, RR intervals, PR intervals, QRS intervals, QT and corrected QT intervals, and body temperature. None of the changes were found to be related to the study drug. This indicates that SY-5007 is safe for the cardiovascular system of beagle dogs.

**Rats’ central nervous system function test**

In the safety pharmacology study for CNS effects, 64 SD rats were used and randomly divided into 4 groups (8 males and 8 females in each group), males received a single oral administration of the solvent (0. 5% w/v MC in deionised water) and SY-5007 formulations at doses of 15, 30 and 40 mg/kg, and females received a single oral administration of the solvent and 30, 60 and 80 mg/kg of SY-5007 formulations, and females received a single oral administration of the solvent and 30, 60 and 80 mg/kg of SY-5007 formulations. All rats were evaluated for cage-side observation, detailed clinical observation, body weight and neurofunctional behavioural indices including in-cage observation, restraint observation, field observation, stimulus reflex observation and body temperature. The results showed that a single oral administration of SY-5007 at 15, 30 and 40 mg/kg in male SD rats and 30, 60 and 80 mg/kg in female SD rats did not cause any abnormalities in clinical observations, abnormalities in body weights and changes in body weights associated with the exposure, and did not affect any of the assessed neurofunctional-behavioural parameters of central nervous function. This indicates that SY-5007 is safe for the central nervous system of SD rats.

**Rats’ central respiratory system function test**

In the safety pharmacology study for respiratory effects, a total of 64 SD rats were used and randomly divided into 4 groups (8 males and 8 females in each group), with males receiving a single oral dose of the solvent (0. 5% w/v MC dissolved in deionised water) and SY-5007 formulations at doses of 15, 30 and 40 mg/kg and females received a single oral dose of solvent and doses of 30, 60 and 80 mg/kg of SY-5007 formulations. All rats were evaluated by cage-side observation, detailed clinical observation, body weight and respiratory parameters (tidal volume, minute tidal volume and respiratory rate). The results showed that a single oral administration of SY-5007 at 15, 30 and 40 mg/kg to male SD rats and 30, 60 and 80 mg/kg to female SD rats did not show any abnormalities in the clinical observations associated with the study drug, nor did it show any effects on the respiratory parameters of the rats associated with the study drug. This indicates that SY-5007 is safe for the respiratory nervous system of SD rats.

### 1.4.2 Pharmacokinetics

Non-clinical pharmacokinetic studies of SY-5007 included in vivo studies in rats and dogs and in vitro studies in rats, mice, dogs, monkeys and humans. Absorption, protein binding, tissue distribution, metabolism and excretion were assessed and basic pharmacokinetic parameters were obtained; the inhibitory and inducible effects of SY-5007 on key CYP450 enzymes were evaluated; the stability of in vitro hepatic microsomal metabolism and species differences in metabolic processes were assessed; the enzymatic phenotypes of key metabolic pathways were identified; and the permeability of SY-5007 was evaluated using a Caco-2 cell model (Table 1-1).

**Table 1-1 Summary of the list of pharmacokinetic tests**

| **Experiment type** | **Experimental species** | **Method of administration** | **Testing institutions** |
| --- | --- | --- | --- |
| Pharmacokinetics with single administration | Rats | i.v. | Shouyao Holdings |
|  |  | Gastric gavage | Shouyao Holdings |
| Pharmacokinetics of multiple administrations | Rats | Gastric gavage | Shouyao Holdings |
| Pharmacokinetics with single administration | dog | i.v. | Kanglong Chemical (Beijing) Biotechnology Co., Ltd |
|  |  | Gastric gavage | Kanglong Chemical (Beijing) Biotechnology Co., Ltd |
| Pharmacokinetics of multiple administrations | dog | Gastric gavage | Kanglong Chemical (Beijing) Biotechnology Co., Ltd |
| Tissue distribution for single administration | Rats | Gastric gavage | Shouyao Holdings |
| Plasma protein binding rate | Rats, mice, dogs, monkeys, humans | In vitro | Shouyao Holdings |
| Identification of liver microsomal metabolites and species differences | Rats, mice, dogs, monkeys, humans | In vitro | Shouyao Holdings |
| Stability of liver microsomal metabolism | Rats, mice, dogs, monkeys, humans | In vitro | Shouyao Holdings |
| Bile metabolite identification | Rats | Gastric gavage | Shouyao Holdings |
| Metabolite plasma exposure | Rats | Gastric gavage | Shouyao Holdings |
| Cytochrome P450 enzyme phenotypic identification | person | In vitro | Shouyao Holdings |
| Excretion of feces, urine, and bile | Rats | Gastric gavage | Shouyao Holdings |
| Cytochrome P450 enzyme inhibition | person | In vitro | Shouyao Holdings |
| Cytochrome P450 enzyme induced | person | In vitro | Shouyao Holdings |
| Caco-2 cell permeability | person | In vitro | Shouyao Holdings |

After a single i.v. administration of SY-5007 1 mg/kg to SD rats, the steady-state apparent volume of distribution (Vss) of the prodrug in rats was 697 mL/kg, the elimination half-life (t_1/2_) was 3.34h, and the clearance rate (CL) was 205 mL/h/kg. Plasma exposures in females (AUC_0-48h_, AUC_0-INF_) and mean residence times (MRT_INF_obs_) were significantly lower and CL was significantly higher in males. After a single gavage administration of SY-5007 (5, 20 and 50 mg/kg) to fasted SD rats, the time to peak plasma concentration (T_max_) of SY-5007 was 0.25~2h; the peak concentration (C_max_) was 2186, 7146 and 12646 ng/ml, with a t_1/2_ of 3. 61-4.49h; the MRT_INF_obs_ was 4.92~6.96h, and the AUC_0~INF_ was 11867, 48078, and 111646h*ng/mL, respectively; the oral bioavailability (F) was 39.1-42.1%. Plasma drug exposure (C_max_ and AUC_0~INF_) increased with dose, and the growth of C_max_ was gradually saturated, but the growth of AUC_0~INF_ was basically linear, while the t_1/2_ and MRT_INF_obs_ both increased slowly, suggesting that the absorption and elimination of SY-5007 in rats may be gradually saturated at both doses in the range of 5~50mg/kg. In addition, there was no statistically significant difference in the main parameters of plasma pharmacokinetics between female and male rats administered SY-5007 (5, 20, 50 mg/kg) by single gavage. After administration of SY-5007 (20 mg/kg once daily) by gavage to SD rats for 7 consecutive days, there was no significant difference in the plasma pharmacokinetic parameters of SY-5007 between day 1 and day 7 in females; in males, the C_max_ of the blood concentration at steady state (day 7) was not significantly different from that on day 1, and the AUC_0-24h_ was 47. 3% higher than that on day 1, P<0.05, indicating that the plasma pharmacokinetic parameters of SY -5007 at 20 mg/kg administered by gavage for seven consecutive days have a certain tendency to accumulate in male rats.

After a single intravenous administration of SY-5007 1 mg/kg to beagles, the Vss of the prodrug in beagles was 1431 mL/kg, the t_1/2_ was 3.27 h and the CL was 398 mL/h/kg. C_max_ was significantly lower in females than in males and there were no significant sex differences in any of the other parameters. After a single gavage administration of SY-5007 (2.5, 5 and 10 mg/kg) to fasted beagles, the T_max_ of SY-5007 plasma concentration ranged from 0.5 to 2 h, the C_max_ was 758, 1626 and 1436 ng/ml, the t_1/2_ ranged from 4.81 to 7.53 h, the MRT_INF_obs_ ranged from 5.75 to 11.4 h, and AUC_0~INF_ was 3763, 9853, and 9026h*ng/mL, respectively; oral bioavailability was 56.6%, 74.1%, and 33.9%, respectively. C_max_ and AUC_0~INF_ increased and then remained unchanged with dose, and t_1/2_ and MRT_INF_obs_ increased slowly, suggesting that SY-5007 may be gradually absorbed and eliminated from the body of beagles in the range of doses of 2.5-10mg/kg. Absorption and elimination in the body may both gradually tend towards saturation. In addition, there was no statistically significant difference in plasma pharmacokinetic parameters between female and male beagles administered SY-5007 by gavage alone. After SY-5007 was administered to beagles by gavage for 7 consecutive days (5 mg/kg once daily), there was no significant difference in the main pharmacokinetic parameters of SY-5007 blood concentration between day 1 and day 7, suggesting that accumulation of SY-5007 at 5 mg/kg for 7 consecutive days is unlikely to occur in beagles.

After a single gavage administration of SY-5007 (20 mg/kg) to rats, SY-5007 was widely distributed in vivo. The in vivo exposures (AUC_0~24h_) of SY-5007 in rats were in the following order from high to low: liver, stomach, lung, colon, spleen, kidney, ovary, body fat, small intestine, heart, uterus, skeletal muscle, testis and brain. The exposures in body fat, small intestine, heart, uterus, skeletal muscle, testis and brain were lower than in plasma and the rest were higher than in plasma. The highest exposures were found in the liver, stomach and lung, which were 358, 202 and 173% of plasma, respectively. The lowest distribution was found in the rat brain, where only a small amount of SY-5007 was detectable and the exposure was only 0.195% of plasma. There was no significant sex difference in the exposure of SY-5007 in rat tissues. Using rapid equilibrium dialysis, the binding rates of SY-5007 to plasma proteins of different species were, in descending order: mouse, monkey, rat, dog and human, with high binding rates of 99.0%, 97.7%, 96.6%, 95.8% and 93.9%, respectively.

SY-5007 in vitro metabolite identification was incubated with 50 μM SY-5007 in rat, mouse, dog, monkey and human liver microsomal incubation systems for 1 h, and a total of 8 monophasic metabolites were found in 5 metabolic forms, namely M1-a, M1-b, M1-c, M2, M3-a, M3-b, M4 and M5. The production of major metabolites in human liver microsomes is closer to that in rats or dogs than in mice or monkeys. The amounts of major metabolites produced in human liver microsomes was closer to the amount produced in rat, dog, monkey and human liver microsomes compared to that of rats or dogs. In vitro metabolic stability studies were performed using 1 μM SY-5007 incubated and analysed in the above systems, and the metabolic half-time of SY-5007 in monkey, mouse, human, dog and rat liver microsomes were 57.9 min, 109 min, 166 min, 280 min and 384 min, respectively. For the bile collection experiment, 20 mg/kg SY-5007 was administered by gavage to bile duct-intubated rats and the bile was collected for identification and analysis. In addition to the same eight monophasic metabolites as in the in vitro study, five di-phasic metabolites, M6, M7, M8, M9 and M10, were detected in rat bile, but the peak areas of the di-phasic metabolites were much smaller than those of the monophasic metabolites. Analysis of plasma samples from rats 0-48 h after administration of SY-5007 20 mg/kg by gavage showed that SY-5007 was mainly present as prodrugs in rat plasma, with small amounts of the monophasic metabolites detectable, of which the highest plasma exposures (AUC_0-INF_) were found for M1-b and M3-b, but none of them exceeded 1% of SY-5007.

The results of chemical inhibition experiments showed that the inhibitors α-naphthoflavone (CYP1A2), quercetin (CYP2C8), sulfonamidophenylpyrazole (CYP2C9) and quinidine (CYP2D6) inhibited the production of one or more metabolites of M1~M5 by 16.0~65. 2% in human liver microsomes, whereas ticlopidine (CYP2C19) had no significant inhibitory effect (inhibition rate <15%) on the production of M1~M5 in human liver microsomes; ketoconazole (CYP3A4) inhibited the production of 36.8~96.2% of M1~M5 except M2. Ticlopidine (CYP2C19) did not significantly inhibit M1~M5 in human liver microsomes (inhibition rate <15%); ketoconazole (CYP3A4) inhibited the production of 36.8~96.2% of M1~M5 in human liver microsomes, except M2. Therefore, the metabolism of SY-5007 in the human liver microsomal incubation system was mainly mediated by CYP3A4, CYP1A2, 2C8, 2C9 and 2D6 were also involved in the production of some SY-5007 metabolites, and CYP2C19 was not significantly involved in the metabolite production. Experiments with humanized recombinant CYP450 enzymes showed that CYP3A4 was involved in the formation of all known SY-5007 metabolites (M1 to M5) and contributed ≥72.1% to the formation of each metabolite; CYP1A2 contributed 1.83-26.4% to the formation of M1-a, M1-b, M2 and M3-a; CYP2B6, CYP2C9, 2C19 and 2D6 all contributed ≤3.42% to the formation of one or more metabolites in M1~M3. Thus, the humanized recombinant CYP450 enzyme experiments indicated that CYP3A4 was the major type of metabolising enzyme of SY-5007, CYP1A2 was involved in some metabolite formation and the contribution of CYP2B6, 2C9, 2C19 and 2D6 to metabolite formation was very low.

In the SY-5007 excretion study, rats were given 20 mg/kg of non-radiolabelled SY-5007 by gavage, and feces, urine and bile were collected from 0 to 48 h post-administration, and SY-5007 and its metabolites in the samples were determined by LC-MS-MS, which showed that the amount of SY-5007 excreted via feces from 0 to 48 h was 61. 7% of the administered amount, of which 55.4% for the prodrug and 6.22% for the metabolite, and 1.13% for urinary excretion, of which 0.801% for the prodrug and 0.332% for the metabolite. The total fecal and urinary excretion of prodrug and metabolite was 62.8% of the administered dose. The amount of prodrug and metabolite in bile from 0 to 48 h in bile duct-intubated rats was 5.22% of the administered dose, of which 0.656% was prodrug and 4.57% was metabolite. The above results indicate that SY-5007 (20 mg/kg) was excreted in the feces mainly as prodrugs and metabolites after gavage administration to rats.

To explore the potential drug-drug interactions (DDIs) induced by SY-5007, we performed a series of in vitro studies. Co-incubation of SY-5007 with substrates of different CYP450 enzymes in mixed human liver microsomes and detection of the corresponding substrate metabolites showed that SY-5007 (0.5-25 μM) inhibited CYP2C8, CYP2C9 and CYP3A4 (IC_50_ of 9.75, 6.68 and 18.0 μM, respectively) and none of the other CYP450 enzyme isoforms CYP1A2, 2C19, 2D6 and 3A4 showed significant inhibition (IC_50_>25 μM). Induction of CYP450 enzymes by SY-5007 was investigated using hepatocytes from three donors and the results showed that SY-5007 (1, 5 and 50 μM) had no induction of CYP1A2 and 3A4 and that CYP2B6 was induced by concentrations above 5 μM in both donors (56. 8-81.4% of the positive control). Permeability studies in Caco-2 cells, SY-5007 was found to be a moderately permeable compound with a P_app_ ratio >2 on the basolateral (BL) and luminal (AP) sides, which may be a P-gp substrate.

### 1.4.3 Toxicology

The toxicological tests of SY-5007 were commissioned by Kanglong Chemical (Beijing) Biotechnology Co., Ltd. in accordance with GLP regulations. The purpose is to assist in the trial design of SY-5007's first clinical trial in humans. The experiments carried out and the main results are listed in Table 1-2.

**Table 1-2 Toxicological test content**

| **Experiment type** | **Route of administration** | **Animal species** |
| --- | --- | --- |
| Acute toxicity | Oral gavage | SD rats, beagles |
| Long-term toxicity | Oral gavage | SD rats, beagles |
| *In vitro* genotoxicity | In vitro | *Salmonella typhimurium* and *Escherichia coli*, Chinese hamster ovarian cells |
| Genotoxicity *in vivo* | Oral gavage | SD rats |

A single oral gavage dose of SY-5007 (males: 25, 100, 250 mg/kg; females: 50, 200, 500 mg/kg) to SD rats was well tolerated by all animals. Changes associated with SY-5007 included: loose stools and yellow anal staining in females in the 500 mg/kg dose group; decreased mean body-weight gain and food consumption in >100 mg/kg males and 500 mg/kg females; mild elevation of ALP in >25 mg/kg males and >200 mg/kg females; and increased BU and food consumption in >100 mg/kg males and >50 mg/kg females; mild elevation of BU and BUN/C in >100 mg/kg males and >50 mg/kg females. Therefore, the maximum tolerated dose (MTD) for acute toxicity testing of SY-5007 administered by single oral gavage to SD rats is 250 mg/kg for males and 500 mg/kg for females.

A single oral gavage dose of SY-5007 (50, 100 and 200 mg/kg) to beagles was well tolerated by all animals. Changes associated with SY-5007 included vomiting in 100 mg/kg animals and a transient decrease in body weight in 100 mg/kg males. Therefore, the maximum tolerated dose (MTD) for acute toxicity testing of SY-5007 administered by single oral gavage to beagles is 200 mg/kg.

SD rats were treated with SY-5007 (males: 15, 30 and 40 mg/kg, females 30, 60, and 80 mg/kg) by oral gavage once daily for 28 consecutive days; all animals in the low and intermediate dose groups survived until scheduled necropsy, whereas males and females in the high dose group appeared dead on day 29 (1/15 deaths) and 32 (1/15 deaths) of the recovery period and on day 28 of the dosing period (1/15 deaths and 54 days (2/15 deaths) of the recovery period, respectively). The mean food consumption of the males in the intermediate dose group and the females in the high dose group from day 22 to 28 was 28.0% to 43.3% lower than that of the control, and the body-weight gain from day 1 to 28 was 51.4% to 92.0% lower than that of the control. During the recovery period (days 29-56), the food consumption of animals in the six dose groups was 14.9%-59.5% lower than that of the control, accompanied by generally lower body-weight gains than the control, but the absolute body weights of the animals increased in all groups except the male and female high-dose groups. A higher incidence of tail curling, staining, back arching, coat roughness, and reverse coat erection was observed from day 15 of dosing until the end of recovery in animals in the male intermediate dose group and in animals in the male and female high dose groups. When SY-5007 was administered to rats by gavage for 28 consecutive days, the dose at which ≤10% of the animals developed severe toxicity (STD_10_) was 30 mg/kg for males and 60 mg/kg for females. The C_max_ and AUC_last_ of plasma SY-5007 at day 28 at the respective corresponding doses were 8165 ng/mL in males and 110511 h*ng/ mL and 14925 ng/mL and 121575 h*ng/mL in females, respectively.

Beagles were treated with SY-5007 (males: 2, 4, and 6 mg/kg; females: 4, 8, and 12 mg/kg) by gavage once daily for 28 consecutive days; all animals in the low and intermediate dose groups survived to scheduled necropsy, and dead or dying animals occurred in males and females in the high dose group on day 21 (1/5 dead) and day 11 (1/5 near death), respectively. Hair loss, discharge, lethargy, pain, scabbing, decreased activity, lethargy, swelling, and loose stools occurred in a small number of animals in the intermediate and high dose groups and were not considered serious adverse effects due to the low frequency of occurrence. During the administration period, neutrophils in the male high-dose group, lymphocytes, eosinophils in the female high-dose group, and mean platelet volume in the female mid-dose group were significantly decreased, and ALT, CK, CHO in the male high-dose group and AST in the female high-dose group were significantly increased. All of the above changes in the low and intermediate dose groups were completely reversed except for the liver-to-brain ratio in the low dose female group. It is now tentatively concluded that beagles can tolerate the respective low and medium doses of SY-5007 administered by gavage for 28 consecutive days, with the highest non-severe toxicity dose (HNSTD) being 4 mg/kg in males and 8 mg/kg in females.

The genotoxicity study of SY-5007 consisted of three tests: *Salmonella typhimurium* and *Escherichia coli* revertant mutation (Ames) test, in vitro Chinese hamster ovary cell chromosome aberration test and in vivo rat micronucleus test. The Ames test and Chinese hamster ovary in vitro chromosome aberration test were negative at the receiving dose; the bone marrow cell micronucleus test was positive when SY-5007 was administered at ≥100 mg/kg/day in male rats and ≥300 mg/kg/day in female rats. The bone marrow cell micronucleus test was positive in male rats at ≥100 mg/kg/day and in female rats at ≥300 mg/kg/day.

# 2. Research Objectives and Evaluation Indicators

**Table 2-1 Research objectives and evaluation indicators**

|  | **Objectives** | **Evaluation indicators** |
| --- | --- | --- |
| **Primary** | - The safety and tolerability of SY-5007 tablets was evaluated in Chinese patients with advanced solid tumors to determine the MTD, DLT and RP2D. | - Safety: including adverse events (AEs), serious adverse events (SAEs), suspected and unexpected serious adverse reactions (SUSAR), laboratory tests, vital signs, physical examination, 12-lead ECG, etc - Tolerability: rate of discontinuation and interruption - MTD - DLT - RP2D |
| **Secondary** | - To evaluate the pharmacokinetic (PK) profile of SY-5007 tablets in subjects with advanced solid tumors; - Preliminary evaluation of the efficacy of SY-5007 tablets in subjects with advanced solid tumors with RET gene fusion or mutation | - PK parameters, including but not limited to C_max_, C_trough_, AUC_0~24_, AUC_0~∞_, T_max_, t_1/2_, bioavailability-corrected clearance CL/F, bioavailability-corrected apparent volume of distributionVd/F and accumulation index Rac. - Efficacy measures: objective response rate (ORR), disease control rate (DCR), duration of response (DOR), and progression-free survival (PFS) |

#

# 3. Study Population

## 3.1 Subject Number Rules

After signing the informed consent form, the subject will be first given a screening number, which is named as follows: **S + study center number + study code + screening sequence number**. The study code for the dose ramp-up phase is 1 and the study code for the dose expansion phase is 2. The screening number for each study centre starts at 001 and increases sequentially. Sample screening number: |S||0|1||1|0|0||3|, indicating the third subject screened by the study center 01 in the dose-escalation phase.

Successful subjects will be given an enrollment number, which is named as **E + study center number + study code + enrollment sequence number**. The enrollment number for each site starts at 001 and increases sequentially. Example of an enrollment number: |E||0||4|2||0|0||3|, indicating the third subject successfully enrolled in the dose expansion phase at research center 04.

Subjects will be assigned numbers in strict order; if a subject withdraws from the trial, that subject's enrolment number can no longer be used and the withdrawn subject can no longer participate in this trial.

## 3.2 Inclusion Criteria

To be eligible for this study, subjects must meet all of the following inclusion criteria:

- 1. Age≥ 18 years old, male or female;
  2. Eastern Cooperative Oncology Group (ECOG) performance status score of 0-1;
  3. Estimated life expectancy >12 weeks;
  4. According to RECIST V1.1, subjects must have at least one assessable lesion in dose-escalation phase and one measurable lesion in dose-expansion phase;
  5. **Dose-escalation phase**: subjects with histologically or cytologically confirmed advanced solid tumors who have failed standard therapy, or for whom no standard treatment is available, or for whom standard treatment is not available at this stage (e.g., subject refuses standard therapy), and for whom there is a RET gene fusion or mutation;

**Dose-expansion phase:** subjects with histologically or cytologically confirmed advanced solid tumors who have failed standard therapy, or for whom no standard treatment is available, or for whom standard treatment is not appropriate at this stage, and who have RET gene fusion in NSCLC or RET mutation in MTC or RET-altered other advanced solid tumors;

- 1. Subjects must have adequate organ function, defined as follows:

**Liver function**:

- Without liver metastases, serum aspartate aminotransferase (AST) and serum alanine aminotransferase (ALT) ≤ 3 times the upper limit of normal (ULN); subjects with liver metastases or hepatocellular carcinoma (HCC), AST, ALT ≤ 5 times ULN, total serum bilirubin (TBIL) ≤ 1.5 times ULN.

**bone marrow function (no transfusions or hematopoietic stimulating factor therapy within 10 days prior to testing):**

- Neutrophil absolute value (ANC) ≥ 1.5×10^9^/L;
- Platelets (PLT) ≥75×10^9^/L;
- Hemoglobin (Hb) ≥ 85g/L.

**Kidney function**:

- Creatinine clearance ≥ 50 mL/min.

**Coagulation function**:

PT or INR≤1.5×ULN.

**Lipids**:

Cholesterol ≤ 500 mg/dL (12.92 mmol/L).

- 1. All women of childbearing age must have a negative serum pregnancy test within 7 days prior to the first dose, and male and female subjects of childbearing potential must agree to abstinence or use contraception throughout the study period and for at least 3 months after the last dose of drug;
  2. Willingness and ability to give informed consent and follow protocol procedures, and comply with follow-up visit requirements.

## 3.3 Exclusion Criteria

Subjects with any of the following are not eligible for entry into this study:

1. **Dose expansion phase:** subjects carry known major driver gene alterations other than RET genes, e.g., EGFR, ALK, ROS1, KRAS, etc.;
2. **Dose expansion phase:** previous use of selective RET inhibitors;
3. Received antitumor therapy such as chemotherapy, radiotherapy, biological therapy, endocrine therapy, immunotherapy and other antitumor therapy within 4 weeks prior to first dose, except for the following:

• Nitrosourea or mitomycin C within 6 weeks prior to first dose;

• Oral fluorouracil analogs and small molecule-targeted drugs 2 weeks prior to first dose or within 5 half-lives of the drug, whichever is longer;

• Traditional Chinese medicines with anti-tumor indications are within 2 weeks prior to the first dose.

1. Received other unlisted clinical trial drugs or treatments within 4 weeks prior to the first dose;
2. Major organ surgery (excluding puncture biopsy) or had significant trauma within 4 weeks prior to the first dose, or required elective surgery during the trial;
3. Adverse effects of previous antitumor therapy have not returned to a CTCAE 5.0 grade ≤ 1 (except for toxicities judged by investigator to pose no safety risk, such as alopecia, grade 2 peripheral neurotoxicity, etc.);
4. Central nervous system(CNS) metastases with clinical symptoms, or other evidence of uncontrolled CNS metastases or meningeal metastases in subjects who, in the judgement of the investigator, are not suitable for enrollment;
5. Participants with active uncontrolled systemic bacterial, viral or fungal infection despite optimal treatment (not required to screen for chronic disease);
6. Active hepatitis B (HBV-DNA≥2000 IU/mL), hepatitis C virus infection (HCV antibody positive), HIV antibody positive, active syphilis, and still uncontrolled after active treatment, and is judged by the investigator to be unsuitable for enrollment;
7. History of severe cardiovascular and cerebrovascular disease, including but not limited to:

• Severe cardiac rhythm or conduction abnormalities, such as ventricular arrhythmias requiring clinical intervention, degree II-III atrioventricular block, etc;

• At rest, mMean QT interval corrected using Fridericia's formula (QTcF)> 480ms;

• Acute coronary syndrome, congestive heart failure, aortic dissection, stroke, or other grade 3 or above cardiovascular and cerebrovascular events within 6 months prior to the first dose;

• New York Heart Association (NYHA) ≥ class II heart failure or left ventricular ejection fraction (LVEF) < 50%;

•High blood pressure remains uncontrolled despite aggressive antihypertensive therapy. Uncontrolled hypertension is defined as systolic blood pressure >185 mmHg and/or diastolic blood pressure >110 mmHg measured three times at least 10 minutes apart;

1. Used any CYP3A4 inhibitor or inducer within 14 days prior to the first dose e;
2. Inability to swallow the drug orally, or conditions that, in the judgment of the investigator, severely interfere with gastrointestinal absorption;
3. Subjects who, in the opinion of the investigator, have a history of other serious systemic diseases, or are otherwise reasons unsuitable for participation in this clinical study.

## 3.4 Screening Failure

Screening failure is considered if participants have signed the ICF but the investigator has not determined their eligibility for enrollment. For all screening failures, investigators will enter at least the subject number, demographic characteristics, and reason for the screening failure in the electronic case report form (CRF). The data will also be kept in the research documents of the research center. When the investigator determines that the screening failure is likely to benefit, a number needs to be reassigned when the subject is re-screened (only allowed once).

## 3.5 Exit Criteria

In accordance with GCP and ICH guidelines, all participants can withdraw from the study at any time, without discrimination, retaliation, or medical treatment. In addition, participants may be asked to withdraw from the study at any time if, in the judgment of the investigator, for any of the following reasons:

- Intolerable toxicity or due to tolerability events require a delay of treatment for more than 2 weeks
- Poor adherence to study protocols
- pregnancy
- Withdrawal of informed consent
- death
- Loss to follow-up
- Other (please elaborate)

Participants who withdraw from the trial for safety reasons should be followed until toxicity resolves or returns to screening levels.

## 3.6 Early Termination of the Trial

Trials may be terminated at the request of the sponsor, the investigator, or the regulatory authority, and any request by a party to terminate a trial must be timely and properly communicated to the other parties, and the trial may be terminated earlier by mutual agreement. If it is necessary to terminate or suspend the trial, the investigator should inform the Ethics Committee of the reasons for the termination or suspension in accordance with the relevant regulatory requirement(s).

Termination of the trial should be considered in the following events:

• Researchers found serious security issues;

• There were significant errors in the test protocol;

• Sponsor reasons;

• The administrative authority withdraws the test;

• The sponsor has the right to decide to terminate a trial at a research center if:

- The research center is unable to complete the agreed number of enrollment at the agreed time
- The research center is in serious violation of the GCP
- The research center is in serious breach of the Protocol

After the study is terminated, all trial-related records should be retained for future review**.**

# 4. Study Design

## 4.1 Overall Study Design

This is a of single-arm, open-label, dose-escalation, and dose-expansion phase I clinical trial.

**Dose-escalation**: Subjects with advanced solid tumors will be enrolled and it is planned that the first two dose groups will be accelerated titrated, one subject in each group, with the next group starting if there are no non-hematological adverse events of grade 2 or higher or hematological adverse events of grade 3 or higher that are definitely or potentially related to the investigational drug; if any of the above adverse events occur, the escalation pattern will have to be changed to a "3+3" escalation phase.

"3+3" escalation phase: 3 subjects were enrolled in each dose group; if no DLT occurred in 3 subjects, subsequent subjects would proceed to the next dose level; if two DLTs occurred in three subjects, enrolment in that dose group will be stopped and, at the discretion of the investigator, three additional subjects could be enrolled at the previous dose level; if one DLT occurred in 3 subjects, 3 additional subjects would be required at that dose level, and if no additional DLT occurred and the investigator confirmed that this dose is well tolerated by the subjects, the study could proceed to the next dose level, or if ≥ one DLT occurred in the next 3 subjects, the study would be terminated at that dose level.

If an MTD is observed at the first dose level, if an MTD is not observed at the highest dose level, or if there is a large range between dose levels, dose escalation is likely, including the addition of a dose level lower than the starting dose or the addition of an intermediate dose level between two dose levels. Dose increases will be made until ≥2/6 cases of DLT occur or non-linear drug emergence occurs in a dose group. If no protocol-specified DLTs occur, a decision will be made to continue the dose group in conjunction with non-clinical information and effective dose and pharmacokinetic parameters observed in humans. Dose-escalation will be discontinued if the dose of SY-5007 exceeds a reasonable dose (2000 mg) and the MTD remains undetermined. The recommended escalation dose levels are listed in Figure 4-2, but the actual escalation dose levels are not limited to those listed in the table.

If a subject withdrew from the study before completion of cycle 1 for reasons other than the study drug, a new subject will be substituted. Subjects must have received at least three-quarters of the treatment in Cycle 1 (should be dosed for ≥ 21 days in a 28-day cycle) to be considered for data validity.

**Dose-expansion**: When the MTD or/RP2D dose is achieved, subjects with advanced solid tumors harbouring RET gene variants were enrolled in either the MTD dose group or 1-2 dose groups below the MTD for efficacy expansion. The dose expansion will be divided into three cohorts: RET fusion-positive NSCLC, RET-mutant MTC and other advanced solid tumors with RET gene variants (fusion or mutation); 10-30 cases per cohort expansion will initially be followed for clinical efficacy. If enrolment is difficult in a cohort study, the sponsor may decide to proceed based on clinical progress without enrolling the full required sample size.


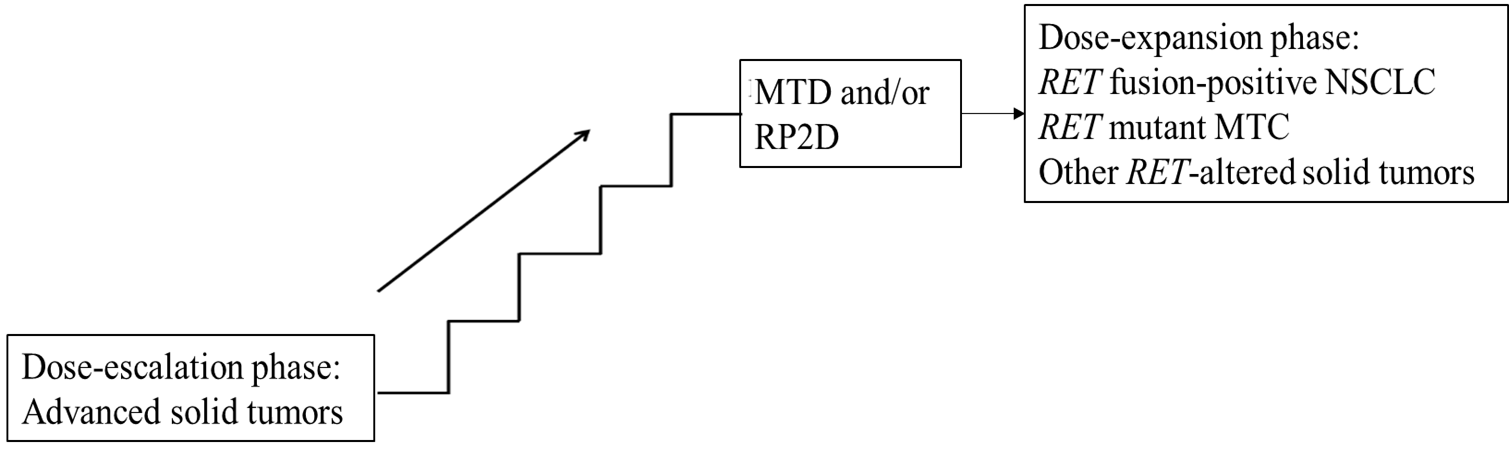


**Figure 4-1 Overall study design**

**Table 4-1 Proposed dose-escalation designs (including but not limited to the above dose groups)**

| **groups** | **Administered dose** | **Frequency of administration** | **Total daily dose** |
| --- | --- | --- | --- |
| 1 | 20mg | QD | 20mg |
| 2 | 20mg | BID | 40mg |
| 3 | 40mg |  | 80mg |
| 4 | 80mg |  | 160mg |
| 5 | 120mg |  | 240mg |
| 6 | 160mg |  | 320mg |
| 7 | 200mg |  | 400mg |

During the dose-escalation phase, the dose level after the initial dose is escalated in steps of 100%, 100%, 50%, 33% …. according to the Fischer's modified method until ≥2/6 of subjects developed DLT at a given dose, or until a dose group whose pharmacokinetic parameters indicate that absorption is saturated, then the dose-escalation is terminated.

Based on the safety, tolerability, PK characteristics and preliminary efficacy obtained from the study, the dosage and dosing regimen for subsequent clinical trial will be determined. If the above maximum dose still fails to reach MTD, then combine with preclinical data, effective dosage observed in human in domestic and foreign studies of similar drug, and PK parameters to determine the dose and dosage regime for subsequent clinical trials after a safety review by the investigator and medical supervisors.

## 4.2 Starting Dose

The starting dose of SY-5007 is determined based on a combination of its pharmacokinetics, pharmacodynamics, GLP toxicological/toxicokinetic studies, and clinical trials of homogeneous targets that have been approved for clinical use.

1) The in vivo efficacy model of SY-5007 showed that the minimum dose of 3 mg/kg BID could achieve a significant tumor growth inhibitory effect (TGI was 59.2%), the TGI reached 80.1% at the dose of 6 mg/kg BID, and the corresponding minimum effective dose of SY-5007 was 28.8 mg according to the surface area.

**Table 4-2 Converted from PK/PD effective dose + body surface area**

| **animal** | **Effective tumor suppressant dose (mg/kg)** | **HED(mg/kg)** | **MRSD(mg/kg)** | **MRSD(mg)^1^** |
| --- | --- | --- | --- | --- |
| **Tumor-bearing mice** | 6（3 mg/kg BID） | 0.48 | 0.48 | 28.8 |
| **Tumor-bearing mice** | 12（6 mg/kg BID） | 0.96 | 0.96 | 57.6 |

Note: 1. "Estimation of the Maximum Safe Starting Dose of the First Dose in Humans ", published by the US FDA in July 2005, translated and approved by the Drug Review Center in June 2009, assuming a human weight of 60kg.

2) According to the STD10 results of the SD rat long toxicity study (Table 4-3), the starting dose of human phase I clinical trial was calculated to be 28.8mg.

**Table 4-3 Conversion from GLP long-toxic STD10+ body surface area**

| **animal** | **STD10(mg/kg)** | **HED(mg/kg)** | **SF** | **MRSD(mg/kg)** | **MRSD(mg) ^1,2^** |
| --- | --- | --- | --- | --- | --- |
| **Male SD rats** | 30 | 4.8 | 10 | 0.48 | 28.8 |
| **Female SD rats** | 60 | 9.6 | 10 | 0.96 | 57.6 |

Note: 1. Non-clinical evaluation of antineoplastic drugs (published in ICH-S9 in 2009).

2. "Estimation of the Maximum Safe Starting Dose of the First Dose in Humans ", publishe by the US FDA in July 2005, translated and approved by the Drug Review Center in June 2009, assuming a human weight of 60kg.

3) According to the HNSTD toxicological study of the beagle dog (Table 4-4), the starting dose of human phase I clinical trial is calculated to be 21.6mg.

**Table 4-4 Converted from GLP long-toxic HNSTD+ body surface area**

| **animal** | **HNSTD (mg/kg)** | **HED(mg/kg)** | **SF** | **MRSD(mg/kg)** | **MRSD(mg)^1,2^** |
| --- | --- | --- | --- | --- | --- |
| **Male Beagle** | 4 | 2.16 | 6 | 0.36 | 21.6 |
| **Female Beagle** | 8 | 4.32 | 6 | 0.72 | 43.2 |

Note: 1. Non-clinical evaluation of antineoplastic drugs (published in ICH-S9 in 2009).

2."Estimation of the Maximum Safe Starting Dose of the First Dose in Humans ", published by the US FDA in July 2005, translated and approved by the Drug Review Center in June 2009, assuming a human weight of 60kg.

**Table 4-5 Calculation of maximum recommended starting dose**

| **Extrapolation method** | **Effective tumor inhibition dose +**  **Body surface area** | **Long poison STD10+**  **Body surface area** | **Long poison HNSTD+**  **Body surface area** |
| --- | --- | --- | --- |
| **Inferred dose (mg) ^1,2^** | 28.8 (mouse) | 28.8 (rats) | 21.6 (Beagle) |

Note: 1. Non-clinical evaluation of antineoplastic drugs (published in ICH-S9 in 2009).

1. "Estimation of the Maximum Safe Starting Dose of the First Dose in Humans", published by the US FDA in July 2005, translated and approved by the Drug Review Center in June 2009, assuming a human weight of 60kg.

According to the pharmacokinetic and pharmacodynamic characteristics of SY-5007, considering the subject's human safety and avoiding the subject's long-term exposure to an ineffective dose, 20 mg QD was used as the starting dose in this study.

## 4.3 DLT Definitions

According to the National Cancer Institute's Criteria for the Determination of Routine Toxicity (NCI CTCAE) version 5.0 grading scheme, DLT is defined as the following toxic reactions associated (both definitely associated and possibly associated) with the study drug, occurring within a single dose and within cycle 1 (35 days) of consecutive doses of the study drug:

(1) Hematological toxicity:

- Grade 4 neutropenia lasting > 7 days;
- ≥ grade 3 febrile neutropenia;
- Grade 4 thrombocytopenia;
- Grade 3 thrombocytopenia with bleeding;
- Grade 4 anemia.

(2) Non-hematologic toxicity:

- Class 4 non-hematologic toxicity;
- Grade 3 non-hematologic toxicity that dose recover to grade ≤2 within 3 days after treatment (except for simple laboratory abnormalities that are judged by the investigator to be asymptomatic and do not require intervention).

(3) Other toxic reactions that, in the judgement of the investigator, require permanent discontinuation of the study drug or result in the discontinuation of the first for more than 7 days.

The above AEs were graded according to the American Institute for Cancer Research's Routine Toxicity Criteria (NCI CTCAE) version 5.0.

DLT assesses cases in at least 75% of participants who have reached the planned dose administered.

## 4.4 Dose Adjustment and Treatment Delays

**4.4.1 Dose-escalation phase**

In the event of a non-DLT AE, medical management (including, but not limited to, dose interruption and symptomatic treatment) is in principle not indicated to observe the possible AEs of the investigational product and their severity and reversibility. However, in the event of a protocol-specified DLT toxicity, the drug must be immediately discontinued and actively managed, and the drug combination used must be documented in the original medical record. Subjects who experience a DLT and recover to ≤ grade 1 or baseline toxicity after treatment may be dosed at the original dose level (if the original dose is the lowest dose) or one dose level lower. Subjects were required to withdraw from the study if dosing is interrupted for more than 2 weeks.

After the determination of DLT and MTD, the management of adverse events in the first cycle of continuous medication refers to 4.4.2 *Continuous Drug Cycle 2 and Later, Dose-expansion Phase*.

**4.4.2** **Continuous Drug Cycle 2 and Later, Dose-expansion Phase**

After the occurrence of toxic reactions, the doctor can judge according to the situation and give corresponding treatment, the specific treatment principle can refer to the following table 4-6:

**Table 4-6 Guidelines for the adjustment of other toxic doses related to drugs**

| **Toxicity level** | **Doseage adjustment** |
| --- | --- |
| Grade 1 or 2 | Maintain dose |
| Grade 3, toxicity ≤ 28 days and recovery to grade ≤2/baseline | Suspeion, downward adjustment or permanently discontinue |
| Grade 3, toxicity lasting more than 28 days without recovery to grade ≤2/baseline | Permanent discontinuation |
| Grade 4 | Dose reduction or permanent discontinuation |

Principles of dose adjustment:

1) Controllable and reversible: grade 3 AE, first occurrence pause until recovery to ≤grade 2/baseline, continue dosing at original dose; second occurrence pause until recovery to ≤grade 2/baseline, adjust down 1 dose to the lowest dose, and permanent discontinuation if recurrent. grade 4 AE, first occurrence pause down 1 dose, and permanent discontinuation if recurrent.

1. Uncontrollable and irreversible: occurrence of grade 3-4 AEs lasting more than 28 days without recovery to ≤ grade 2/baseline, permanent discontinuation.

# 5. Research Follow-up Visit

The timing of each visit and the examinations to be performed in this study are detailed in the study flow charts 5-1 and 5-2, where 'X' indicates the examination or assessment to be performed. All information in the following tables should be supported by original documentation.

## 5.1 Research Follow-up visit Process

**Table 5-1 Dose-escalation phase study flow table - advanced solid tumors**

| **Research steps** | **Screening period^*^** | **Single-dose PK study period** | | | | | | | **Study treatment period 1** | | | | | | **End of treatment visits^20^** | **Safety follow-up^21^** |
| --- | --- | --- | --- | --- | --- | --- | --- | --- | --- | --- | --- | --- | --- | --- | --- | --- |
|  |  |  |  |  |  |  |  |  | **Cycle 1** | | | | | **Cycle 2~**  **(Follow-up every eight weeks)** |  |  |
| Day of the cycle | -28~-1 | 1 | 2 | 3 | 4 | 5 | 6 | 7 | 1^a^ | 8 ^b^ | 15 ^b^ | 22 ^b^ | 28 ^c^ | 28 ^c^ | 28c |  |
| Demographic information | X |  |  |  |  |  |  |  |  |  |  |  |  |  |  |  |
| History and treatment history ^2^ | X |  |  |  |  |  |  |  |  |  |  |  |  |  |  |  |
| RET genetic testing ^3^ | X |  |  |  |  |  |  |  |  |  |  |  |  |  |  |  |
| Physical examination ^4^ | X |  |  |  |  |  |  | X |  | X | X | X | X | X | X |  |
| ECOG score | X |  |  |  |  |  |  | X |  | X | X | X | X | X | X |  |
| Vital signs ^5^ | X | X | X | X | X | X | X | X | X | X | X | X | X | X | X |  |
| 12-lead ECG | X |  |  |  |  |  |  | X |  | X |  |  | X | X | X |  |
| Blood routine test ^6^ | X |  |  |  |  |  |  | X |  | X |  |  | X | X | Xd |  |
| Blood Biochemistry ^7^ | X |  |  |  |  |  |  | X |  | X |  |  | X | X | Xd |  |
| Pregnancy test ^8^ | X |  |  |  |  |  |  |  |  |  |  |  |  |  | Xd |  |
| Virological examination ^9^ | X |  |  |  |  |  |  |  |  |  |  |  |  |  | Xd |  |
| Thyroid function tests ^10^ | X |  |  |  |  |  |  | X |  |  |  |  | X | X | Xd |  |
| Coagulation four items11 | X |  |  |  |  |  |  | X |  |  |  |  | X | X | Xd |  |
| urinalysis ^12^ | X |  |  |  |  |  |  | X |  | X |  |  | X | X | Xd |  |
| Calcitonin and CEA (MTC only) ^13^ | X |  |  |  |  |  |  | X |  |  |  |  | X | X | Xd |  |
| Whole blood cfDNA analysis ^14^ | X |  |  |  |  |  |  |  |  |  |  |  | X (only for the first CR or PR). | | X |  |
| Whole blood DNA genome | X |  |  |  |  |  |  |  |  |  |  |  |  |  |  |  |
| Tumor evaluation ^15^ | X |  |  |  |  |  |  |  | X | | | | | | Xd |  |
| Tumor evaluation confirms ^16^ |  |  |  |  |  |  |  |  |  |  |  |  |  | At least 4 weeks after the initial CR/PR |  |  |
| Pharmacokinetic studies ^17^ |  | X | X | X | X | X | X | X | X |  |  |  | X |  |  |  |
| Take SY-5007 |  | X |  |  |  |  |  |  | X | X | X | X | X | X |  |  |
| Subject's medication diary |  | X |  |  |  |  |  |  | X | X | X | X | X | X |  |  |
| Combined medications ^18^ | X | X | X | X | X | X | X | X | X | X | X | X | X | X | X | X |
| Adver event ^19^ |  | X | X | X | X | X | X | X | X | X | X | X | X | X | X | X |

*: Routine tests during the screening period (blood routine, blood biochemistry, pregnancy test, virus serology, thyroid function test, coagulation function, urinalysis, calcitonin and CEA, 12-lead-ECG, etc.) need to be completed within 7 days before the first dose;

a: The window period is +1 day;

b: The window period is ±1 days;

c: The window period is ±3 days, and the imaging examination ± 7 days.

d: If blood routine, blood biochemistry, pregnancy test, viral serology, thyroid function test, coagulation function, urine routine, calcitonin and CEA, 12-lead-ECG within 7 days prior to this visit, and imaging evaluation is performed within 28 days prior to this visit, these tests do not need to be repeated for this visit.

| **Study Step Notes:** | |
| --- | --- |
| 1. Study and treatment period | Unless otherwise noted, *all tests and assessments need to be performed prior to taking the study drug*.  Please follow the notes for time windows. Subjects who do not return to the center for clinical visits should contact the investigator promptly for adverse events and co-medication. |
| 2. Medical history and treatment history | Medical and cancer history, surgical history, radiation therapy, and systemic anticancer therapy, including the names and dates of use of all VEGFR-targeted tyrosine kinase inhibitors. |
| 3. RET genetic testing | Subjects provide blood samples, available tissue or biopsy tissue for RET gene testing whenever possible, even if a positive RET test report can be provided. The methods used to detect the RET gene are RT-PCR, IHC, NGS and FISH. Specific requirements for the testing of samples can be found in the Central Laboratory Manual. |
| 4. Physical examination | Physical examination of relevant systems and review of results, measurement of weight and height are performed during the screening period. The physical examination includes general condition, skin and mucous membranes, superficial lymph nodes, head and its organs, neck, chest, abdomen, spine and extremities, nervous system, and others. A symptom-specific physical examination, including weight measurement, may be performed at other times. |
| 5. Vital signs | Systolic and diastolic blood pressure, pulse, respiratory rate and body temperature. |
| 6. Blood routine | White blood cell count (WBC), neutrophil count (ANC), eosinophil count (EO), basophil count (BASO), lymphocytes count (LYMPH), red blood cell count (RBC), hemoglobin (HB), platelet count (PLT), hematocrit (HCT), monocytes count (MONO); |
| 7. Blood biochemistry | Total bilirubin (TBIL), total protein (TP), albumin (ALB), alanine aminotransferase (ALT), glutamyl aminotransferase (AST), glutamyl transferase (γ-GT), creatinine (Cr), total cholesterol (CHOL), triglycerides (TG), blood urea (Urea), alkaline phosphatase (ALP), blood glucose (GLU), serum sodium (Na+), serum potassium (K+), serum calcium (Ca2+), serum magnesium (Mg2+), serum chlorine (CL-) , serum creatine kinase (CK), serum creatine kinase isoenzyme (CK-MB), serum lactate dehydrogenase (LDH); |
| 8. Pregnancy test | Blood pregnancy tests were performed at baseline (-7 to -1 day) and 28 days after the last dose and were performed only in non-menopausal women of childbearing potential, during the study period as needed at the discretion of the investigator, and in women of childbearing potential. Surgically sterilized women and women who have been menopausal for at least 2 years are not required to be tested. |
| 9.Virological examination | Hepatitis B surface antigen, hepatitis B surface antibody, hepatitis B e antigen, hepatitis B e antibody, hepatitis B core antibody (the investigator selected hepatitis B virus DNA if necessary based on the five results of hepatitis B and related medical history, and allowed to send hepatitis B virus DNA for simultaneous testing), hepatitis C virus antibody, HIV antibody and treponemal antibody; |
| 10. Thyroid function | Thyroid-stimulating hormone (TSH), free triiodothyronine (T3), free thyroxine (T4). |
| 11. Four items of blood coagulation | International normalized ratio (INR), activated partial thromboplastin time (APTT), prothrombin time (PT), fibrinogen (FIB); |
| 12. Urinalysis | pH, urine protein, ketone body, urine specific gravity, urine occult blood, urine leukocytes, urine red blood cells, urine nitrite, urine bilirubin; |
| 13. Calcitonin and CEA | Calcitonin and CEA are only available to participants diagnosed with MTC. These operations should be performed in the same laboratory to minimize differences between the subjects. |
| 14. Whole blood cfDNA analysis | Whole blood should be collected for cfDNA analysis at screening, at the first efficacy assessment of CR or PR, and at disease progression. Whole blood should be collected for cfDNA analysis at the EOT visit even if disease imaging has not been performed. SY-5007 will be discontinued if disease progression due to oligometastasis is not considered to be of benefit to the subject in the opinion of the investigator, otherwise the drug may be continued and the subject's blood may be collected for cfDNA analysis. The volume of blood collected for each cfDNA analysis is 10 mL. |
| 15. Tumor assessment | Baseline imaging assessment of the neck, chest, abdomen (total), pelvis, or any other suspected lesion area using CT or MRI during the baseline period (within 28 days prior to the first dose). Bone scans are required at baseline. Subsequent tumour assessment for brain and/or bone metastases is required if the subject has brain and/or bone metastases at baseline (MRI is preferred for brain metastases and enhanced CT is acceptable if there is a contraindication to MRI). Imaging studies will be accepted with results obtained up to 28 days prior to the first dose of medication. Bone scans are accepted up to 3 months prior to the first dose.  Tumour imaging assessments, including neck (not required for follow-up if no involvement at baseline), chest, abdomen, and pelvis, should be performed every 8 weeks (±7 days) from C1D1, using the same modality as the baseline imaging assessment, until disease progression, withdrawal of consent, or initiation of new anticancer therapy. The investigator may increase the number of examinations based on the subject's actual condition. In addition, any site with a positive result at the time of the baseline imaging assessment should be repeated at all post-baseline assessments. Additional investigations may be performed based on clinical presentation.  If a subject discontinues study treatment for reasons other than disease progression (e.g., AEs, poor compliance), arrangements should be made for the subject to complete follow-up imaging assessments whenever possible (but not necessary for practical reasons and to minimise inconvenience to the subject) until the subject's disease has progressed, informed consent has been withdrawn, or new antineoplastic therapy has been initiated. |
| 16. Tumor assessment and confirmation | According to the RECIST 1.1 criteria, researchers confirmed tumor evaluation after at least 4 weeks (28 days) in subjects who received CR or PR efficacy in initial tumor evaluation at C1D28 (± 7 days). |
| 17. Pharmacokinetic studies | **Dose-escalation phase - QD administration**: Subjects will first undergo a single-dose pharmacokinetic (PK) study, i.e., 1 dose followed by 7 days of observation, with blood collection points including before the first dose (within 0.5 h), 0.5, 1, 2, 4, 6, 8, 12, 24 (D2), 48 (D3), 72 (D4), 96 (D5), 120 (D6), 144 (D7) and 168 h (C1D1 before dosing). A PK study is then performed with once daily continuous dosing for 28 days per cycle. Blood collection points including pre-dose (within 0.5 hours), and 0.5, 1, 2, 4, 6, 8, 12, and 24 h post-dose on day 28 of cycle 1 (window period ±3).  **Dose-escalation phase - BID administration**: Subjects will first undergo a single-dose pharmacokinetic (PK) study, i.e., 1 dose followed by 7 days of observation, with blood collection points including before the first dose (within 0.5 h), 0.5, 1, 2, 4, 6, 8, 12, 24 (D2), 48 (D3), 72 (D4), 96 (D5), 120 (D6), 144 (D7) and 168 h (C1D1 before dosing). A PK study is then performed with two daily continuous dosing for 28 days per cycle. Blood collection points including pre-dose (within 0.5 hours), and 0.5, 1, 2, 4, 6, 8, and12 h post-dose on day 28 of cycle 1 (window period ±3).  The PK blood collection point can be adjusted according to the PK results.  PK parameters, including but not limited to C_max_, C_trough_, AUC_0~24_, AUC_0~∞_, T_max,_ t_1/2_, bioavailability-corrected clearance CL/F, bioavailability-corrected apparent volume of distribution Vd/F and accumulation index Rac. |
| 18. Combined medication | Records of concomitant medications should be recorded from 28 days prior to study treatment to 28 days after the last treatment (window period of ±3 days). |
| 19. Adverse events | Adverse events were recorded from the time of the first dose until 28 days after the last dose of study drug (a window of ±3 days) or until complete remission of all serious or drug-related toxicities or confirmation of conversion to chronic or stable disease, whichever is later.  Serious adverse events should be monitored and reported from subjects after taking the drug in accordance with the protocol. In this protocol, the CTCAE version 5.0 standard is used for adverse event classification. |
| 20. End-of-treatment visit | Refers to the 28 days after the last dose of the test drug, during which all participants are required to complete end-of-treatment follow-up, even if the participant ends treatment early. |
| 21. Safety follow-up | If the subject is permanently off medication because of an adverse event or abnormal laboratory test, he or she should be followed weekly for the first 4 weeks of follow-up, and may be followed every 4 weeks thereafter until resolution or stabilization of the adverse event. If the subject has to delay taking the medication for more than 2 weeks because of a trial drug-related adverse event, the subject should be withdrawn from the trial, but follow-up of the resolution of the adverse event must continue until resolution, stabilization, or the subject is lost to follow-up. |

**Table 5-2 Dose-expansion Phase Study Flow Chart - Advanced Solid Tumors with RET Gene Variants**

| **Research steps** | **Screening period^*^** | **Study treatment period 1** | | | | | | **End of treatment visits^20^** | **Safety follow-up^21^** |
| --- | --- | --- | --- | --- | --- | --- | --- | --- | --- |
|  |  | **Cycle 1** | | | | | **Cycle 2 ~ (follow-up every eight weeks)** |  |  |
| Day of the cycle | -28~-1 | 1 | 8 ^a^ | 15 ^a^ | 22 ^a^ | 28 ^b^ | 28 ^b^ | 28b |  |
| Demographic information | X |  |  |  |  |  |  |  |  |
| History and treatment history ^2^ | X |  |  |  |  |  |  |  |  |
| RET genetic testing ^3^ | X | X | X | X | X | X | X | X |  |
| Physical examination ^4^ | X | X | X | X | X | X | X | X |  |
| ECOG score | X | X | X | X | X | X | X | X |  |
| Vital signs ^5^ | X | X | X |  |  | X | X | X |  |
| 12-lead ECG | X |  | X |  |  | X | X | Xc |  |
| Blood routine test ^6^ | X |  | X |  |  | X | X | Xc |  |
| Blood Biochemistry ^7^ | X |  |  |  |  |  |  | Xc |  |
| Pregnancy test ^8^ | X |  |  |  |  |  |  | Xc |  |
| Virological examination ^9^ | X |  |  |  |  | X | X | Xc |  |
| Thyroid function tests ^10^ | X |  |  |  |  | X | X | Xc |  |
| Coagulation four items11 | X |  | X |  |  | X | X | Xc |  |
| urinalysis ^12^ | X |  |  |  |  | X | X | Xc |  |
| Calcitonin and CEA (MTC only) ^13^ | X |  |  |  |  | X (only for the first CR or PR). | | X |  |
| Whole blood cfDNA analysis ^14^ | X |  |  |  |  |  |  |  |  |
| Whole blood DNA genome | X | X | | | | | | Xc |  |
| Tumor evaluation ^15^ |  |  |  |  |  |  | At least 4 weeks after the first CR/PR |  |  |
| Tumor evaluation confirms ^16^ |  | X |  |  |  | X |  |  |  |
| Pharmacokinetic studies ^17^ |  | X | X | X | X | X | X |  |  |
| Take SY-5007 |  | X | X | X | X | X | X |  |  |
| Subject's medication diary | X | X | X | X | X | X | X | X | X |
| Combined medications ^18^ |  | X | X | X | X | X | X | X | X |
| Adver event ^19^ |  |  |  |  |  |  |  |  |  |

*: Routine tests during the screening period (blood routine, blood biochemistry, pregnancy test, virus serology, thyroid function test, coagulation function, urinalysis, calcitonin and CEA, 12-lead-ECG, etc.) need to be completed within 7 days before the first dose;

a: The window period is +1 day;

b: The window period is ±1 days;

c: The window period is ±3 days, and the imaging examination ± 7 days.

d: If blood routine, blood biochemistry, pregnancy test, viral serology, thyroid function test, coagulation function, urine routine, calcitonin and CEA, 12-lead-ECG within 7 days prior to this visit, and imaging evaluation is performed within 28 days prior to this visit, these tests do not need to be repeated for this visit.

| **Study Step Notes:** | |
| --- | --- |
| 1. Study treatment period | Unless otherwise noted, *all tests and assessments need to be performed prior to taking the study drug*.  Please follow the notes for time windows. Subjects who do not return to the center for clinical visits should contact the investigator promptly for adverse events and co-medication. |
| 2. Medical history and treatment history | Medical and cancer history, surgical history, radiation therapy, and systemic anticancer therapy, including the names and dates of use of all VEGFR-targeted tyrosine kinase inhibitors. |
| 3. RET genetic testing | Subjects provide blood samples, available tissue or biopsy tissue for RET gene testing whenever possible, even if a positive RET test report can be provided. The methods used to detect the RET gene are RT-PCR, IHC, NGS and FISH. Specific requirements for the testing of samples can be found in the Central Laboratory Manual. |
| 4. Physical examination | Physical examination of relevant systems and review of results, measurement of weight and height are performed during the screening period. The physical examination includes general condition, skin and mucous membranes, superficial lymph nodes, head and its organs, neck, chest, abdomen, spine and extremities, nervous system, and others. A symptom-specific physical examination, including weight measurement, may be performed at other times. |
| 5. Vital signs | Systolic and diastolic blood pressure, pulse, respiratory rate and body temperature. |
| 6. Blood routine | White blood cell count (WBC), neutrophil count (ANC), eosinophil count (EO), basophil count (BASO), lymphocytes count (LYMPH), red blood cell count (RBC), hemoglobin (HB), platelet count (PLT), hematocrit (HCT), monocytes count (MONO); |
| 7. Blood biochemistry | Total bilirubin (TBIL), total protein (TP), albumin (ALB), alanine aminotransferase (ALT), glutamyl aminotransferase (AST), glutamyl transferase (γ-GT), creatinine (Cr), total cholesterol (CHOL), triglycerides (TG), blood urea (Urea), alkaline phosphatase (ALP), blood glucose (GLU), serum sodium (Na+), serum potassium (K+), serum calcium (Ca2+), serum magnesium (Mg2+), serum chlorine (CL-) , serum creatine kinase (CK), serum creatine kinase isoenzyme (CK-MB), serum lactate dehydrogenase (LDH); |
| 8. Pregnancy test | Blood pregnancy tests were performed at baseline (-7 to -1 day) and 28 days after the last dose and were performed only in non-menopausal women of childbearing potential, during the study period as needed at the discretion of the investigator, and in women of childbearing potential. Surgically sterilized women and women who have been menopausal for at least 2 years are not required to be tested. |
| 9.Virological examination | Hepatitis B surface antigen, hepatitis B surface antibody, hepatitis B e antigen, hepatitis B e antibody, hepatitis B core antibody (the investigator selected hepatitis B virus DNA if necessary based on the five results of hepatitis B and related medical history, and allowed to send hepatitis B virus DNA for simultaneous testing), hepatitis C virus antibody, HIV antibody and treponemal antibody; |
| 10. Thyroid function | Thyroid-stimulating hormone (TSH), free triiodothyronine (T3), free thyroxine (T4). |
| 11. Four items of blood coagulation | International normalized ratio (INR), activated partial thromboplastin time (APTT), prothrombin time (PT), fibrinogen (FIB); |
| 12. Urinalysis | pH, urine protein, ketone body, urine specific gravity, urine occult blood, urine leukocytes, urine red blood cells, urine nitrite, urine bilirubin; |
| 13. Calcitonin and CEA | Calcitonin and CEA are only available to participants diagnosed with MTC. These operations should be performed in the same laboratory to minimize differences between the subjects. |
| 14. Whole blood cfDNA analysis | Whole blood should be collected for cfDNA analysis at screening, at the first efficacy assessment of CR or PR, and at disease progression. Whole blood should be collected for cfDNA analysis at the EOT visit even if disease imaging has not been performed. SY-5007 will be discontinued if disease progression due to oligometastasis is not considered to be of benefit to the subject in the opinion of the investigator, otherwise the drug may be continued and the subject's blood may be collected for cfDNA analysis. The volume of blood collected for each cfDNA analysis is 10 mL. |
| 15. Tumor assessment | Baseline imaging assessment of the neck, chest, abdomen (total), pelvis, or any other suspected lesion area using CT or MRI during the baseline period (within 28 days prior to the first dose). Bone scans are required at baseline. Subsequent tumour assessment for brain and/or bone metastases is required if the subject has brain and/or bone metastases at baseline (MRI is preferred for brain metastases and enhanced CT is acceptable if there is a contraindication to MRI). Imaging studies will be accepted with results obtained up to 28 days prior to the first dose of medication. Bone scans are accepted up to 3 months prior to the first dose.  Tumour imaging assessments, including neck (not required for follow-up if no involvement at baseline), chest, abdomen, and pelvis, should be performed every 8 weeks (±7 days) from C1D1, using the same modality as the baseline imaging assessment, until disease progression, withdrawal of consent, or initiation of new anticancer therapy. The investigator may increase the number of examinations based on the subject's actual condition. In addition, any site with a positive result at the time of the baseline imaging assessment should be repeated at all post-baseline assessments. Additional investigations may be performed based on clinical presentation.  If a subject discontinues study treatment for reasons other than disease progression (e.g., AEs, poor compliance), arrangements should be made for the subject to complete follow-up imaging assessments whenever possible (but not necessary for practical reasons and to minimise inconvenience to the subject) until the subject's disease has progressed, informed consent has been withdrawn, or new antineoplastic therapy has been initiated. |
| 16. Tumor assessment and confirmation | According to the RECIST 1.1 criteria, researchers confirmed tumor evaluation after at least 4 weeks (28 days) in subjects who received CR or PR efficacy in initial tumor evaluation at C1D28 (± 7 days). |
| 17. Pharmacokinetic studies | **Dose-escalation phase - QD administration**: Subjects will first undergo a single-dose pharmacokinetic (PK) study, i.e., 1 dose followed by 7 days of observation, with blood collection points including before the first dose (within 0.5 h), 0.5, 1, 2, 4, 6, 8, 12, 24 (D2), 48 (D3), 72 (D4), 96 (D5), 120 (D6), 144 (D7) and 168 h (C1D1 before dosing). A PK study is then performed with once daily continuous dosing for 28 days per cycle. Blood collection points including pre-dose (within 0.5 hours), and 0.5, 1, 2, 4, 6, 8, 12, and 24 h post-dose on day 28 of cycle 1 (window period ±3).  **Dose-escalation phase - BID administration**: Subjects will first undergo a single-dose pharmacokinetic (PK) study, i.e., 1 dose followed by 7 days of observation, with blood collection points including before the first dose (within 0.5 h), 0.5, 1, 2, 4, 6, 8, 12, 24 (D2), 48 (D3), 72 (D4), 96 (D5), 120 (D6), 144 (D7) and 168 h (C1D1 before dosing). A PK study is then performed with two daily continuous dosing for 28 days per cycle. Blood collection points including pre-dose (within 0.5 hours), and 0.5, 1, 2, 4, 6, 8, and12 h post-dose on day 28 of cycle 1 (window period ±3).  The PK blood collection point can be adjusted according to the PK results.  PK parameters, including but not limited to C_max_, C_trough_, AUC_0~24_, AUC_0~∞_, T_max,_ t_1/2_, bioavailability-corrected clearance CL/F, bioavailability-corrected apparent volume of distribution Vd/F and accumulation index Rac. |
| 18. Combined medication | Records of concomitant medications should be recorded from 28 days prior to study treatment to 28 days after the last treatment (window period of ±3 days). |
| 19. Adverse events | Adverse events were recorded from the time of the first dose until 28 days after the last dose of study drug (a window of ±3 days) or until complete remission of all serious or drug-related toxicities or confirmation of conversion to chronic or stable disease, whichever was later.  Serious adverse events should be monitored and reported from subjects after taking the drug in accordance with the protocol. In this protocol, the CTCAE version 5.0 standard is used for adverse event classification. |
| 20. End-of-treatment visit | Refers to the 28 days after the last dose of the test drug, during which all participants are required to complete end-of-treatment follow-up, even if the participant ends treatment early. |
| 21. Safety follow-up | If the subject is permanently off medication because of an adverse event or abnormal laboratory test, he or she should be followed weekly for the first 4 weeks of follow-up, and may be followed every 4 weeks thereafter until resolution or stabilization of the adverse event. If the subject has to delay taking the medication for more than 2 weeks because of a trial drug-related adverse event, the subject should be withdrawn from the trial, but follow-up of the resolution of the adverse event must continue until resolution, stabilization, or the subject is lost to follow-up. |

### 5.1.1 Screening Visits

Screening assessment must be performed within 28 days prior to the first dose of medication; routine tests (routine blood count, blood biochemistry, pregnancy test, viral serology, thyroid function tests, coagulation, routine urinalysis, calcitonin and CEA, 12-lead ECG) must be completed within 7 days prior to the first dose of medication; imaging tests are accepted within 28 days prior to the first dose of medication. Bone scans were accepted up to 3 months prior to dosing.

### 5.1.2 PK Research

**Dose-escalation Phase - QD administration**: Subjects will first undergo a single-dose pharmacokinetic (PK) study, i.e., 1 dose followed by 7 days of observation, with blood collection points including before the first dose (within 0.5 h), 0.5, 1, 2, 4, 6, 8, 12, 24 (D2), 48 (D3), 72 (D4), 96 (D5), 120 (D6), 144 (D7) and 168 h (C1D1 before dosing). A PK study is then performed with once daily continuous dosing for 28 days per cycle. Blood collection points including pre-dose (within 0.5 hours), and 0.5, 1, 2, 4, 6, 8, 12, and 24 h post-dose on day 28 of cycle 1 (window period ±3).

**Dose-escalation Phase - BID administration**: Subjects will first undergo a single-dose pharmacokinetic (PK) study, i.e., 1 dose followed by 7 days of observation, with blood collection points including before the first dose (within 0.5 h), 0.5, 1, 2, 4, 6, 8, 12, 24 (D2), 48 (D3), 72 (D4), 96 (D5), 120 (D6), 144 (D7) and 168 h (C1D1 before dosing). A PK study is then performed with two daily continuous dosing for 28 days per cycle. Blood collection points including pre-dose (within 0.5 hours), and 0.5, 1, 2, 4, 6, 8, and12 h post-dose on day 28 of cycle 1 (window period ±3).

The PK blood collection point can be adjusted according to according to the PK results of previous subjects.

**Dose expansion phase**: Subjects will be directly enrolled in the PK study with twice-daily continuous dosing for 28 days per cycle. Blood collection points include days 1 and 28 of cycle 1 and 28 (window period is ±3 days), before (within 0.5 h), and 0.5, 1, 2, 4, 6, 8, and 12 hours after the first dose.

The PK blood collection point can be adjusted according to according to the PK results of previous subjects.

3 mL of whole blood is collected at each of the above blood collection sites and processed, preserved and tested by qualified personnel according to the SOPs. Specific procedures for collection, preservation and processing of blood samples are described in the Centre's Laboratory Manual.

**Table 5-4 Pharmacokinetic parameters**

| C_max_ | Maximum plasma concentration |
| --- | --- |
| C_max,ss_ | Steady-state maximum plasma concentration |
| C_trough_ | Steady state minimum plasma concentration |
| T_max_ | Peak time |
| t_1/2_ | Eliminates half-life |
| AUC _0-t_ | Area under the curve within t hours post-dose |
| AUC _0-∞_ | Area under the 0-infinite curve post-dose |
| AUC_ss_ | The area under the curve at steady state post-dose |
| CL/F | Bioavailability-corrected clearance |
| CEO/F | Bioavailability-corrected apparent volume of distribution |
| Rac | Accumulation index |

**Table 5-5 Sample Collection Schedule (Dose-escalation-QD Administration)**

| **Sample number** | **Study cycle** | **Day** | **Time** | **Medication** |
| --- | --- | --- | --- | --- |
| 01 | PK study period | 1 | 0 h | Before the first dose |
| 02 | PK study period | 1 | 0.5 h（±5 minutes） | After a single dose |
| 03 | PK study period | 1 | 1 h（±10 minutes） | After a single dose |
| 04 | PK study period | 1 | 2 h（±10 minutes） | After a single dose |
| 05 | PK study period | 1 | 4 h（±10 minutes） | After a single dose |
| 06 | PK study period | 1 | 6 h（±10 minutes） | After a single dose |
| 07 | PK study period | 1 | 8 h（±10 minutes） | After a single dose |
| 08 | PK study period | 1 | 12 h（±10 minutes） | After a single dose |
| 09 | PK study period | 2 | 24 h（±60 minutes） | After a single dose |
| 10 | PK study period | 3 | 48 h（±60 minutes） | After a single dose |
| 11 | PK study period | 4 | 72 h（±60 minutes） | After a single dose |
| 12 | PK study period | 5 | 96 h（±60 minutes） | After a single dose |
| 13 | PK study period | 6 | 120 h（±60 minutes） | After a single dose |
| 14 | PK study period | 7 | 144h（±60 minutes） | After a single dose |
| 15 | PK study period | 8 | 168 h（±60 minutes） | After a single dose |
| 16 | C1 | 28 | 0 h | Day 28 of continuous dosing-prior dose |
| 17 | C1 | 28 | 0.5 h（±5 minutes） | Day 28 of continuous dosing-post dose |
| 18 | C1 | 28 | 1 h（±10 minutes） | Day 28 of continuous dosing-post dose |
| 19 | C1 | 28 | 2 h（±10 minutes） | Day 28 of continuous dosing-post dose |
| 20 | C1 | 28 | 4h（±10 minutes） | Day 28 of continuous dosing-post dose |
| 21 | C1 | 28 | 6 h（±10 minutes） | Day 28 of continuous dosing-post dose |
| 22 | C1 | 28 | 8 h（±10 minutes） | Day 28 of continuous dosing-post dose |
| 23 | C1 | 28 | 12 h（±10 minutes） | Day 28 of continuous dosing-post dose |
| 24 | C1 | 28 | 24 h（±60 minutes） | Day 28 of continuous dosing-post dose, C2D1 |

**Table 5-6 Sample Collection Schedule (Dose-sscalation-BID Administration)**

| **Sample number** | **Study cycle** | **Day** | **Time** | **Medication** |
| --- | --- | --- | --- | --- |
| 01 | PK study period | 1 | 0 h | Before the first dose |
| 02 | PK study period | 1 | 0.5 h（±5 minutes） | After a single dose |
| 03 | PK study period | 1 | 1 h（±10 minutes） | After a single dose |
| 04 | PK study period | 1 | 2 h（±10 minutes） | After a single dose |
| 05 | PK study period | 1 | 4 h（±10 minutes） | After a single dose |
| 06 | PK study period | 1 | 6 h（±10 minutes） | After a single dose |
| 07 | PK study period | 1 | 8 h（±10 minutes） | After a single dose |
| 08 | PK study period | 1 | 12 h（±10 minutes） | After a single dose |
| 09 | PK study period | 2 | 24 h（±60 minutes） | After a single dose |
| 10 | PK study period | 3 | 48 h（±60 minutes） | After a single dose |
| 11 | PK study period | 4 | 72 h（±60 minutes） | After a single dose |
| 12 | PK study period | 5 | 96 h（±60 minutes） | After a single dose |
| 13 | PK study period | 6 | 120 h（±60 minutes） | After a single dose |
| 14 | PK study period | 7 | 144h（±60 minutes） | After a single dose |
| 15 | PK study period | 8 | 168 h（±60 minutes） | After a single dose |
| 16 | C1 | 28 | 0 h | Day 28 of continuous dosing-prior dose |
| 17 | C1 | 28 | 0.5 h（±5 minutes） | Day 28 of continuous dosing-post dose |
| 18 | C1 | 28 | 1 h（±10 minutes） | Day 28 of continuous dosing-post dose |
| 19 | C1 | 28 | 2 h（±10 minutes） | Day 28 of continuous dosing-post dose |
| 20 | C1 | 28 | 4h（±10 minutes） | Day 28 of continuous dosing-post dose |
| 21 | C1 | 28 | 6 h（±10 minutes） | Day 28 of continuous dosing-post dose |
| 22 | C1 | 28 | 8 h（±10 minutes） | Day 28 of continuous dosing-post dose |
| 23 | C1 | 28 | 12 h（±10 minutes） | Day 28 of continuous dosing-post dose |

**Table 5-7 Sample Collection Schedule (Dose-expansion)**

| **Sample number** | **Study cycle** | **Day** | **Time** | **Medication** |
| --- | --- | --- | --- | --- |
| 01 | C1 | 1 | 0 h | Before the first dose |
| 02 | C1 | 1 | 0.5 h（±5 minutes） | After a single dose |
| 03 | C1 | 1 | 1 h（±10 minutes） | After a single dose |
| 04 | C1 | 1 | 2 h（±10 minutes） | After a single dose |
| 05 | C1 | 1 | 4h（±10 minutes） | After a single dose |
| 06 | C1 | 1 | 6 h（±10 minutes） | After a single dose |
| 07 | C1 | 1 | 8 h（±10 minutes） | After a single dose |
| 08 | C1 | 1 | 12 h（±60 minutes） | After a single dose |
| 09 | C1 | 28 | 0 h | Day 28 of continuous dosing-prior dose |
| 10 | C1 | 28 | 0.5 h（±5 minutes） | Day 28 of continuous dosing-post dose |
| 11 | C1 | 28 | 1 h（±10 minutes） | Day 28 of continuous dosing-post dose |
| 12 | C1 | 28 | 2 h（±10 minutes） | Day 28 of continuous dosing-post dose |
| 13 | C1 | 28 | 4h（±10 minutes） | Day 28 of continuous dosing-post dose |
| 14 | C1 | 28 | 6 h（±10 minutes） | Day 28 of continuous dosing-post dose |
| 15 | C1 | 28 | 8 h（±10 minutes） | Day 28 of continuous dosing-post dose |
| 16 | C1 | 28 | 12 h（±10 minutes） | Day 28 of continuous dosing-post dose |

### 5.1.3 Study Treatment Period

The treatment period is defined as the period from the first day of continuous dosing to the last dose of the drug.

Subjects entering the treatment period will take the drug continuously on a daily basis until a progressive or intolerable toxic reaction occurs, the investigator or the subject decides to discontinue treatment, start a new anti-tumor therapy, or die.

### 5.1.4 End-of-treatment Visit

Within 28 days of the last dose of study drug, all subjects will be required to attend an end-of-treatment visit during this period, which will include the completion of examinations and assessments such as:

- Physical examination
- ECOG score
- Vital signs
- 12-lead ECG
- Blood routine
- Blood biochemistry
- Pregnancy test
- Virological examination
- Thyroid function tests
- Coagulation function
- Urinalysis
- Calcitonin and CEA (MTC only)
- Whole blood cfDNA analysis
- Tumor evaluation
- Adverse events and concomitant drugs were assessed.

If blood routine, blood biochemistry, pregnancy test, viral serology, thyroid function test, coagulation function, urinalysis, calcitonin and CEA, 12-lead-ECG were received within 7 days prior to the visit, and imaging evaluation is performed within 28 days prior to the visit, these tests do not need to be repeated for this visit.

All subjects who withdraw from the study should be contacted, if possible, and the subject should be asked to complete a safety visit and be informed of other antineoplastic treatments that have been given within 28 days of withdrawal from the study and recorded in the CRF. If the subject is temporarily or permanently off medication because of an adverse event or abnormal laboratory test, he/she should be followed up weekly for the first 4 weeks of follow-up, and may be followed up every 4 weeks thereafter until the AE resolves, stabilises, or disappears. If a subject needs to delay taking medication for more than 2 weeks because of a trial drug-related AE, the subject should be withdrawn from the trial, but follow-up for resolution of the AE should continue until recovery, stabilisation, or the subject is lost to follow-up. If the subject is lost to follow-up, this should be recorded on the CRF.

### 5.1.5 Unscheduled Visits

Any study-related visits that occur between scheduled visits should be documented in the eCRF. The following process should be performed at all study-related unscheduled visits: evaluation of observed or reported adverse events, including those related to co-administration. Other assessments will be performed at the investigator's discretion.

## 5.2 Efficacy Evaluation

Tumor assessment in this trial is performed according to RECIST 1.1 criteria (see *Appendix 15.2* for details). Imaging should be performed according to a strict schedule regardless of treatment delay. Subjects with a first remission assessment (CR/PR) require confirmation of efficacy after 4 weeks.

## 5.3 Safety Evaluation

To assess the safety of subjects from the time of signing the ICF until 28 days after the last dose according to the CTCAE 5.0 criteria. The following parameters must be documented to assess drug safet:

- AE、ADR、SAE、SUSAR,etc.
- Laboratory tests
- Physical examination
- Vital signs
- 12-lead ECG
- ECOG score, etc
- Delay and/or interruption of treatment due to AEs.

### 5.3.1 Vital Signs

Vital signs include temperature, pulse, blood pressure and respiration. Subjects are required to rest for at least 5 minutes prior to vital sign assessment, and blood pressure is measured in the same arm each time. During the course of the study, the investigator may add other vital signs as needed (e.g., for safety reasons).

### 5.3.2 Physical Examination (including weight)

A complete physical examination is required from the baseline visit to the end-of-study treatment visit. The complete physical examination includes general condition, skin and mucous membranes, superficial lymph nodes, head and its organs, neck, chest, abdomen, spine, extremities, nervous system, and others. The physical examination requires weight measurement (kg), during which the subjects must remove their shoes and wear indoor clothing. The investigator may add additional physical examinations as appropriate, as described in the study flowcharts 5-1 and 5-2. Significant clinical findings from the physical examination should be documented in the original medical record.

### 5.3.3 Laboratory Tests

Laboratory tests will be performed as specified in study flowcharts 5-1 and 5-2, and additional tests may be performed as clinically indicated. All tests must be recorded in the eCRF, especially clinically significant abnormal laboratory values (e.g. requiring dose adjustment, treatment interruption, treatment delay, causing clinical signs and symptoms, requiring clinical intervention, etc.). If an abnormal test result is an adverse event, it must be recorded on the adverse event page.

### 5.3.4 ECG Examination

The 12-lead ECG should be performed with the subject in the supine position after adequate rest and one ECG at each time point, recording heart rate, PR, QRS, uncorrected QT and Fridericia corrected QTc (QTcF). Depending on the study centre, QTcF may be calculated using Fridericia's formula or obtained directly from the ECG. If the QTcF interval is prolonged (>500 msec), it should be rechecked.

Additional ECGs may be performed if clinically indicated or if clinically difficult to determine. Only clinically significant abnormalities need to be recorded on the AE form. Clinically significant baseline/screening ECG abnormalities must be recorded on the appropriate eCRF page.

# 6. Experimental Drug

## 6.1 Experimental Drug

SY-5007: 20 mg, 80 mg, the dosage and mode of administration are as prescribed by follow the physician. It is recommended that the drug should be taken at the same time every day, and the actual time of taking the drug should be based on the original clinical records. In order to prevent food from affecting drug absorption, the drug should be taken on an empty stomach, that is, 1 hour before or 2 hours after meals. If the dose is missed, it can be taken within 4 hours of the expected dose time. If the drug has not been taken for more than 4 hours, no refills will be made and the drug will be taken at the expected time. If participants vomit after taking the drug, no refills will be given.

Treatment will continue until the participant develops disease progression, intolerable toxicity, a decision by the investigator or subject to discontinue treatment, initiation of a new antineoplastic therapy, or death.

## 6.2 Supply, Storage and Distribution of Test Drugs

The test drugs should be signed for, counted and stored securely by the designated research center personnel. Store sealed at room temperature (10~30°C) and protected from light.

At the start of the study, the appropriate dose and size of test drug will be dispensed according to the dose level at which the subject is placed.

The test drug SY-5007 will be provided to the subject at each return visit to the hospital and the test drug will be dispensed on a daily basis according to the period of the follow-up visit and the corresponding window period drug.

## 6.3 Assessment of Treatment Adherence

The investigator should assess the number of drugs taken by the subject and related information at each follow-up visit and document this in the chart notes for each follow-up visit. The investigator and appropriate personnel should ensure that the transfer and dispensing of drugs are accurately recorded. Subjects should return unused drugs and used drug packages as specified at the visit.

At the end of the trial or at other possible times, Shouyao Holdings is required to destroy any used or unused medication and packaging, and record this in the investigator's file.

## 6.4 Combination of Drugs

### 6.4.1 Combination of Drugs Allowed in This Study

The investigator may initiate appropriate supportive treatment after assessing the relationship of the AE to the study drug. The initiation and duration of supportive treatments should be recorded. These treatments include anti-emetic, anti-diarrheal, antipyretic, anti-allergic, treatment of rash and hand-foot syndrome, use of antihypertensive medication, use of analgesics, use of antibiotics, and others such as blood products;

- Non-hematologic toxicity may be treated with appropriate drugs at grade 2;
- Hematological toxicity can be treated with appropriate drugs at grade 3;
- Symptomatic treatment for common AEs is allowed during the trial.

Local treatment with radiotherapy and surgery is allowed if the subject, although considered to have progressive disease, is still considered by the investigator to have benefited from treatment with the study drug. Theoretical caution should be exercised as the effect of SY-5007 on radiotherapy and surgery is not clear and no studies are available. Treatment with SY-5007 should be interrupted during local treatment and should be stopped for at least 2 days prior to the start of local treatment and resumed with SY-5007 2 days after the end of local treatment.

Subjects should inform the investigator immediately if they are using any of the above treatments during the study period, and all information regarding co-administration of drugs (generic name of the medication, purpose of the medication, dosage of the medication, duration of medication, etc.) should be recorded in detail in the CRF.

### 6.4.2 Combination of Drugs Prohibited in This Study

**Prohibited drugs**

- The use of other anti-tumor therapies other than the test drug is prohibited during the study, including chemotherapy, targeted therapy, radiotherapy, etc. (except as permitted by 6.4.1);
- Strong inducers of CYP3A were not permitted during the study, including but not limited to: carbamazepine, phenobarbital, phenytoin, rifabutin, rifampicin, rifapentine, tipranavir, ritonavir, and St. John's wort;
- Strong inhibitors of CYP3A, including but not limited to: atazanavir, clarithromycin, indinavir, itraconazole, ketoconazole, nefazodone, nelfinavir, saquinavir, telithromycin, vinpocetine, voriconazole;
- Drugs that prolong the QTc interval of the heart, including but not limited to the following classes of drugs:
  - 1. Antiarrhythmics (quinidine, sotalol, amiodarone, disopyramide, procainamide);
    2. Antipsychotics (risperidone, fluphenazine, haloperidol, thioridazine, pimozide, olanzapine, clozapine);
    3. Antifungals (fluconazole, ketoconazole);
    4. Antimalarial drugs (mefloquine, chloroquine);
    5. Antidepressants (amitriptyline, imipramine, clomipramine, dutasteride, doxepin).

# 7. Safety Reports and Adverse Event Management

## 7.1 Related Definitions

AEs are any unwanted medical events that occur after a subject has received the test drug and may be manifested by signs and symptoms, illness, or abnormal laboratory tests that are not necessarily causally related to the test drug.

Definite signs or symptoms of tumor progression should not be recorded as an adverse event unless they are more severe than expected or the investigator believes that the tumour progression is related to the study drug administration or study procedures. If a new primary malignancy develops, such events are considered AEs.

Serious adverse events (SAEs) are adverse medical events such as death, life-threatening, permanent or significant disability or loss of function, the need for hospitalization or prolonged hospitalization, and congenital anomalies or birth defects that occur after a subject has received the test drug.

Suspected and Unanticipated Serious Adverse Reaction (SUSAR) means a suspected and unanticipated serious adverse reaction where the nature and severity of the clinical manifestation exceed the information available in the investigator's manual of the test drug, the specification for the marketed product, or the summary of product characteristics.

## 7.2 Severity Assessment

Investigators are required to describe the maximum severity of the adverse event using the above definition of severity in accordance with CTCAE Version 5.0. If the adverse event is determined to be a SAE, the CTCAE classification recorded on the eCRF Adverse Event Page must be consistent with the description of the NCI CTCAE classification included in the narrative section of the SAE report.

| **level** | **Clinical description of severity** |
| --- | --- |
| 1 | Mild; asymptomatic or mild; seen only for clinical or diagnostic purposes; no treatment is required. |
| 2 | Moderate; minor, localized or non-invasive treatment is required; age-appropriate instrumental activities of daily living are limited*. |
| 3 | Serious or medically significant but not immediately life-threatening; results in hospitalization or delayed hospitalization; disability; autonomic limitations in activities of daily living** |
| 4 | Life-threatening; urgent treatment is required. |
| 5 | AE-related deaths. |

Activities of Daily Living (ADL)

* Instrumental ADL refer to cooking, buying clothes, using the phone, financial management, etc.

** Autonomic ADL refer to bathing, dressing and undressing, eating, toileting, taking medication, etc., and are not bedridden.

Note the difference between severity and seriousness of an AE. A SAE may not be a SAE. For example, a headache can be severe (significantly affecting the subject's daily functioning) but is not necessarily a SAE unless it meets one of the above criteria for a SAE.

## 7.3 Causality Assessment

Investigators must assess the causes of all AEs (serious and non-serious); investigators must document causal relationships appropriately in the eCRF and report them as required for serious adverse events. The investigator's causal assessment is the investigator's assessment of the likelihood that this trial product caused or contributed to the adverse event. If the investigator's assessment of the cause of the adverse event remains unclear, the event should be reported as "related to the study drug" according to the sponsor's requirements. If the investigator's assessment is "unexplained but not related to study drug", this should also be clearly documented in the trial record.

In addition, if the investigator judges a SAE to be treatment-related, this causal relationship must be appropriately documented in the original documentation and in the eCRF, and the results of the evaluation should be reported according to the appropriate SAE reporting requirements, if necessary.

According to the five principles of adverse event analysis, the correlation evaluation is divided into definitely related, probably related, probably unrelated, definitely unrelated, and unable to evaluate.

Definitely related: the temporal sequence of administration and events is reasonable; the event stops or rapidly decreases or improves after discontinuation of the drug (depending on immune status, some ADRs may occur several days after discontinuation of the drug); the event recurs on reapplication and may be markedly exacerbated (i.e., a positive re-excitation test); the event is supported by the literature; and the influence of other confounding factors, such as the original disease, has been ruled out..

Probably related: no history of repeated use of the drug, and there is a close relationship between the use of the drug and the time of the reaction, as well as supporting documentation; however, other medications that triggered the event or progression factors of the original disease cannot be excluded.

Probably unrelated: the event is not closely correlated with the timing of the drug, the presentation of the event is not consistent with known safety information about the drug, and the progression of the original disease is equally likely to have a similar clinical presentation.

Definitely unrelated: the event is not closely related or not related to the timing of the drug, the presentation of the event is not consistent with known safety information about the medication. It is clear that the event is related to the original condition or to other medicines.

Unable to evaluate: the report has too many missing items, causality is difficult to establish, and information cannot be added.

Of these, definitely related and probably related should be considered as AEs related to the study drug.

## 7.4 Exposure During Pregnancy

Exposure during pregnancy (also known as intrauterine exposure [EIU]) is considered if the following conditions occur in the study:

- Females who received study drug and became pregnant, are found to be pregnant, or are found to be pregnant after study discontinuation (maternal exposure);
- Males who are exposed to the study drug prior to conception and/or during pregnancy due to therapeutic factors (paternal exposure).

Any subject or partner enrolled in the trial who becomes pregnant or is found to be pregnant while receiving the trial drug must be reported by the investigator on the Pregnancy Report Form and submitted to Shouyao Holdings. Report the pregnancy to Shouyao Holdings immediately upon notification of the pregnancy, whether or not an adverse event occurs in the pregnant woman or fetus.

For all pregnancy exposures where the outcome is unknown, the investigator should follow the pregnant woman until the end of pregnancy or termination of pregnancy (e.g. abortion) and report the outcome to Shouyao Holdings. The investigator should submit this information as a follow-up report on the first pregnancy report form. If the pregnancy outcome meets the criteria for a SAE, such as spontaneous abortion, stillbirth, neonatal death, or congenital anomaly (including aborted fetus, stillbirth, or neonatal death), the investigator should report it according to the procedures for reporting SAEs. If the infant is alive at birth, it should be considered 'normal' at birth (i.e. no minimum follow-up period is required for infants who are considered normal until the Pregnancy Report Form is completed). The 'normality' of the aborted f etus can be assessed by visual observation, except in cases where pre-abortion findings suggest the presence of congenital anomalies.

Additional information on pregnancy outcomes as SAEs is as follows:

- "Spontaneous abortion" includes both inevitable abortion and induced abortion.
- All neonatal deaths occurring within one month of birth, regardless of cause, should be reported as SAEs.

In addition, any infant death one month after birth should also be reported whenever the investigator believes that the death may be related to the study drug.

The investigator will request further follow-up of birth outcomes (e.g., follow-up of preterm infants to confirm the onset of developmental delay), as appropriate. In the case of paternal exposure, the investigator will provide the subject's partner with a Pregnancy Partner Information Report Form. The investigator should complete the in utero exposure form given to the subject for delivery to his/her partner.

## 7.5 Follow-up of AEs

If the subject is permanently discontinuation due to an AE or abnormal laboratory test, the subject should be followed until recovery or stabilization of the AE. If the subject has to discontinue medication for more than 4 weeks because of a trial drug-related AE, the subject should be withdrawn from the trial, but should continue to be followed for resolution of the AE until recovery or stabilization is required. If the subject is lost to follow-up, this should be recorded in the eCRF.

After the first AE is reported, the investigator is required to initiate follow-up with each subject and provide the sponsor with further information on the subject's status. All AEs for which information are recorded at the last follow-up visit/contact and that are still ongoing will continue to be evaluated at the next follow-up visit/contact. All AEs require follow-up until recovery or disease stabilization, or the event can be explained, or the subject is lost to follow-up. Once recovered, the AE information will need to be updated.

The investigator assures that follow-up visits will attempt to supplement the investigation and clarify the nature of the AE or its causality, which may require additional laboratory tests, examinations, histopathological studies, or consultation with other healthcare professionals. The sponsor may require the investigator to perform or arrange for additional investigations or assessments to elucidate the nature of the AE or its causality, and the investigator is obligated to cooperate with the sponsor.

## 7.6 Reporting Deadlines and Requirements

During the trial, the investigator should record AEs in the medical record and case report form in a true, accurate, complete, timely, and lawful manner. The investigator should evaluate each AE to determine whether it meets the criteria for a SAE. All AEs should be followed until remission or stabilization.

Except for SAEs that are not required to be reported immediately by the protocol or other documentation (e.g., investigator's manual), the investigator should report all SAEs immediately to the sponsor, followed by a detailed written follow-up report in a timely manner. For reports of death, the investigator should provide the sponsor and the Ethics Committee with other necessary information, such as autopsy reports and final medical reports.

SAEs that occur at the end of the trial, or after the end of the follow-up period until the end of the trial, should be reported by the investigator to the sponsor, and should be reported promptly if they are unexpected SAEs.

All safety-related information received by the sponsor from any source should be analyzed and evaluated promptly, including severity, relevance to the trial drug, and whether it is an expected event. The sponsor should promptly report suspected and unanticipated SAEs to all investigators involved in the trial, to the trial research center, and to the Ethics Committee; the sponsor should report suspected and unanticipated SARs to the regulatory authority and to the competent health authority.

For information on suspected and unanticipated SAEs and other potentially serious safety risks occurring during the clinical trial of drug, the sponsor shall report them in a timely manner to the Drug Evaluation Centre and other supervisory authorities prescribed by law in accordance with the relevant requirements.

The email address of the sponsor of this project to receive the SAE report is:[sae@centaurusbio.com](mailto:sae@centaurusbio.com)。

If the subject starts other anti-tumor therapy, the reporting deadline for non-serious adverse events ends at the start of the new treatment. However, if the subject dies within the safety follow-up period after the last dose of study drug, the death must be reported to the sponsor regardless of whether the subject has received other treatment.

# 8. Data Management

In this study, EDC is used for data collection.

It is the investigator's ultimate responsibility to collect and report all clinical, safety, and laboratory data in the eCRF and any other data collection forms (raw files), and to ensure the accuracy, reliability/truthfulness, attribution, completeness, consistency, clarity, timeliness (simultaneity), permanence, and accessibility of these data.

## 8.1 Data Entry and Modification

The investigator and/or authorized researchers are responsible for performing the data collection, and the data manager compiles the database and data verification procedures and performs data management. In order to ensure the accuracy of the data, the data manager sends queries about the data in this EDC to the investigator and the clinical supervisor, who should respond and return as soon as possible, and may send a further query if necessar.

## 8.2 External Data Management

The first draft of the external data transfer protocol shall be prepared by the data management unit and finalized after review and approval by the sponsor and the biospecimen testing unit. The biospecimen testing unit shall complete the external data transfer in accordance with the provisions of the external data transfer protocol, and the data manager shall perform a timely consistency check of the external data and the EDC data. If the consistency check reveals problems, this should be confirmed with the biospecimen testing unit in a timely manner and, if necessary, a resubmission of the data may be requested.

## 8.3 Data Lock

After the study team (principal investigator, Shouyao holdings, data manager, statistical analyst, etc.) confirms that the data entry is complete and the data are clean, the database will be locked by the data manager in accordance with the relevant SOPs. After locking, the database will not be changed arbitrarily; if there is any modification, it is necessary to open and lock the database according to the relevant SOPs.

## 8.4 Record Keeping

To ensure inspections and/or audits by regulatory authorities or the Shouyao holdings, the investigator should maintain records that include the identity of all subjects enrolled in the study (with sufficient information to link to the file, e.g., medical records), the original informed consent forms with all signatures, the serious adverse event form, the original documentation, a detailed record of the treatment, and sufficient documentation of relevant correspondence (e.g., letters, meeting times, telephone logs). The investigator should record the files for the longest period of time required therein, as agreed in the GCP, regulations, or the clinical research protocol.

If, for any reason, the investigator is unable to continue to keep the study records during the required retention period (e.g. retirement, job transfer), he/she should notify Shouyao holdings. At the same time, the trial records must be transferred to a designated representative recognized by TPH, e.g. another investigator, a research institution or an independent third party appointed by TPH. The investigator must obtain written permission from Shouyao holdings before disposing of any records, even if it is consistent with the need for retention.

# 9. Statistical Analysis

## 9.1 Statistical Analysis of Data Sets

**Full Analysis Set (FAS Set):** Baseline data and efficacy analyses were performed on all complete cases who had taken at least one dose of the study drug, on an intent-to-treat (ITT) basis.

**Per-Protocol Set (PPS set):** Subjects without significant protocol deviations in the FAS set. Missing data were not imputed. Drug efficacy is statistically analyzed using both FAS and PPS.

**Safety Analysis Set (SAS Set):** All enrolled subjects who used the study drug at least once and had a post-dose safety record were part of the safety analysis set. This data set is used for safety analyses.

**Pharmacokinetic Analysis Set (PKAS set):** All enrolled cases, subjects who have used the test drug at least once and have at least one sample of evaluable PK parameters. The PKAS set will be used to generate PK parameter lists as well as PK parameter analysis. When a PK sample does not meet the requirements, the PK data may be kicked out during PK parameter analysis, and the reason for the kick is indicated during the analysis.

## 9.2 Methods of Statistical Analysis

Baseline data were analyzed by FAS; All effectiveness indicators were analyzed by FAS and PPS; SAS is used for security analysis; PKAS is used for PK analysis.

**9.2.1 General Analysis**

Unless otherwise stated, the study will be summarized using the corresponding descriptive statistics according to the type of data, i.e. Mean, Standard Deviation (SD), Median, Minimum and Maximum were used for statistical description of the measurement data; Frequency and Percentage were used for the counting and hierarchical data, and Kaplan-Meier method is used for estimation of the median time and its overall 95% credible intervals in the time-to-event data.

**9.2.2 Demographic and baseline analysis**

Demographic and baseline analysis will be based on FAS. The analysis will include, but is not limited to the following:

- distribution of subjects and analyzed population;
- basic characteristics of the subject (including demographic characteristics, life history, past medical history and medication history);
- Discontinuation, dose reductions, and early withdrawals from the study and the reasons for these will be analyzed.

This will be summarized in tables and descriptive statistics.

**9.2.3 Handling of Missing Data**

Missing data will not be processed in this study except for missing dates. Time-to-event data such as PFS will be discriminated and censored times calculated according to the censoring rules. Missing dates will be processed according to the SOP of the statistical analysis unit.

**9.2.4 Security Analysis**

Security analysis will be based on descriptive statistical summaries with indicators including mean, standard deviation, median, minimum, and maximum.

Data on physical examination, vital signs, ECOG scores, ECG parameters, and laboratory tests will be itemized and changes relative to baseline will be analyzed in summary. Laboratory test results will be graded according to the CTCAE version 5.0; variables not included in the criteria will be summarized in categories above or below the range of normal values for laboratory tests.

AEss are listed in order of subject number along with their system organ classification (SOC), preferred term (PT), and severity. The number of cases and incidence of adverse events by dose group will be summarized using different categorization methods (e.g. drug-related, CTCAE ≥ grade 3, etc.). AEs in each category will be further summarized by dose group and system organ classification (SOC), preferred term (PT). SAEs will be separately tabulated and pooled for analysis.

If a subject's dose is adjusted, all AEs (drug-related or otherwise) will be summarized separately by initial dose administered and dose taken at the end of treatment.

**9.2.5 PK Analysis**

Descriptive statistics will be summarized for PK concentrations. Analyses will use geometric mean (GM), coefficient of variation (CV%) and geometric coefficient of variation (Geometric CV%, GCV%) in addition to the statistics listed in the generic analysis to summarize descriptive statistics for PK concentration data.

**9.2.6 Efficacy Analysis**

The ORR and DCR assessed by the investigators were analyzed with point and interval estimates to calculate 95% confidence intervals of the population.

For other secondary efficacy measures, including DOR and PFS, the Kaplan-Meier method is used to estimate median time and 95% confidence intervals for the population.

# 10. Quality Control and Assurance

Clinical research units must be registered with the State Drug Administration and Supervision Department; clinical wards must meet standardized requirements and ensure that resuscitation equipment is complete; each research center must strictly follow the study protocol and truthfully complete the CRF.

To ensure compliance with the requirements of the study protocol and GCP, Shouyao Holdings or its partners will conduct regular monitoring visits during the study. Supervisors will check the original documents to ensure that the information recorded in the CRF is correct. The investigator and the research center should allow direct access to the original documents by the monitors of Shouyao Holdings or its collaborators and the relevant regulatory authorities to complete the checks.

The research center will also be subject to audits by the Institutional Review Board (IRB)/Independent Ethics Committee (IEC) and/or quality assurance audits by Shouyao Holdings and/or review by the relevant regulatory authorities.

During monitoring visits and possible audits or verifications, investigators and associated personnel should ensure availability and have sufficient time to participate in the entire process.

# 11. Ethical Requirements

## 11.1 Institutional Review Board (IRB)/Independent Ethics Committee (IEC)

It Iis the investigator's responsibility to obtain approval of this protocol, protocol revisions, informed consent forms, and other relevant documents (e.g., recruitment advertisements). All documents sent to and from the IRB/IEC should be kept in the investigator's file. The original IRB/IEC approval should be submitted to Shouyao Holdings.

This revised protocol may be used prior to IRB/IEC approval only if revision of the protocol is necessary to eliminate obvious and immediately apparent harm to subjects. In such cases, the investigator must notify the IRB/IEC and Shouyao Holdings, Inc. in writing immediately after implementation.

## 11.2 Ethical Implementation in Research

The trial is conducted in accordance with legal and regulatory requirements, the General Principles for the Implementation of International Ethical Guidelines for Human Biomedical Research (International Committee for the Organization of Medical Sciences (ICMS) 2002), GCP guidelines (International Conference on Harmonization for Development (ICH) 1996) and the Declaration of Helsinki (World Medical Association (WMA) 2008). In addition, the trial will comply with the requirements of this protocol, the International Conference on Harmonization (ICH) guidelines for the management of GCP, and the regulatory and legal requirements of the research center.

## 11.3 Subject Information and Informed Consent

All parties to the study are required to maintain the confidentiality of subjects' personal information. Subject information will not be included on any forms, reports, published articles, or in any other public context, except as required by law. Subjects' names, addresses, dates of birth and other identifying information will be replaced with codes consisting of a coding system provided by First Pharmaceutical Holdings, Inc. to de-identify subjects. At the time of data transfer, Shougang Holding will maintain a high level of confidentiality of the subject information and protect the subject's personal data.

The informed consent form must comply with ICH-GCP and local regulatory and legal requirements.

The informed consent form used in this study, as well as any changes made during the course of the study, must be approved by the IRB/IEC and Shouyao Holdings prior to use.

The investigator must ensure that each subject or his legal representative is fully informed of the nature and purpose of the trial and the possible risks of participation. The investigator or his designated representative must obtain a signed informed consent form from each subject or the subject's legal representative before beginning any procedures related to the study. The investigator will retain the original signed informed consent form for each case and provide each subject with a copy of the signed informed consent form.

## 11.4 Safety Concern Reports and Critical Program/ICH GCP Violations

Shouyao Holdings should be notified promptly of any prohibition or restriction on clinical trials by a competent authority in any part of the world (e.g., suspension of a clinical trial) or if the investigator becomes aware of any new information that may affect the evaluation of the investigational product.

In addition, the investigator should immediately notify Shouyao Holdings of any immediate safety precautions taken by the investigator to protect trial subjects from immediate harm, as well as any serious violation of this study protocol or ICH GCP known to the investigator.

# 12. Sponsor's Criteria for Termination the Study

This trial may be terminated early due to regulatory decisions, changes in IRB/IEC opinions, drug safety issues and Shouyao Holdings’ considerations. In addition, Shouyao Holdings reserves the right to discontinue the development of SY-5007 at any time.

In the event of early termination or suspension of the trial, Shouyao Holdings will promptly notify the investigator. Upon notification, the investigator shall contact all subjects and the hospital pharmacy (if applicable) within the timeframe specified by Shouyao Holdings. As required by Shouyao Holdings, all study data shall be collected and all CRFs shall be completed to the extent possible.

# 13. Articles Published by Researchers

Shouyao Holdings has no objection to investigators publishing any information collected or generated by them, whether the results are favorable to the investigational drug or not. However, in order to avoid inadvertent disclosure of confidential information, the investigator is required to provide Shouyao Holdings with the full text of the original manuscript, abstract, or content to be disclosed in any other form (posters, invited or guest lectures, etc.) at least 30 days prior to submission or other forms of disclosure. If new measures are required to protect intellectual property rights, the researcher must agree to delay publication for a maximum of 60 days. If necessary, the researcher will remove any previously unpublished confidential information (other than the results of the study itself) prior to publication.

If the results are part of a multicenter trial, the investigator agrees to publish the multicenter trial results first. However, if the multicenter research article has not been submitted for publication within 12 months of completion or termination of the trial in all research centers, the investigator may publish the article separately, subject to the other requirements in this chapter.

For all research-related articles, research institutions must follow the Consensus Ethical Standards for Publication and Authorship, including Chapter II, Ethical Considerations for the Conduct and Reporting of Research, which harmonises the requirements for submission of articles to biomedical journals. The website of the Editorial Board of the International Medical Journal is http: //www.icmje.org/index.html#authorship.

The clinical research agreement between Shouyao Holdings and the research organization also includes a section on publication of research results. In the section entitled "Publication by the Investigator", it states that the terminology used should be consistent with that used in the clinical trial protocol.

# 14. References

[1] Takahashi, M.; Ritz, J.; Cooper, G.M. Activation of a novel human transforming gene, ret, by DNA rearrangement. Cell 1985, 42, 581–588.

[2] FRANK⁃RAUE K，RONDOT S，RAUE F. Molecular genetics and phenomics of RET mutations：Impact on prognosis of MTC [J]. Mol Cell Endocrinol，2010，322（1/2）：2-7.

[3] Orris, E.P.; et al. RET recognition of GDNF-GFR 1 ligand by a composite binding site promotes membrane-proximal self-association [J]. Cell Rep. 2014, 8, 1894– 1904.

[4] HOUVRAS Y. Completing the Arc：Targeted inhibition of RET in medullary thyroid cancer[J]. J Clin Oncol, 2012, 30 (2).

[5] Chi, X. et al. Ret-dependent cell rearrangements in the Wolffian duct epithelium initiate ureteric bud morphogenesis [J]. Dev. Cell 17, 199–209 (2009).

[6] Tsuzuki, T. et al. Spatial and temporal expression of the ret proto-oncogene product in embryonic, infant and adult rat tissues [J]. Oncogene 10, 191–198 (1995).

[7] De Graaff, E. et al. Differential activities of the RET tyrosine kinase receptor isoforms during mammalian embryogenesis [J]. Genes Dev. 15, 2433–2444 (2001).

[8] Airaksinen, M. S. & Saarma, M. The GDNF family: signalling, biological functions and therapeutic value[J]. Nat. Rev. Neurosci. 3, 383–394 (2002).

[9] Amiel, J.; Sproat-Emison, E.; Garcia-Barcelo, M.; Lantieri, F.; Burzynski, G.; Borrego, S.; Pelet, A.; Arnold, S.; Miao, X.; Griseri, P.; et al. Hirschsprung disease consortium. Hirschsprung disease, associated syndromes and genetics: A review. J. Med. Genet. 2008, 45, 1–14.

[10] Pierotti,M, I Bongarzone, M Borello,et al. Cytogenetics and molecular genetics of carcinomas arising from thyroid epithelial follicular cells[J]. Genes Chromosomes Cancer,1996,16:1-14.

[11] Romei, C., Ciampi, R. & Elisei, R. A comprehensive overview of the role of the RET proto-oncogene in thyroid carcinoma. Nat. Rev. Endocrinol. 12,192–202 (2016).

[12] Kohno T, Ichikawa H, Totoki Y, et al. KIF5B-RET fusions in lung adenocarcinoma[J]. Nat Med, 2012, 18(3): 375-377.

[13] Takeuchi, K. et al. RET, ROS1 and ALK fusions in lung cancer [J]. Nat. Med. 18, 378–381 (2012).

[14] Lipson, D. et al. Identification of new ALK and RET gene fusions from colorectal and lung cancer biopsies [J]. Nat Med. 18, 382–384 (2012).

[15] Donis-Keller, H. et al. Mutations in the RET proto-oncogene are associated with MEN 2A and FMTC. Hum. Mol. Genet. 2, 851–856 (1993).

[16] Mulligan, L. M. et al. Germ-line mutations of the RET proto-oncogene in multiple endocrine neoplasia type 2A [J]. Nature 363, 458–460 (1993).

[17] Hofstra, R. M. et al. A mutation in the RET proto-oncogene associated with multiple endocrine neoplasia type 2B and sporadic medullary thyroid carcinoma [J]. Nature 367, 375–376 (1994).

[18] Mulligan, L. M. RET revisited: expanding the oncogenic portfolio [J]. Nat. Rev. Cancer 14, 173-186(2014).

[19] Alexander Drilon1, Zishuo I. Hu, Gillianne G. Y. Lai and Daniel S. W. Tan, Targeting RET-driven cancers: lessons from evolving preclinical and clinical landscapes [J]. REVIEWS. 14 Nov 2017

[20] Saito, M. et al. A mouse model of KIF5B–RET fusion dependent lung tumorigenesis [J]. Carcinogenesis 35, 2452–2456 (2014).

[21] Mulligan, L. M. RET revisited: expanding the oncogenic portfolio [J]. Nat. Rev. Cancer 14, 173-186(2014).

[22] Stransky, N., Cerami, E., Schalm, S., Kim, J. L. & Lengauer, C. The landscape of kinase fusions in cancer [J]. Nat. Commun. 5, 4846 (2014).

[23] Jhiang,S, The RET proto-oncogene in human cancers[J]. Oncogene,2000,19:5590-5597.

[24] Voss RK, Feng L, Lee JE, et al. Medullary thyroid carcinoma in MEN2A:ATA moderate or high-risk RET mutations do not predict disease aggressiveness[J]. J Clin Endocrinol Metab,2017,102(8): 2807-2813.DOI:10.1210/jc.2017-00317.

[25] Bray F, Ferlay J, Soerjomataram I, et al. Global cancer statistics 2018: GLOBOCAN estimates of incidence and mortality worldwide for 36 cancers in 185 countries[J]. CA Cancer J Clin, 2018, 68(6):394-424.

[26] Rudin CM, Drilon A, Poirier JT. RET mutations in neuroendocrine tumors: including small-cell lung cancer [J]. J Thorac Oncol 2014; 9:1240-2.

[27] Cai W, Su C, Li X, et al. KIF5B-RET fusions in Chinese patients with non-small cell lung cancer[J]. Cancer, 2013,119(8): 1486-1494.

[28] Pan Fang, Ren Qinglan. Relationship between tyrosine kinase ALK, ROS1, RET fusion genes and clinical features of lung cancer[J]. Journal of Practical Cancer, 2015, 30(7): 958-960.

[29] Wang R, Hu H, Pan Y, et al. RET fusions define a unique molecular and clinicopathologic subtype of non-small-cell lung cancer[J]. J Clin Oncol, 2012, 30(35): 4352-4359.

[30] Yokota K, Sasaki H, Okuda K, et al. KIF5B/RET fusion gene in surgically-treated adenocarcinoma of the lung[J]. Oncol Rep, 2012, 28(4): 1187-1192.

[31] Lipson D, Capelletti M, Yelensky R, et al. Identification of new ALK and RET gene fusions from colorectal and lung cancer biopsies[J]. Nat Med, 2012, 18(3): 382-384.

[32] Kim JO, Lee J, Shin JY, et al. KIF5B-RET fusion gene may coincide oncogenic mutations of EGFR or KRAS gene in lung adenocarcinomas[J]. Diagn Pathol, 2015, 10: 143.

[33] Lin C, Wang S, Xie W, et al. The RET fusion gene and its correlation with demographic and clinicopathological features of nonsmall cell lung cancer: a meta-analysis[J]. Cancer Biol Ther, 2015, 16(7): 1019-1028.

[34] Lu H, Xu H, Xie F, et al. 1p/19q codeletion and RET rearrangements in small- cell lung cancer[J]. Onco Targets Ther, 2016, 9: 3571-3577.

[35] Li, A.Y.; McCusker, M.G.; Russo, A.; Scilla, K.A.; Gittens, A.; Arensmeyer, K.; Mehra, R.; Adamo, V.; Rolfo, C. RET fusions in solid tumors [J]. Cancer Treat. Rev. 2019, 81, 101911.

[36] Mizukami, T.; Shiraishi, K.; Shimada, Y.; Ogiwara, H.; Tsuta, K.; Ichikawa, H.; Sakamoto, H.; Kato, M.; Shibata, T.; Nakano, T.; et al. Molecular mechanisms underlying oncogenic RET fusion in lung adenocarcinoma [J]. J. Thorac. Oncol. 2014, 9, 622–630.

[37] Neumann HPH, Bausch B, McWhinney SR, Bender BU, Gimm O, Franke G, et al. Germ-line mutations in non-syndromic pheochromocytoma [J]. N Engl J Med 2002; 346:1459–66.

[38] Ju YS, Lee W-C, Shin J-Y, Lee S, Bleazard T, Won J-K, et al. A transforming KIF5B and RET gene fusion in lung adenocarcinoma revealed from whole-genome and transcriptome sequencing [J]. Genome Res 2012; 22:436–45.

[39] Sarfaty M, Moore A, Neiman V, Dudnik E, Ilouze M, Gottfried M, et al. RET fusion lung carcinoma: response to therapy and clinical features in a case series of 14 patients [J]. Clin Lung Cancer 2017;18: e223-32.

[40] Gautschi O, Milia J, Filleron T, Wolf J, Carbone DP, Owen D, et al. Targeting RET in patients with RET-rearranged lung cancers: results from the global. Multicenter RET Registry [J]. J Clin Oncol 2017; 35:1403-10.

[41] SONG Z, YU X, ZHANG Y. Clinicopathologic characteristics，genetic variability and therapeutic options of RET rearrangements patients in lung adenocarcinoma [J]. Lung Cancer，2016，101：16⁃21.

[42] LU C, DONG X R, ZHAO J, et al. Association of genetic and immuno-characteristics with clinical outcomes in patients with RET⁃rearranged non⁃small cell lung cancer：a retrospective mul⁃ticenter study [J]. J Hematol Oncol, 2020, 13(1):37.

[43]ZHU Y C，WANG W X，ZHANG Q X，et al. The KIF5B⁃RET fusion gene mutation as a novel mechanism of acquired EGFR tyrosine kinase inhibitor resistance in lung adenocarcinoma [J]. Clin Lung Cancer, 2019, 20(1): e73⁃73e76.

[44]OFFIN M，SOMWAR R，REKHTMAN N，et al. Acquired ALK and RET gene fusions as mechanisms of resistance to osimertinib in EGFR ⁃ mutant lung cancers [J]. JCO Precis Oncol, 2018, 2: 10.1200/P0.18.00126.

[45] Kodama T, Tsukaguchi T, Satoh Y, et al. Alectinib shows potent antitumor activity against RET- rearranged nonsmall cell lung cancer[J]. Mol Cancer Thers, 2014, 13 (12): 2910-2918.

[46] Fenton CL, Lukes Y, Nicholson D, Dinauer CA, Francis GL, Tuttle RM. The ret/PTC mutations are common in sporadic papillary thyroid carcinoma of children and young adults. J Clin Endocrinol Metab 2000; 85:1170-5.

[47] Elisei R, Romei C, Vorontsova T, Cosci B, Veremeychik V, Kuchinskaya E, et al. RET/PTC rearrangements in thyroid nodules: studies in irradiated and not irradiated, malignant and benign thyroid lesions in children and adults. J Clin Endocrinol Metab 2001; 86:3211–6.

[48] Cheung CC, Carydis B, Ezzat S, Bedard YC, Asa SL. Analysis of ret/PTC gene rearrangements refines the fine needle aspiration diagnosis of thyroid cancer. J Clin Endocrinol Metab 2001; 86:2187–90.

[49] Nikiforov YE. RET/PTC rearrangement in thyroid tumors. Endocr Pathol 2002; 13:3–16.

[50] Romei C, Elisei R. RET/PTC translocations and clinico-pathological features in human papillary thyroid carcinoma. Front Endocrinol (Lausanne) 2012; 3:54.

[51] Romei C, Fugazzola L, Puxeddu E, Frasca F, Viola D, Muzza M, et al. Modifications in the papillary thyroid cancer gene profile over the last 15 years. J Clin Endocrinol Metab 2012; 97: E1758–65.

[52] Lu Z, Zhang Y, Feng D, Sheng J, Yang W, Liu B. Targeted next generation sequencing identifies somatic mutations and gene fusions in papillary thyroid carcinoma. Oncotarget 2017;8 :45784–92.

[53] Integrated genomic characterization of papillary thyroid carcinoma. Cell 2014; 159: 676–90.

[54] Bounacer A, Wicker R, Caillou B, Cailleux AF, Sarasin A, Schlumberger M, et al. High prevalence of activating ret proto-oncogene rearrangements, in thyroid tumors from patients who had received external radiation. Oncogene 1997; 15:1263–73.

[55] Hamatani K, Eguchi H, Ito R, Mukai M, Takahashi K, Taga M, et al. RET/PTC rearrangements preferentially occurred in papillary thyroid cancer among atomic bomb survivors exposed to high radiation dose. Cancer Res 2008; 68:7176–82.

[56] Hamatani K, Eguchi H, Koyama K, Mukai M, Nakachi K. Kusunoki Y. A novel RET rearrangement (ACBD5/RET) by pericentric inversion, inv (10) (p12.1; q11.2), in papillary thyroid cancer from an atomic bomb survivor exposed to high-dose radiation. Oncol Rep 2014; 32:1809–14.

[57] Cabanillas ME,Mefadden DG,Durante C. Thyroid cancer [J]. Lancet,2016388(10061):2783-2795.

[58] Jin LX,Moley JF. Surgery for Iymph node metastases of medullary thyroid carcinoma: a review[J]. Cancer,2016,122(3):358-366.

[59] Zbuk, K. M. & Eng, C. Cancer phenomics: RET and PTEN as illustrative models [J]. Nat. Rev. Cancer 7, 35–45 (2007).

[60] Voss RK, Feng L, Lee JE, et al. Medullary thyroid carcinoma in MEN2A: ATA moderate or high-risk RET mutations RET mutations do not predict disease aggressiveness[J]. J Clin Endocrinol Metab,2017,102(8):2807-2813.

[61] Igarashi T, Okamura R, Jikuzono T, et al. An extended family with familial medullary thyroid carcinoma and Hirschsprung’s disease[J]. J Nippon Med Sch,2014,81(2):64-69.

[62] Krampitz GW, Norton JA. RET gene mutations (genotype and phenotype) of multiple endocrine neoplasia type 2 and familial medullary thyroid carcinoma[J]. Cancer,2014,120(13):1920-1931.

[63] Toledo RA, Hatakana R, Jr LD, et al. Comprehensive assessment of the disputed RET Y791F variant shows no association with medullary thyroid carcinoma susceptibility[J]. Endocr Relat Cancer,2015,22(1):65-76.

[64] Jasim S, Ying AK, Waguespack SG, et al. Multiple endocrine neoplasia type 2B with a RET proto-oncogene A883F mutation displays a more indolent form of medullary thyroid carcinoma compared with a RET M918T mutation[J]. Thyroid,2011,21(2):189-192.

[65] Kihara M, Miyauchi A, Yoshioka K, et al. Germline RET mutation carriers in Japanese patients with apparently sporadic medullary thyroid carcinoma: a single institution esperience [J]. Auris Nasus Larynx,2016,43(5):551-555.

[66] Zedenius J. Is somatic RET mutation a prognostic factor for sporadic medullary thyroid carcinoma? [J]. Nat Clin Prcat Endocinol Metab,2008,4(8):432-433.

[67] Lopezdelisle L, Pierreeugene C, Louisbrennetot C, et al. Activated ALK signals through the ERK-ETV5-RET pathway to drive neuroblastoma oncogenesis[J]. Oncogene,2018,37(11):1417-1429.

[68] Chen Z, Zhao Y, Yu Y, et al. Small molecule inhibitor regorafenib inhibits RET signaling in neuroblastoma cells and effectively suppresses tumor growthin vivo[J]. Oncotarget,2017,8(61):104090-104103.

[69] Krawczyk A, Hasselazar K, Pawlaczek A, et al. Germinal mutations of RET,SDHB,SDHD,and VHL genes in patients with apparently sporadic pheochromocytomas and paragangliomas[J]. Endokrynologia Polska,2010,61(1):43-48.

[70] Wang Y, Lai X, Huang L, et al. Establishment of an induced pluripotent stem cell model of Hirschsrpung disease, a congenital condition of the enteric nervous system, from a patient carrying a novel RET mutation[J]. Neuroreport,2018,29(12):975-980.

[71] Ogino H, Yano S, Kakiuchi S, Yamada T, Ikuta K, Nakataki E, et al. Novel dual targeting strategy with vandetanib induces tumor cell apoptosis and inhibits angiogenesis in malignant pleural mesothelioma cells expressing RET oncogenic rearrangement [J]. Cancer Lett 2008; 265:55–66.

[72] Le Rolle A-F, Klempner SJ, Garrett CR, Seery T, Sanford EM, Balasubramanian S, et al. Identification and characterization of RET fusions in advanced colorectal cancer [J]. Oncotarget 2015; 6:28929–37.

[73] Kim SY, Oh SO, Kim K, Lee J, Kang S, Kim K-M, et al. NCOA4-RET fusion in colorectal cancer: therapeutic challenge using patient-derived tumor cell lines. J Cancer 2018; 9:3032–7.

[74] Paratala BS, Chung JH, Williams CB, Yilmazel B, Petrosky W, Williams K, et al. RET rearrangements are actionable alterations in breast cancer [J]. Nat Commun 2018; 9:4821.

[75] Hao Yanzhi. Vandetanib—the first drug for the treatment of medullary thyroid cancer[J]. Chinese Pharmaceutical Journal, 2013, 48(14): 1229-1230

[76] Wells SA Jr, Robinson BG, Gagel RF, et al. Vandetanib in patients with locally advanced or metastatic medullary thyroid cancer: a randomized, double-blind phase Ⅲ trial [J]. J Clin Oncol, 2012, 30(2): 134-141.

[77] Brose MS, Nutting CM, Jarzab B, et al. Sorafenib in radioactive iodine-refractory, locally advanced or metastatic differentiated thyroid cancer: a randomised, double-blind, phase 3 trial [J]. Lancet, 2014, 384(9940): 319-328.

[81] Kodama T, Tsukaguchi T, Satoh Y, et al. Alectinib shows potent antitumor activity against RET- rearranged nonsmall cell lung cancer[J]. Mol Cancer Thers, 2014, 13 (12): 2910-2918.

[82] NAVIS AC，BOURGONJE A，WESSELING P，et al． Effects of dual targeting of tumor cells and stroma in human glioblastoma xenografts with a tyrosine kinase inhibitor against c-MET and VEGFR2[J]. PLoS One，2013，8( 3) : e58262．

[83] Drilon A, Wang L, Hasanovic A, et al. Response to cabozantinib in patients with RET fusion-positive lung adenocarcinomas[J]. Cancer Discov, 2013, 3(6): 630-635.

[84] Mukhopadhyay S, Pennell NA, Ali SM, et al. RET- rearranged lung adenocarcinomas with lymphangitic spread，psammoma bodies, and clinical responses to cabozantinib[J]. J Thorac Oncol, 2014, 9(11): 1714-1719.

[85] FDA. NDA 203756 approval letter [EB/OL]. (2012 -11 -29)

[86] FDA. NDA 208692 approval letter [EB/OL]. (2016 - 04 -25).

[87] FDA. FDA grants regular approval to cabometyx for first-line treatment of advanced renal cell carcinoma [EB /OL]. (2017-12-19).

[88] YAKES FM, CHEN J, TAN J, et al. Cabozantinib (XL184), a Novel MET and VEGFR2 inhibitor，simultaneously suppresses metastasis，angiogenesis,and tumor growth[J]. Mol Cancer Ther,2011,10( 12) : 2298-2308．

[89] ELISEI R, SCHLUMBERGER MJ, M LLER SP, et al. Cabozantinib in progressive medullary thyroid cancer[J]. J Clin Oncol,2013,31(29)3639-3646．

[90] SCHLUMBERGER M, ELISEI R, M LLER S, et al. Overall survival analysis of EXAM, a phase III trial of cabozantinib in patients with radiographically progressive medullary thyroid carcinoma[J]．Ann Oncol,2017,28(11) : 2813 -2819．

[91] Ping G, Hui-Min W, Wei-Min W, et al. Sunitinib in pretreated advanced non-small-cell lung carcinoma: a primary result from Asian population[J]. Med Oncol, 2011, 28(2): 578-583.

[92] Wu H, Shih JY, Yang JC. Rapid response to sunitinib in a patient with lung adenocarcinoma harboring KIF5B- RET fusion gene[J]. J Thorac Oncol, 2015, 10(9): e95-e96.

[93] Velcheti V, Hida T, Reckamp KL, et al. Phase 2 study of lenvatinib in patients with RET fusion-positive adenocarcinoma of the lung[J]. Eur J Cancer, 2017, 72(1): S178.

[94]] Kodama T, Tsukaguchi T, Satoh Y, et al. Alectinib shows potent antitumor activity against RET- rearranged nonsmall cell lung cancer[J]. Mol Cancer Thers, 2014, 13(12): 2910-2918.

1. Pietrantonio F, Di Nicolantonio F, Schrock AB, et al. RET fusions in a small subset of advanced colorectal cancers at risk of being neglected[J]. Ann Oncol, 2018, 29(6): 1394-1401.

[96] Drilon A，Oxnard G，Wirth L，et al. Registrational Results of LIBRETTO-001: A Phase 1/2 Trial of LOXO-292 in Patients with RET Fusion-Positive Lung Cancers[J]. Journal of Thoracic Oncology,2019,14(10): S6-S7.

[97] Wirth L, Sherman E, Drilon A, et al. Registrational results of LOXO-292 in patients with RET-altered thyroid cancers[J]. Annals of Oncology,2019,30(suppl 5): v933.

[98] Subbiah V, Gainor J F, Rahal R, et al. Precision Targeted Therapy with BLU-667 for RET-Driven Cancers[J]. Cancer Discovery,2018,8(7):836-849.

[99] Abdel-Magid AF. RET kinase inhibitors may treat cancer and gastrointestinal disorders. ACS Med Chem Lett 2014; 6:13-4.

[100] Redaelli S, Plaza-Menacho I, Mologni L. Novel targeted therapeutics for MEN2. Endocr Relat Cancer 2018; 25: T53-68.

.

# 15. Appendix

## 15.1 ECOG Physical Fitness Score

**Table 1-1 ECOG physical condition score**

| **Mark** | **Physical condition** |
| --- | --- |
| **0 points** | Fully normal mobility with unrestricted access to all daily activities performed in the disease-free state. |
| **1 point** | Restricted in strenuous activities, but can walk freely and engage in light physical activities or undertake sedentary work, such as light housework and office work. |
| **2 point** | Free walking and complete self-care, but incapable of work, can get up and move around more than half of the time during the day. |
| **3 point** | Can only partially take care of themselves, and are bedridden or wheelchair-bound for more than half of the daytime. |
| **4 point** | Completely incapacitated, unable to take care of themselves and completely bedridden or wheelchair |
| **5 point** | Death |

## 15.2 Efficacy Evaluation Criteria for Solid Tumors Version 1.1 (RECIST V1.1).

The following is an excerpt from the RECIST V1.1 standard.

**Definition**

At baseline, tumor lesions/lymph nodes will be classified as measurable and non-measurable by the following definitions:

**Measurable lesions**

Tumor lesions: at least one accurately measurable diameter line (recorded as the maximum diameter) with the following minimum lengths:

- CT scan 10 mm (CT scan layer thickness not greater than 5 mm)
- Clinical Routine clinical examination instruments 10 mm (tumor lesions that cannot be accurately measured by a calibrating instrument should be recorded as non-measurable)
- Chest X-ray 20 mm
- Malignant lymph nodes: pathologically enlarged and measurable, individual lymph node CT scan short diameter must be ≥ 15 mm (CT scan layer thickness recommended no more than 5 mm). At baseline and follow-up, only the short diameter is measured and followed up.

**Non-measurable lesions**

All other lesions, including small lesions (longest diameter <10 mm or pathological lymph nodes with short diameters ≥10 mm to <15 mm) and non-measurable lesions. Non-measurable lesions included: meningeal disease, ascites, pleural or pericardial effusion, inflammatory breast cancer, carcinomatous lymphadenopathy of the skin/lungs, abdominal masses that could not be diagnosed and followed up on imaging, and cystic lesions.

**Special considerations on lesion measurement**

Bone lesions, cystic lesions and lesions previously treated locally require special mention:

Bone lesions:

- Bone scans, PET scans or plain films are not suitable for measuring bone lesions but can be used to confirm the presence or absence of bone lesions;
- Osteolytic lesions or mixed osteolytic/osteogenic lesions with a defined soft tissue component that meets the above definition of measurability may be treated as measurable lesions if these lesions can be evaluated with tomographic imaging techniques such as CT or MRI;
- Osteogenesis is non-measurable lesins.

**Cystic lesions:**

- A lesion that meets the radiographic criteria for the definition of a simple cyst should not be considered malignant because it is a simple cyst by definition and is neither a measurable nor non-measurable lesion;
- If it is a cystic metastatic lesion and meets the above definition of measurability, it may be considered to be a measurable lesion. However, if a non-cystic lesion is present in the same patient, the non-cystic lesion should be preferred as the target lesion.

**Locally treated lesions:**

- Lesions located at sites that have been treated with radiotherapy or other localised regional treatments are generally treated as non-measurable lesions unless there is definite progression of that lesion.

**Description of the measurement method**

**Lesion measurement**

All tumor measurements should be recorded in metric metres at the time of clinical evaluation. All baseline ratings of tumor lesion size should be completed as close to the start of treatment as possible and must be completed within 28 days (4 weeks) prior to the start of treatment.

**Evaluation methodology**

The same techniques and methods should be used for baseline evaluation of lesions and subsequent measurements. All lesions must be evaluated using imaging, except for lesions that cannot be evaluated with imaging but only with clinical examination.

**Clinical lesions**: Clinical lesions are only considered measurable if they are located superficially and have a diameter of ≥10 mm when measured (e.g., skin nodules, etc.). For subjects with skin lesions, it is recommended that color photographs containing scale measurements of the size of the lesion be used for archiving purposes. When lesions are evaluated using both imaging and clinical examination, imaging should be used whenever possible because it is more objective and can be reviewed repeatedly at the end of the study.

**Chest x-ray**: When tumor progression is an important study endpoint, chest CT should be used in preference to X-ray because it is more sensitive than X-ray, especially for new lesions. Chest X-ray testing is only indicated when the measured lesion is well defined and the lungs are well ventilated.

**CT, MRI:** CT is currently the best available reproducible method for efficacy evaluation. The definition of measurability in this guideline is based on a CT scan layer thickness of ≤5 mm. If the CT layer thickness is greater than 5 mm, the minimum measurable lesion should be two times the layer thickness MRI is also acceptable in some cases (e.g., whole-body scan).

**Ultrasound:** Ultrasound should not be used as a measurement method for lesion size. Ultrasound is not reproducible at the end of the measurement due to its operational dependency and does not guarantee homogeneity of technique and measurement between measurements. If a new lesion is detected using ultrasound during the trial, it should be confirmed using CT or MRI. MRI can be used instead if the radiation exposure of CT is taken into account.

**Endoscopy, laparoscopy:** The use of these techniques is not recommended for the objective evaluation of tumours, but this method can be used to confirm CR in the case of biopsy specimens obtained, and also in trials where the study endpoint is recurrence or surgical resection after CR.

**Tumor markers**: Tumor markers cannot be used alone to evaluate objective tumor remission. However, if the marker level exceeds the upper limit of normal at baseline, it must return to normal when used to evaluate complete remission. Because tumur markers vary from disease to disease, this needs to be taken into account when writing measurement criteria into the protocol. Specific criteria for CA-125 remission (recurrent ovarian cancer) and PSA (recurrent prostate cancer) remission have been published. The International Organisation for Gynecological Cancer has developed criteria for CA-125 progression, which will soon be added to the objective tumor assessment criteria for first-line treatment regimens for ovarian cancer.

**Cytology/histology techniques**: These techniques can be used to identify PRs and CRs in specific circumstances specified by the protocol (e.g. residual benign tumor tissue is often present in the lesions of germ cell tumors). When exudation may be a potential side effect of a therapy (e.g. treatment with paclitaxel compounds or angiogenesis inhibitors) and the measurable tumor meets the criteria for remission or disease stabilisation, the appearance or exacerbation of tumor-associated exudation during the course of treatment may be confirmed by cytological techniques to differentiate between remission (or disease stabilisation) and disease progression.

**Assessment of tumor remission**

**Evaluation of all tumors and measurable lesions**

In order to evaluate objective remission or possible future progression, it is necessary to assess the total tumor load of all tumor lesions at baseline, as a reference for later measurements. In clinical protocols with objective remission as the primary treatment endpoint, only subjects with measurable lesions at baseline are enrolled. Measurable lesions are defined as the presence of at least one measurable lesion. In contrast, for trials in which disease progression (time to disease progression or degree of progression at a fixed date) is the primary treatment endpoint, it must be made clear in the protocol entry criteria whether enrolment is limited to subjects with measurable lesions or whether enrolment is possible without measurable lesions.

**Baseline recording of target and non-measurable lesions**

When more than one measurable lesion is present at the time of the baseline assessment, all lesions, up to a total of five (no more than two per organ), should be recorded and measured as target lesions representative of all involved organs (i.e., a maximum of two or four target lesions should be selected as baseline measurement lesions for subjects with only one or two cumulative organs).

Target lesions must be selected based on size (longest diameter), be representative of all involved organs, and measurements must be well reproducible. Sometimes the largest lesion can be re-selected as the largest repeatable lesion when the largest lesion is not repeatable.

Lymph nodes require special attention because they are normal tissue and can be detected on imaging even in the absence of tumor metastases. Pathological lymph nodes that are defined as measurable nodes or even target lesions must meet the following criteria: short diameter ≥15 mm on CT; only the short diameter needs to be detected at baseline. The radiologist usually relies on the short diameter of the node to determine whether the node has metastatic tumor. Nodal dimensions are generally expressed as two-dimensional data from imaging (CT in the axial plane, MRI in one of the axial, sagittal or coronal planes). The smallest value is taken as the short diameter. For example, a 20 mm × 30 mm abdominal nodule with a short diameter of 20 mm would be considered a malignant, measurable nodule. In this example, 20 mm is the measurement of the nodule. Nodules ≥10 mm but <15 mm in diameter should not be considered target lesions. Nodules <10 mm do not fall into the category of pathological nodules and do not need to be documented and further observed.

The calculated sum of the diameters of all target lesions (including the longest diameter for non-nodal lesions and the short diameter for nodal lesions) will be reported as the sum of the baseline diameters. If lymph node diameters are included, only the short diameters will be counted as mentioned above. The sum of the baseline diameters will be used as a reference value for the baseline level of disease.

All remaining lesions, including pathological lymph nodes, can be considered as non-target lesions and do not need to be measured, but should be recorded at the baseline assessment. They should be recorded as "present", "absent" or in rare cases "clearly progressive". Widespread target foci may be documented together with target organs (e.g., massively enlarged pelvic lymph nodes or large liver metastases).

**Mitigation criteria**

**Target lesion assessment**

Complete response (CR): disappearance of all target lesions and the short diameter of all pathological lymph nodes (both target and non-target nodes) must be reduced to <10 mm.r.

Partial response (PR): the sum of the diameters of target lesions is reduced by at least 30% from the baseline.

Disease progression (PD): a relative increase in the sum of the diameters of the target lesions by at least 20%, taking as reference the smallest value of the sum of the diameters of all the target lesions measured throughout the course of the experimental study (or the baseline value if the baseline measurement is the smallest); in addition to this, an increase in the absolute value of the sum of the diameters of the diameters of the target lesions by at least 5 mm must be fulfilled (the appearance of one or more new lesions is also considered to be disease progression).

Disease stabilization (SD): the target lesion has not decreased to the extent of PR, nor has it increased to the level of PD, and in between, the smallest value of the sum of diameters can be used as a reference for the study.

**Considerations for target disease assessment**

Lymph nodes: even if a lymph node identified as a target lesion is reduced to less than 10 mm, the value of the actual short diameter corresponding to the baseline (in the same anatomical plane as at the time of the baseline measurement) should be recorded at each measurement. This means that if a lymph node is a target lesion, even if the criteria for complete remission are met, the lesion cannot be said to have disappeared completely, because the short diameter of a normal lymph node is defined as <10 mm. Target lymph node lesions need to be recorded exclusively at specific locations on the eCRF form or in other recording modalities: for CR, the short diameters of all lymph nodes must be <10 mm, and for PR, SD, and PD, the actual measured value of the short diameters of target lymph nodes will be included in the eCRF form. For PR, SD and PD, the actual measurement of the target lymph node short diameter will be included in the sum of the target lesion diameters.

Target lesions that are too small to measure: In clinical trials, all lesions (nodular or non-nodular) that are measured at baseline should have their actual measurements recorded at subsequent assessments, even if they are very small (e.g. 2 mm). However, sometimes they may be so small that the image on the CT scan is very blurred and it is difficult for the radiologist to define the exact value, and they may be reported as "too small to measure". If this happens, it is important to record a value in the eCRF table. If the radiologist thinks the lesion may have disappeared, then this should also be recorded as 0 mm, and if the lesion is present but vague and no precise measurement can be given, then the default value is 5 mm. (Note: This is unlikely to be the case for lymph nodes, which usually have measurable dimensions or, as in the retroperitoneal space, are often surrounded by fatty tissue; however, if this is also the case, it is important to record a value on the eCRF chart. The default value of 5 mm is derived from the slice thickness of the CT scan (this value does not change depending on the slice thickness value of the CT). Providing this default value reduces the risk of incorrect assessment, as the likelihood of repeating the same measurement is low. However, it is important to remember that if the radiologist can give an accurate value for the size of the lesion, the actual value must be recorded, even if the lesion diameter is less than 5 mm.

Separated or bound lesions: If a non-nodular lesion splits into fragments, the longest diameters of the separated parts are added together to calculate the sum of the diameters of the lesions. Similarly, in the case of joined lesions, they can be distinguished by the planes between the joined parts and their respective maximum diameters are then calculated. However, if the union is inseparable, the longest diameter should be taken as the longest diameter of the united lesion as a whole.

**Assessment of non-target lesions**

Complete response (CR): disappearance of all non-target lesions and return of tumour markers to normal levels. All lymph nodes are non-pathological in size (short diameter <10 mm).

Non-complete remission/non-progression of disease: presence of one or more non-target lesions and/or persistence of tumor marker levels above normal.

Disease progression (PD): definite progression of pre-existing non-target lesions. Note: The presence of one or more new lesions is also considered disease progression.

**Special considerations for the assessment of progression of non-target lesions**

An additional explanation of the definition of progression of non-target lesions is as follows: if measurable non-target lesions are present in a subject, even if the target lesion is judged to be stable or in partial remission, a definitive definition of progression based on the non-target lesions must be met with an overall deterioration of the non-target lesions to the extent that treatment must be discontinued. A general increase in the size of one or more non-target lesions is often insufficient to meet the criteria for progression, and therefore it is rarely possible to define overall tumor progression based on changes in non-target lesions alone when the target lesion is stable or in partial remission.

**New lesions**

The appearance of new malignant lesions signals disease progression; therefore, some assessment of new lesions is important. There are no specific criteria for detecting lesions on imaging, but the detection of a new lesion should be unequivocal. For example, progression cannot be attributed to a difference in imaging technique, a change in imaging morphology, or a lesion other than the tumor (for example, some so-called new bone lesions are simply a healing of the original lesion or a recurrence of the original lesion). This is important when a patient has a partial or complete response to a baseline lesion, e.g. a case of necrosis in a liver lesion may be reported as a new cystic lesion on the CT report when it is not.

Lesions detected at follow-up but not at baseline are considered new and indicative of disease progression. For example, a subject with a visceral lesion detected at baseline who has a metastatic lesion detected at a cranial CT or MRI scan will be considered to have an intracranial metastatic lesion, even if they did not have a cranial scan at baseline.

If a new lesion is poorly defined, for example because of its small morphology, further treatment and follow-up is required to confirm that it is a new lesion. If the repeat examination confirms that it is a new lesion, the time of disease progression should be counted from the time of its initial discovery.

The assessment of lesions by FDG-PET generally requires additional tests for additional confirmation, and a combination of FDG-PET and additional CT findings is useful for assessing progression (especially in new suspected disease). New lesions that can be clarified by FDG-PET are assessed according to the following procedure:

A negative baseline FDG-PET followed by a positive follow-up FDG-PET indicates disease progression.

No baseline FDG-PET is performed and the subsequent follow-up FDG-PET is positive:

Disease progression is demonstrated if the positive follow-up FDG-PET shows new lesion foci consistent with the CT findings.

If the positive findings on follow-up FDG PET show a new lesion that is not confirmed by the CT findings, a further CT scan is required to confirm it (if confirmed, the time of disease progression is counted from the time the abnormality is detected on the previous FDG PET scan).

Disease progression does not occur if the positive findings on follow-up FDG-PET are consistent with a pre-existing lesion detected on CT and the lesion does not progress on imaging.

**Best overall efficacy assessment**

The best overall efficacy assessment is efficacy from the start of the trial to the end of the trial, taking into account any necessary confirmatory conditions. Sometimes the efficacy response occurs after the end of treatment, so the protocol should make it clear whether the end-of-treatment efficacy assessment is included in the best overall efficacy assessment. The protocol must make clear how any new treatment before progression affects the best efficacy response. The best efficacy response in a subject is largely dependent on the outcome of the target and non-target lesions and how well the new lesions perform. It also depends on the type of trial, protocol requirements and outcome measures. In particular, in non-randomised trials, the efficacy response profile is the primary objective and confirmation of PR or CR is required to confirm which is the best overall efficacy assessment.

***Point-in-time reaction***

It is assumed that efficacy evaluations will be available at specific time points in each protocol. Table 1 will provide a summary of the overall efficacy evaluations at each time point for a population of subjects with measurable disease at baseline level.

If the subject has no measurable disease (no target lesion), the evaluation can be found in Table 2.

***Statements of absence and non-evaluation of assessments***

If a lesion could not be imaged or measured at a particular time point, the subject is not evaluable at that time point. If only part of a lesion can be assessed at a given time point, this will usually be considered as not evaluable at that time point, unless there is evidence to confirm that the missing lesion does not affect the assessment of efficacy response at the given time point. This situation is likely to occur in the case of disease progression. For example, a subject who has 3 lesions totalling 50 mm at baseline, but then has only 2 evaluable lesions totalling 80 mm, will be assessed for disease progression regardless of the impact of the missing lesions.

**Best overall response: All time points**

Once all data were available for participants, the best overall response could be determined.

Assessment of best overall response when the trial does not require confirmation of a complete or partial efficacy response: the optimal efficacy response in a trial is the best response at all time points (e.g. a subject whose efficacy is evaluated as SD in cycle 1, PR in cycle 2, and PD in cycle 1, but whose optimal complete remission is evaluated as PR). If the optimal complete remission is evaluated as SD, the optimal complete remission must be achieved as specified in the protocol. minimum time from baseline. If the minimum time criterion is not met, even a Best Total Remission evaluation of SD will not be accepted and the subject's Best Total Remission will be subject to subsequent evaluations. For example, a subject who is evaluated as SD in Cycle 1 and PD in Cycle 2, but does not meet the minimum time requirement for SD, would have a Best Total Remission evaluation of PD. The same subject who misses a visit after being evaluated as SD in Cycle 1 would be considered non-evaluable.

Assessment of best overall response when the trial requires confirmation of a complete or partial response: a complete or partial remission can only be declared if each subject meets the trial-specified criteria for a partial or complete remission and, as explicitly stated in the protocol, efficacy is confirmed at a later time (usually after four weeks). In this case, the optimal complete remission is described in Table 3.

Table 1 Time-point efficacy: subjects with target lesions (including or excluding non-target lesions)

| Target lesions | Non-target lesions | New lesions | Total mitigation |
| --- | --- | --- | --- |
| CR | CR | No | CR |
| CR | Non-CR/non-PD | No | PR |
| CR | Cannot be evaluated | No | PR |
| PR | Non-progressive or cannot be fully evaluated | No | PR |
| SD | Non-progressive or cannot be fully evaluated | No | SD |
| Cannot be fully evaluated | Non-progressive | No | NOT |
| PD | Any situation | Yes or no | PD |
| Any situation | PD | Yes or no | PD |
| Any situation | Any situation | Yes | PD |
| CR = complete response | PR = partial mitigation | SD = disease stable | PD = disease progression NE = not assessable |

Table 2 Time-point efficacy - subjects with only non-target lesions

| Non-target lesions | New lesions | Total mitigation |
| --- | --- | --- |
| CR | No | CR |
| Non-CR or non-PD | No | Non-CR or non-PD |
| Cannot be fully evaluated | No | Cannot be evaluated |
| PD cannot be fully evaluated | Yes or no | PD |
| Any situation | Yes | PD |

Note: For non-target lesions, "non-CR/non-PD" refers to efficacy that is superior to SD. As SD is increasingly used as an endpoint indicator for evaluating efficacy, non-CR/non-PD efficacy is developed to target situations where no lesions were specified to be measurable.

For unspecified progressive findings (e.g., very small indeterminate new lesions; cystic or necrotic lesions in pre-existing lesions) therapy may be continued until the next assessment. If disease progression is confirmed at the next assessment, the date of progression should be the date of the previous suspected progression.

Table 3 Efficacy of CR and PR requires confirmation of best overall response

| First time point total relief | Subsequent time point total relief | Best overall response |
| --- | --- | --- |
| CR | CR | CR |
| CR | PR | SD, PD or PR^a^ |
| CR | SD | SD if it lasts enough time, otherwise PD |
| CR | PD | SD if it lasts enough time, otherwise PD |
| CR | NOT | SD if it lasts enough time, otherwise NE |
| PR | CR | PR |
| PR | PR | PR |
| PR | SD | SD |
| PR | PD | SD if it lasts enough time, otherwise PD |
| PR | NE | SD if it lasts enough time, otherwise NE |
| NE | NE | NE |

Note: CR is complete remission, PR is partial remission, SD is stable disease, PD is progressive disease, and NE is not evaluable. Superscript "a": if there is a true CR at the first time point and any disease appears at a subsequent time point, then even if the subject achieves PR relative to baseline, the efficacy rating will still be PD at the subsequent time point (because disease will reappear after the CR). Best overall response depends on whether SD occurs within the shortest treatment interval; however, sometimes the first evaluation is a CR, but subsequent time-point scans suggest that small lesions still appear to be present, so that in fact the subject's outcome should be a PR at the first time-point rather than a CR. In this case, the first CR judgement should be modified to a PR, with the best response being a PR.

## 15.3 Creatinine Clearance Calculation Formula

According to the Cockcroft Gault formula:

male:
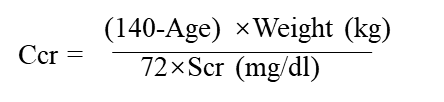
 or
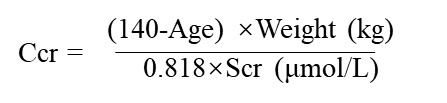


Female:
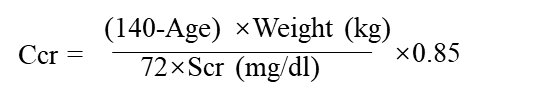
 or
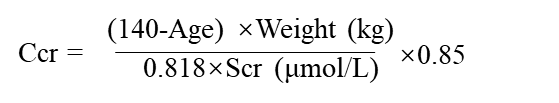


## 15.4 Examples of abstinence or contraception

Examples of cyclic abstinence include: ovulation, temperature or post-ovulation methods.

Examples of non-hormonal contraceptive methods with an annual failure rate of <1% include: tubal ligation, male sterilisation, intrauterine devices, etc. Alternatively, a combination of two methods may be used to achieve an annual failure rate of <1%.

## 15.5 New York Heart Association Cardiac Function Classification (NYHA)

| **level** | **Physical activity** | **Resting state** | **Symptoms (fatigue, palpitations, wheezing, or angina)** |
| --- | --- | --- | --- |
| I | Unrestricted | Asymptomatic | General physical activity does not cause |
| II | Mildly restricted | Asymptomatic | Daily physical activity can cause |
| III | Significantly restricted | Asymptomatic | Caused by lower than daily physical activity |
| IV | forfeit | Symptomatic | Any physical activity can cause |

## 15.6 Program Amendment Records

**Scenario remediation records**

| **Version number** | **Revised chapters** | **Amendments** | **Main reasons** | **Amendmentist** |
| --- | --- | --- | --- | --- |
| V1.0 | not applicable | not applicable | not applicable | not applicable |
|  |  |  |  |  |
|  |  |  |  |  |
|  |  |  |  |  |
|  |  |  |  |  |
|  |  |  |  |  |
|  |  |  |  |  |
|  |  |  |  |  |
|  |  |  |  |  |
|  |  |  |  |  |
|  |  |  |  |  |
|  |  |  |  |  |
|  |  |  |  |  |
|  |  |  |  |  |
|  |  |  |  |  |

## 15.7 Risk Prevention and Control Program

Based on the important known risks, important potential safety risks, important missing data and safety data of similar products, the following risk control plan has been developed:SY-5007 Possible Safety Risks and Risk Control Plan.

| Significantt known risks: | | | | |
| --- | --- | --- | --- | --- |
| This product is the first time it has been applied to human studies. | | | | |
| Important potential risks | | | | |
| Risk issues |  | Processing principles and instructions | | |
|  | Precautions | Monitors | Clarification | Combination therapy |
| Blood system | 1. The Protocol entry criteria stipulate that subjects must meet the following criteria:   Bone marrow function:  • absolute neutrophil (ANC) ≥1.5×10^9^/L; • platelets≥ 75×10^9^/L; • Hemoglobin ≥ 85 g/L.   The Adverse effects of prior antineoplastic therapy have not recovered to a CTCAE 5.0 grade rating of ≤ grade 1 (except for toxicities judged by the investigator to pose no safety risk, e.g. alopecia, grade 2 peripheral neurotoxicity, etc.);  2. DLT is defined as the following toxic reactions that have a correlation (including definitely related and possibly related) to the study drug occurring within a single dose and within cycle 1 (35 days) of continuous dosing:  (1) Hematological toxicity:   Grade 4 neutropenia lasts > 7 days;   Grade ≥3 febrile neutropenia;   Grade 4 thrombocytopenia;   Grade 3 thrombocytopenia with bleeding;   Grade 4 anemia. |  The protocol provides for routine blood tests, blood biochemistry and coagulation function during the screening period; routine blood tests, blood biochemistry and blood biochemistry are performed at weeks 1, 2 and 5 of cycle 1, and every eight weeks starting from cycle 2 of the study treatment period. Additional coagulation function tests when clinical indications or suspected symptoms occur.  **Dose adjustment:**  Treatment of hematological toxicity: in the event of hematological toxicity, the investigator may provide symptomatic treatment based on clinical manifestations, etc. If the hematological toxicity reaches grade ≥3, suspend dosing and take the drug at the original dose level (when the original dose is the lowest dose) or reduce the dose level by 1 dose level after resumption of dosing to grade <3. If Grade ≥3 hematological toxicity reoccurs after resumption of dosing, subjects are discontinued and withdrawn from the study. If Grade 3 or 4 anemia develops, the dose may not be suspended and, at the investigator's discretion, may be treated with a blood transfusion. |  Some hematological toxicities (changes in blood cell counts, coagulation indices, etc.) identified in the preclinical SD rat repeat dosing trials of this product are related to adverse events associated with drugs with a similar mechanism of action to that of this product, and are therefore subject to the relevant regulations.   The current protocol has been described more fully. | When toxicity occurs, the investigator can carry out symptomatic treatment according to clinical manifestations. |
| Electrolyte abnormalities | NA | The protocol provides for blood biochemistry during the screening period; blood biochemistry is performed weekly during cycle 1 and every eight weeks beginning with cycle 2 of the study treatment period. |  Abnormalities such as blood PK were found in the preclinical SD rat and Beagle repeated administration test of this product, and the relevant regulations were carried out in consideration of the fact that similar mechanism of action drugs have been reported. | Treatment of hyperphosphatemia: restriction of phosphorus intake; dialysis treatment: high-throughput dialysis is more effective in removing blood phosphorus; use of phosphorus binding agents: traditional phosphorus binding agents include aluminium- and calcium-containing phosphorus binding agents, and newer ones include sevelamer, lanthanum carbonate, nicotinic acid (niacinamide), iron-containing phosphorus binding agents, and colestiline. Surgery may be indicated if necessary.  Treatment of hyperkalaemia: furosemide, hydrochlorothiazide, sodium bicarbonate, calcium gluconate, diethylstilbestrol, pyridostigmine hydrochloride, polyurethane resins, polysulfonated styrene, dextrose, haemodialysis filtration. |
| Liver and gallbladder | 1. The protocol entry criteria stipulate that subjects must meet the following criteria:   liver function:  Serum albumin transaminase (AST), serum albumin transaminase (ALT) ≤ 3 times the upper limit of normal (ULN), total serum bilirubin (TBIL) ≤ 1.5 times the ULN.  2. The Protocol entry criteria stipulate that subjects must meet the following criteria:   active hepatitis (Hepatitis B: HbsAg positive and HBV-DNA ≥ 2000 IU/ml; Hepatitis C: HCV antibody positive and HCV-RNA ≥ 1000 IU/ml).  3. DLT is defined as the following toxic reactions related (including definitely related and possibly related) to the study drug occurring within a single dose and within the 1st cycle (35 days) of consecutive dosing:  (1) Non-hematological toxicity:   Grade 4 non-hematological toxicity;   Grade 3 non-hematological toxicity that does not recover to ≤ Grade 2 within 3 days of treatment (except for simple laboratory test abnormalities that, in the judgement of the investigator, are not clinically symptomatic and do not require intervention).  (2) Other toxic reactions that, in the judgement of the investigator, require permanent discontinuation of the study drug or result in discontinuation of the first cycle for more than 7 days. |  The protocol provides for blood biochemistry during the screening period; blood biochemistry is performed weekly during cycle 1 and every eight weeks beginning with cycle 2 of the study treatment period.  **Dose adjustment:**  Hepatoprotective therapy: subjects were treated with hepatoprotective therapy when any of ALT, AST, ALP, GGT, and bilirubin were Grade 1. If the total bilirubin reaches grade ≥2 after hepatoprotective treatment, the drug should be suspended, and the drug should be lowered by one dose level after recovering to grade ≤1; if any one of the above indexes except the total bilirubin reaches grade ≥3 after hepatoprotective treatment, the drug should be suspended, and the drug should be lowered by one dose level after recovering to grade ≤1, and if the relapse occurs again, the subject should be withdrawn from the study. |  Provision is made for the toxicity to the liver and gallbladder found in preclinical animal long term toxicity tests, and for adverse events associated with drugs with a similar mechanism of action to this product. | Hepatoprotective therapy may be selected but not limited to: polyphosphatidylcholine, glucuronolactone, glutathione, thiopronin, glycyrrhizateamine, glycyrrhizinatediamine, compound glycyrrhizin, adenosylmethionine, ursodeoxycholic acid, biphenyl diester, bicyclic alcohol tablets |
| Hyperlipidemia | NA | The protocol provides for blood biochemistry during the screening period; blood biochemistry is performed weekly during cycle 1 and every eight weeks beginning with cycle 2 of the study treatment period. |  TG is abnormal in the repeated administration test of preclinical SD rats of this product, and considering that drugs with similar mechanisms of action have been reported, relevant provisions are made. | When toxicity occurs, investigators may treat symptomatically based on clinical presentation. |
| Plasma protein | NA | The protocol provides for blood biochemistry during the screening period; blood biochemistry is performed weekly during cycle 1 and every eight weeks beginning with cycle 2 of the study treatment period. |  TP, ALB, A/G were abnormal in the repeated administration test of preclinical SD rats of this product, and considering that drugs with similar mechanisms of action have been reported, relevant provisions are made. |  |
| Pancreas and blood sugar | DLT is defined as the following toxic reactions that have a correlation (including definitely related and possibly related) to the study drug occurring within a single dose and within cycle 1 (35 days) of continuous dosing:  (1) Hematological toxicity:   Grade 4 neutropenia lasts > 7 days;   Grade ≥3 febrile neutropenia;   Grade 4 thrombocytopenia;   Grade 3 thrombocytopenia with bleeding;   Grade 4 anemia.  (2) Non-hematological toxicity:   Grade 4 non-hematological toxicity;   Grade 3 non-hematological toxicity that does not recover to ≤ Grade 2 within 3 days of treatment (except for simple laboratory test abnormalities that, in the judgement of the investigator, are not clinically symptomatic and do not require intervention).  (3) Other toxic reactions that, in the judgement of the investigator, require permanent discontinuation of the study drug or result in discontinuation of the first cycle for more than 7 days. |  The protocol provides for blood biochemical examination during the screening period; Blood biochemistry is performed weekly during cycle 1 and every eight weeks beginning with cycle 2 of the study treatment period. |  In the preclinical animal long toxicity test of this product, it was found that it is toxic to the pancreas (pancreatic islet cell hypertrophy/hyperplasia, pancreatic acinar cell degranulation, blood sugar increase), and there are adverse events related to drugs with similar mechanisms of action of this product, so relevant provisions are made. |  |
| Reproductive toxicity | The protocol entry criteria stipulate that subjects must meet the following criteria:  All females of childbearing potential must have a negative serum pregnancy test within 7 days prior to the first dose of study drug and both male and female subjects of childbearing potential must agree to remain abstinent or use contraception throughout the study period and for at least 3 months after the last dose of study drug. | The protocol is concerned with pregnancy reports as serious adverse events |  Preclinical long toxicity experiments observed a weight reduction in reproductive tissue, so relevant regulations are made. |  |
| Gastrointestinal tract | 1. The protocol entry criteria stipulate that subjects must meet the following criteria:   AEs of prior antineoplastic therapy have not returned to a CTCAE 5.0 grade rating of ≤ grade 1 (with the exception of toxicities judged by the investigator to be of no safety risk, e.g. alopecia, grade 2 peripheral neurotoxicity, etc.);  2. The protocol exclusion criteria state that subjects must not meet the following criteria:   Inability to swallow medication orally, or the presence of conditions that, in the judgement of the investigator, severely impair gastrointestinal absorption. | Dose adjustment:  Diarrhea treatment: At the onset of loose stools or obvious signs of diarrhea, symptomatic treatment with antidiarrheal medication is recommended. For antidiarrheal medication, if diarrhea is still grade ≥3, the medication should be discontinued and the medication should be taken at the original dosage (if the original dosage is the lowest dosage) or one dosage lower after the dosage is resumed to grade ≤1. If grade ≥3 diarrhea recurs after dose resumption, the subject will be discontinued and withdrawn from the study. Antiemetic therapy: Antiemetic therapy is not recommended for the prevention of emesis and may be administered after the onset of emesis. In the case of antiemetic therapy, if vomiting reaches grade ≥3, the medication should be discontinued and resumed at the original dose (if the original dose is the lowest dose) or reduced by one dose level after resumption of the dose to grade ≤1. If grade ≥3 emesis occurs again after resumption of dosing, the subject will discontinue dosing and withdraw from the study.. |  Some gastrointestinal toxicity (decreased food intake, loose stools, vomiting, etc.) was found in the preclinical Beagle repeat dosing trial of this product, and AEs associated with drugs with similar mechanisms of action were specified in relation to this product, so relevant provisions were made.   The current protocol is more fully described. | Antiemetic drugs may be selected but not limited to: dexamethasone, 5-HT3 receptor antagonists, or dopamine antagonists (such as metoclopramide) |
| Bleeding tendency | The protocol entry criteria state that subjects must meet the following criteria:  Coagulation function:  • PTor INR≤1.5×ULN. |  The protocol provides for four coagulation tests during the screening period; weekly coagulation tests during cycle 1 and eight-weekly coagulation tests beginning in cycle 2 of the study treatment period. |  This drug was found to have gastric mucosal reddening and ileal discolouration in preclinical SD rats and Beagle dogs in repeated dosing tests, so the relevant provisions were made. | When toxicity occurs, investigators may treat symptomatically based on clinical presentation. |
| kidney | The protocol entry criteria state that subjects must meet the following criteria:  Kidney function:  Creatinine clearance ≥ 50 mL/min. | The protocol provides for blood biochemistry during the screening period; weekly blood biochemistry during cycle 1 and eight-weekly blood biochemistry during cycle 2 of the study treatment period. |  This drug is prescribed in view of the preclinical findings of elevated urea in the acute toxicity study in SD rats and elevated creatinine in the repeat dose study in SD rats and the fact that drugs with similar mechanisms of action have been reported in this regard, so relevant provisions are made. | Hepatoprotective treatment can be selected but not limited to: urotoxin, Shen Shuaining, Shen fukang, paraformaldehyde starch , kaito tablet, Bailing capsule. |
